# Increasing Complexity of the N-Glycome During *Caenorhabditis* Development

**DOI:** 10.1016/j.mcpro.2023.100505

**Published:** 2023-01-28

**Authors:** Iain B.H. Wilson, Shi Yan, Chunsheng Jin, Zuzanna Dutkiewicz, Dubravko Rendić, Dieter Palmberger, Ralf Schnabel, Katharina Paschinger

**Affiliations:** 1 Department für Chemie, Universität für Bodenkultur, Wien, Austria; 2 Institut für Parasitologie, Veterinärmedizinische Universität Wien, Wien, Austria; 3 Institutionen för Biomedicin, Göteborgs universitet, Göteborg, Sweden; 4 Institut für Genetik, Technische Universität Braunschweig, Braunschweig, Germany

**Keywords:** glycomics, mass spectrometry, galactose, fucose, phosphorylcholine, ESI, electrospray ionization, FUT, fucosyltransferase, GALT, galactosyltransferase, g.u., glucose unit, HIAX, hydrophilic interaction anion exchange, MS, mass spectrometry, PC, phosphorylcholine, PCE, phosphorylcholine esterase, PNGase, peptide:N-glycosidase, RP, reversed-phase

## Abstract

*Caenorhabditis elegans* is a frequently employed genetic model organism and has been the object of a wide range of developmental, genetic, proteomic, and glycomic studies. Here, using an off-line MALDI-TOF-MS approach, we have analyzed the N-glycans of mixed embryos and liquid- or plate-grown L4 larvae. Of the over 200 different annotatable N-glycan structures, variations between the stages as well as the mode of cultivation were observed. While the embryonal N-glycome appears less complicated overall, the liquid- and plate-grown larvae differ especially in terms of methylation of bisecting fucose, α-galactosylation of mannose, and di-β-galactosylation of core α1,6-fucose. Furthermore, we analyzed the O-glycans by LC–electrospray ionization–MS following β-elimination; especially the embryonal O-glycomes included a set of phosphorylcholine-modified structures, previously not shown to exist in nematodes. However, the set of glycan structures cannot be clearly correlated with levels of glycosyltransferase transcripts in developmental RNA-Seq datasets, but there is an indication for coordinated expression of clusters of potential glycosylation-relevant genes. Thus, there are still questions to be answered in terms of how and why a simple nematode synthesizes such a diverse glycome.

## Highlights

• *Caenorhabditis* embryos express a simpler N-glycome than L4 larvae.
• Liquid-grown L4 larvae have more methylated N-glycans.
• Plate-grown L4 larvae possess more variable core chitobiose modifications.
• Transcriptomic data indicate clustered expression of glycosyltransferases.

## Graphical Abstract

**Figure:**
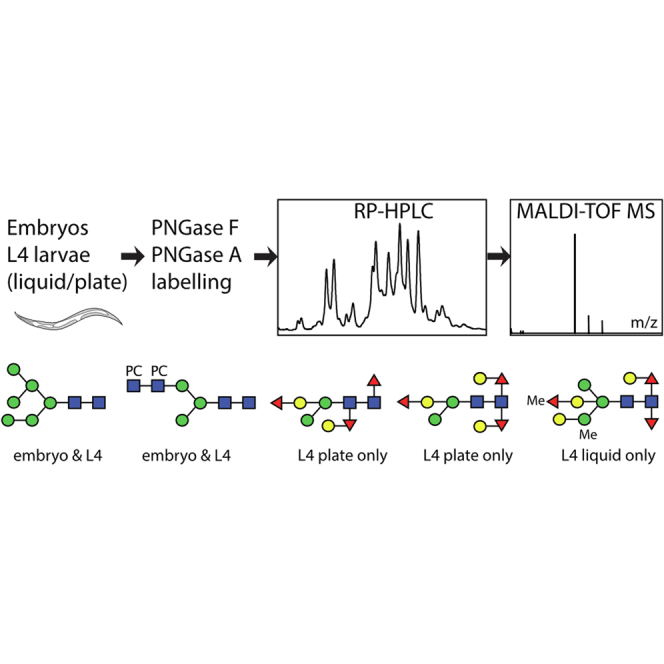

## In Brief

There is an increasing N-glycomic complexity during development of the nematode, *Caenorhabditis elegans*, as revealed by off-line HPLC/MALDI-TOF-MS/MS. The higher degree of glycan methylation and α-galactosylation of mannose residues in liquid-grown worms may reflect cultivation-dependent stress. Furthermore, phosphorylcholine modifications were found not just on N-glycans but on O-glycans. The increased branching and core fucosylation of N-glycans in the larvae as compared with the embryos may correlate with regulated expression of key glycosyltransferases.

As multicellular organisms develop, it can be expected that post-translational modifications of their proteins vary, especially those involved in cell–cell interactions. Indeed, there is much evidence to indicate that protein-linked glycans are developmentally highly relevant (1). Variations in glycosylation are dependent on the expression of the proteins to which they are attached, the enzymes that modify them, and the availability of the necessary nucleotide sugar donors. In invertebrates, examples of developmental glycomic alterations as detected by mass spectrometry (MS) include reports on insects (2), nematodes (3) and trematodes (4).

*Caenorhabditis elegans* is the first multicellular organism to have its genome sequenced (5). The developmental fate of the cells from embryo to adult are well described (6) and reports have previously concluded that there are differences in N-glycosylation between embryos, larvae, and adults (7, 8). Many of the earlier studies on *C. elegans* N-glycans have centered on the comparison of wildtype and mutant glycomes, including the triple GlcNAc-TI-knockout (9, 10) and various fucosyltransferase (FUT) or hexosaminidase single, double, and triple knockouts (11, 12, 13, 14, 15), as well as mutants of the Golgi mannosidase II *aman-2*, galactosyltransferase *galt-1*, GDP-Man dehydratase *bre-1*, UDP-hexose transporter *srf-3*, conserved oligomeric Golgi complex *cogc-1*, and putative *S*-adenosylmethionine transporter *samt-1* genes (16, 17, 18, 19, 20, 21). An overall consensus as to the N-glycosylation capacity of *C. elegans* was slow to emerge, but the overall complexity soon became obvious as well as a significant (22, 23), but not complete, overlap with the N-glycomes of parasitic nematodes (24). Especially the high degree of modification of the N-glycan core region is a novel feature of *C. elegans* (25, 26). In terms of mucin-type O-glycans, in addition to NMR-based data (27), comparisons have been made between the wildtype and the *bre-1*, *bus-2*, *bus-4*, *samt-1*, and *srf-3* mutants, also indicative of unusual structures, including ones modified by glucuronic acid (18, 19, 20, 28, 29).

Using an off-line LC–MS approach, we have now performed an in-depth N-glycomic analysis of the mixed embryos and larval L4 stages, the latter grown either in liquid or on plates. Building on previous MALDI-TOF MS, electrospray ionization (ESI)–MS, GC–MS, and NMR data on the range of N-glycan motifs found in mutant *C. elegans* strains (11, 12, 13), we revealed over 200 different N-glycan structures with variations in fucosylation and galactosylation or the degree of antennal modifications, including complex forms not found to date. Furthermore, on-line LC–MS of the O-glycans of *C. elegans* shows, for the first time, the presence of phosphorylcholine-modified mucin-type oligosaccharides in a nematode, in addition to the previously found zwitterionic N-glycan, glycolipid, and glycosaminoglycan-type structures.

## Experimental Procedures

### Biological Material and Glycan Preparation

Wildtype *C. elegans* (N2) and *pmk-1(km25)* strains were obtained from the *Caenorhabditis* Genetics Centre, University of Minnesota. The *apx-1(t3208)* and *glp-1(e2144)* mutants with altered cell identities were previously isolated (30); *apx-1* is the abbreviation for anterior pharynx in excess and encodes a delta-like ligand for the Notch receptor encoded by the *glp-1* gene, the abbreviation for germ line proliferation mutant. Synchronized larvae were grown either in liquid culture or on nematode growth medium agar plates and harvested at the L4 stage. All worm materials were boiled in water for 10 min to heat-inactivate hydrolases prior to glycan preparation. *C. elegans* eggs containing mixed-stage embryos (2 g, prior to the terminal differentiation) were ground in liquid nitrogen in a mortar, whereas the L4 larvae (both batches, 2–3 g) were homogenized in a glass homogenizer. The homogenates were transferred into glass flasks, adjusted to 5% (v/v) formic acid and proteolyzed overnight at 37 °C (porcine pepsin, Sigma; 1 mg per gram of worm sample). Glycopeptides were purified by cation exchange chromatography (Dowex 50W×8; elution with 0.5 M ammonium acetate, pH 6.0) followed by G25 gel filtration (31).

In the case of the embryos, peptic glycopeptides were subject to glycan release using peptide:N-glycosidase (PNGase) A alone (recombinant, prepared in house; Supplementary Data) in 50 mM ammonium acetate buffer (pH 5.0). For the different L4 larval preparations, the glycopeptides were subject to sequential release using two different PNGases: recombinant bacterial PNGase F (from *Flavobacterium* [*Elizabethkingia*] *meningosepticum*, Roche; at pH 8.0) and either a native almond PNGase A (Roche, used in the first batch) or a recombinant rice PNGase Ar (from *Oryza sativa* expressed in *Pichia pastoris* [*Komagataella phaffii*] and Endo H treated; New England Biolabs; at pH 5.0; used in the second batch). First, the L4 glycopeptides were treated with PNGase F overnight at 37 °C. After cation exchange chromatography on Dowex 50W×8, native glycans were purified stepwise using nPGC and C18 cartridges. Glycan-containing fractions, as judged by MALDI-TOF MS, were fluorescently labeled with 2-aminopyridine. The remaining L4 glycopeptides bound to the Dowex resin were eluted with 0.5 M ammonium acetate and gel filtrated (Sephadex G25) prior to incubation with either PNGase A or PNGase Ar overnight at 37 °C. The N-glycans (including those carrying core α1,3-fucose) released at this stage were also then no longer bound by Dowex and were subject to the same purification and fluorescent-labeling steps as for those released with PNGase F. Residual glycopeptides of embryos remaining after PNGase A treatment and glycopeptides of L4 larvae remaining after serial PNGase F and A treatment (first batch) were subject to O-glycan release by reductive β-elimination prior to LC–MS analysis (32).

### N-Glycan Fractionation

Pyridylaminated N-glycome pools were fractionated by reversed-phase (RP) HPLC (Hypersil ODS 250 × 4.6 mm C18 column; Agilent), and a gradient of 30% (v/v) methanol (buffer B) in 100 mM ammonium acetate, pH 4 (buffer A), was applied at a flow rate of 1.5 ml/min as follows: 1% buffer B per minute over 35 min. Lyophilized HPLC fractions were dissolved in water and individually subject to MALDI-TOF MS. The RP-HPLC column was calibrated daily in terms of glucose units (g.u.) (33) using a pyridylaminated dextran hydrolysate (2–20 g.u.), and the degree of polymerization of single standards was verified by MALDI-TOF MS.

Alternatively, aliquots of the plate-grown L4 larval glycan pools (PNGase F or Ar released) were subject to hydrophilic interaction anion exchange (HIAX) HPLC for size/charge separation followed by RP-amide HPLC. HIAX was performed with an IonPac AS11 column (Dionex; 4 × 250 mm) using a Shimadzu Nexera UPLC system as described previously (34). A two-solvent gradient was applied with buffer A (0.8 M ammonium acetate, pH 3.85) and buffer B (80% acetonitrile) at a flow rate of 1 ml/min: 0 to 5 min, 99% B; 5 to 50 min, 90% B; 50 to 65 min, 80% B; 65 to 85 min, 75% B. A pool of pyridylaminated oligomannosidic N-glycans from white beans (containing Man_3–9_GlcNAc_2_) was used to calibrate the column. Selected HIAX fractions were then applied to an Ascentis Express RP-amide column (Sigma–Aldrich; 150 × 4.6 mm, 2.7 μm), and a gradient of 30% (v/v) methanol (buffer B) in 100 mM ammonium acetate, pH 4 (buffer A) was applied at a flow rate of 0.8 ml/min (Shimadzu LC-30 AD pumps) as follows: 0 to 4 min, 0% B; 4 to 14 min, 0 to 5% B; 14 to 24 min, 5 to 15% B; 24 to 34 min, 15 to 35% B; 34 to 35 min, return to starting conditions (35). The RP-amide HPLC column was calibrated daily in terms of glucose units using a pyridylaminated dextran hydrolysate.

### MALDI-TOF MS

Free glycans and pyridylaminated glycans were analyzed in positive ion mode using a Bruker Autoflex Speed instrument (1000 Hz Smartbeam-II laser) and 6-aza-2-thiothymine as matrix; calibration was performed using a Bruker peptide standard. MS/MS of [M + H]^+^ ions was performed by laser-induced dissociation (precursor ion selector was generally ±0.6%). The detector voltage was normally set at 1977 V for MS and 2133 V for MS/MS; 1000 to 2000 shots from different regions of the sample spots were summed. Spectra were processed with the manufacturer’s software (Bruker Flexanalysis 3.3.80) using the SNAP algorithm with a signal/noise threshold of six for MS (unsmoothed) and three for MS/MS (four times smoothed). Glycan MS and MS/MS spectra (approximately 5500 in total) were manually interpreted on the basis of the masses of the predicted component monosaccharides, the differences of mass in glycan series, fragmentation patterns, and results of enzymatic and chemical treatments. For the approximately 200 proposed structures (supplemental Table S1), the minimum criterion for inclusion was an interpretable MALDI-TOF MS/MS spectrum (see also mzxml raw data files). Furthermore, examples for each core and antennal motif were verified by digestion data; comparison was also made to elution, in terms of glucose units, with previous data. For bisecting and distal core GlcNAc modifications, corroborative evidence comes from ESI–MS^2^, GC–MS, and/or NMR data of N-glycans from mutant *C. elegans* strains with simplified N-glycomes (11, 12, 13, 26), whereas phosphorylcholine and core difucosylation are known modifications of nematode and insect N-glycans (36, 37, 38, 39, 40, 41); the occurrence of 2-*O*-methylfucose and 3-*O*-methylmannose residues has also been demonstrated in *C. elegans* (18). Calculated theoretical masses were verified using GlycoWorkbench 2.0 (EurocarbDB). The deviation between calculated and observed *m/z* values was typically 0.1 to 0.2 Da.

### Exoglycosidase and Hydrofluoric Acid Treatment

Aliquots of the isolated HPLC fractions were, based on results of HPLC elution and MALDI-TOF MS and MS/MS data, subject to targeted exoglycosidase digestion and chemical treatment (42). Either α-fucosidase (microbial α1,2-specific FUCM from Megazyme), α-galactosidase (coffee bean from Sigma, desalted (43)), β-galactosidase (recombinant, *Aspergillus nidulan*s or *Aspergillus niger* (44)), β-hexosaminidases (recombinant *C. elegans* HEX-4 prepared in-house (45), *Streptomyces plicatus* chitinase from New England Biolabs (46), or jack bean hexosaminidase from Sigma (47)), α-mannosidase (jack bean from Sigma, jack bean from New England Biolabs, or *Xanthomonas* α1,2/3-mannosidase from New England Biolabs (48, 49)), or recombinant *Streptococcus pneumoniae* phosphorylcholine esterase (PCE) (50, 51), were used for further treatment of the sample in 25 mM ammonium acetate, pH 5 (pH 6.5 in the case of FUCM or HEX-4), at 37 °C for 24 h. For chemical removal of phosphorylcholine or α1,3-fucose residues, selected fractions were dried and incubated for 48 h at 0 °C with 3 μl 48% (v/v) hydrofluoric acid prior to evaporation in a centrifugal concentrator. The samples were diluted in water and re-evaporated, before redissolving once again. The chemically or enzymatically treated fractions were subject to MALDI-TOF MS and MS/MS (as aforementioned) without further purification, except for the PCE digests, which were subject to solid phase extraction on C18 and elution with 30% (v/v) MeOH.

### LC–ESI MS

O-glycans were analyzed by online LC–MS/MS using a 10 cm × 150 μm I.D. column, prepared in-house, containing 5 μm porous graphitized carbon particles coupled to an LTQ ion trap mass spectrometer (Thermo Scientific). Glycans were eluted using a linear gradient from 0 to 40% acetonitrile in 10 mM ammonium bicarbonate over 40 min at a flow rate of 10 μl/min. The eluted O-glycans were detected in negative-ion mode with an electrospray voltage of 3.5 kV, capillary voltage of −33.0 V, and capillary temperature of 300 °C (52). Specified ions were isolated for MS^n^ fragmentation by collision-induced dissociation with the collision energy set to 30%. Air was used as a sheath gas, and mass ranges were defined dependent on the specific structure to be analyzed. The data were processed using the Xcalibur software (version 2.0.7; Thermo Scientific). O-glycans were identified from their MS/MS spectra by manual annotation (supplemental Table S2).

### Bioinformatic Analyses

A reference protein set (RefSeq) for *C. elegans* was downloaded as a fasta file from National Center for Biotechnology Information GenBank on September 14, 2013. The whole fasta file was uploaded for analysis to the CBS TMHMM Server, version 2.0: http://www.cbs.dtu.dk/services/TMHMM/. “One line per protein” was selected as an output format, and the data were saved in a text file, extensive empty spaces were trimmed, and loaded into Excel. Thereafter, all entries predicted to have more than one transmembrane domain were deleted as were those not predicted to have the transmembrane domain near either N or C terminus; finally, sequences of less than 300 or more than 600 amino acids were excluded, other than known glycobiosynthetic enzymes. The corresponding Wormbase accessions and gene names (if assigned) for selected National Center for Biotechnology Information entries were then retrieved. Using the time-resolved RNA-Seq data (53) available *via* GExplore (http://genome.sfu.ca/gexplore/gexplore_search_all.html), which contains whole transcriptome data from *C. elegans* (54), the relevant differential pulse code modulation values were then saved into a csv file, analyzed using R scripts, and plotted using the pheatmap library (https://www.r-project.org). For correlation of the expression levels of potential glycosylation-relevant genes, the R corrplot library was used based on a previously published approach (55).

## Results

### N-Glycans of *C. elegans* Embryos

**Fig. 1:**
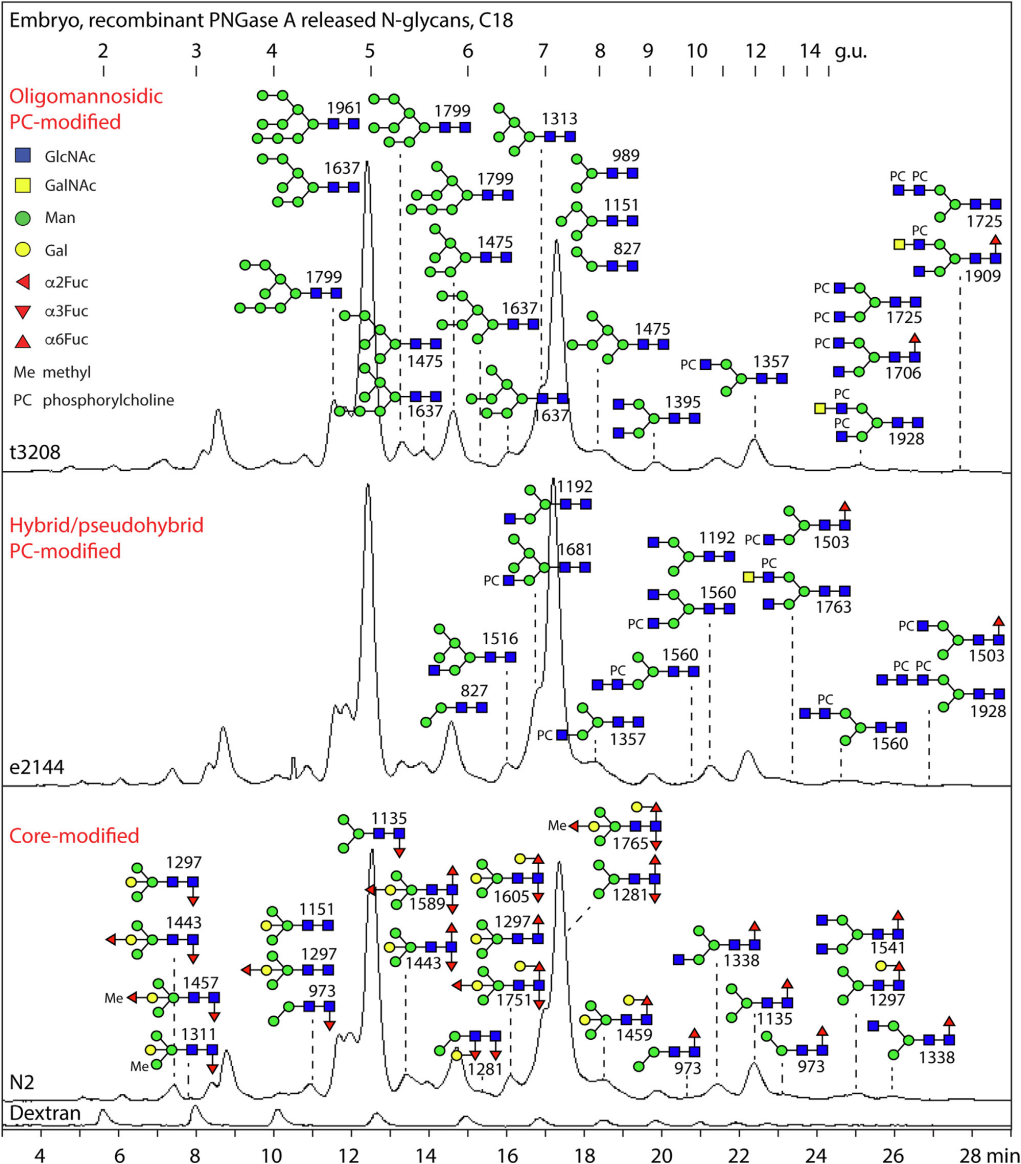
**RP-HPLC of PNGase A-released pyridylaminated N-glycans from *Caenorhabditis elegans* embryos.** Chromatograms for the wildtype N2, mutant e2144, and t3208 strains are shown annotated with dextran hydrolysate as external calibrant (in glucose units, g.u.) and with the structures found on the basis of MS, MS/MS, and digestion data; glycans are depicted according to the Standard Nomenclature for Glycans as shown. There are only minor differences in the N-glycans detected in the three strains, for example, some core α1,3-fucosylated Hex_3–5_HexNAc_2_Fuc_1–3_ structures were not detected in the mutants. For clarity, the oligomannosidic structures are shown in the *upper panel* only, the phosphorylcholine-modified ones in the *upper and middle panels*, and the various core-modified paucimannosidic ones in the *lower panel*. See supplemental Fig. S2 for MS of individual fractions of N-glycans from N2 embryos and supplemental Table S1 for a full list. Previous studies have shown that different pyridylaminated Man_6–8_GlcNAc_2_ and Man_2–3_GlcNAc_2_Fuc_1_ isomers have distinct RP-HPLC retention times and fragmentation patterns (33, 35, 87). PNGase, peptide:N-glycosidase; RP, reversed-phase.

Initially, we assessed whether there were differences between the N-glycomes of wildtype *C. elegans* (N2) and two mutants with minor defects in development, specifically *apx-1* (*t3208*) and *glp-1* (*e2144*). While GLP-1 encodes a Notch-type receptor, APX-1 functions as a GLP-1 ligand, thereby mediating cell–cell interactions in a Notch signaling pathway (56). Both wildtype and *glp-1* embryos from liquid culture were previously analyzed by MALDI-TOF MS alone after PNGase A release from tryptic peptides (57). Here, we apply our off-line HPLC-MALDI-TOF-MS/MS workflow using our own recombinant PNGase A to release glycans (supplemental Fig. S1) from pepsin-generated peptides. All three RP-HPLC chromatograms are highly similar with only minor differences in peak shape (Fig. 1). In terms of the glycans in each peak (supplemental Table S1 and supplemental Fig. S2), only 10 minor structures were not observed in all strains; however, the apparent absence of a minor structure may be merely because of sensitivity limitations.

The embryo N-glycomes of all three strains were rich in oligomannosidic structures, proven by retention time and, in some cases, α-mannosidase treatment. A number of monofucosylated and difucosylated glycans and three trifucosylated paucimannosidic glycans were present in all three strains; in comparison to other studies (35), the type of core fucosylation (α1,3 or α1,6) was partly defined because of hydrofluoric acid sensitivity (α1,3) or resistance (α1,6) as well as the Y_1_ ion fragments of *m/z* 446, 592, 608, or 754 (HexNAc_1_Fuc_1–2_Gal_0–1_-PA; supplemental Fig. S2), whereby fucosylation of the distal core GlcNAc was rare. Two different positions for β-galactosylation could be defined, either bisecting or on the core α1,6-fucose, as previously reported in mutant or wildtype adult *C. elegans* (11, 12, 13), but α-galactosylation could not be demonstrated; methylation was rather limited. Some hybrid, pseudohybrid, or biantennary structures were found but none with three antennae. The late-eluting glycans tend to be modified with phosphorylcholine on the antennae (all with the key MS/MS fragment B_1_ ion of *m/z* 369, corresponding to PC_1_HexNAc_1_; supplemental Fig. S2), whereby sensitivity to the GalNAc-specific HEX-4 hexosaminidase was observed for a glycan with a PC_1_HexNAc_2_ motif. Overall, we conclude that there are no significant differences in the mutant embryonal N-glycomes; embryos are also less rich in terms of glycan complexity as compared with the L4 larvae, because of lower variability of the core and antennal modifications, with about 60 structures detected in the former as compared with a total of 120 in the latter.

### N-Glycans of *C. elegans* L4 Larvae

Considering previous data suggesting that wildtype embryos grown in liquid or plate cultures differed in terms of their N-glycomes (57), we harvested L4 worms grown under both these conditions and prepared N-glycans *via* serial digestion with PNGase F followed by either native PNGase A (first preparation) or recombinant PNGase Ar (second preparation; performed to confirm the data from the first preparations and to use an enzyme with a broader specificity). The resulting pools of glycans were analyzed by the off-line RP-HPLC-MALDI-TOF-MS/MS workflow; also aliquots of the plate-grown L4 PNGase F- and Ar-released N-glycans were subject to size-based HIAX fractionation followed by RP-amide HPLC on two selected pools.

**Fig. 2:**
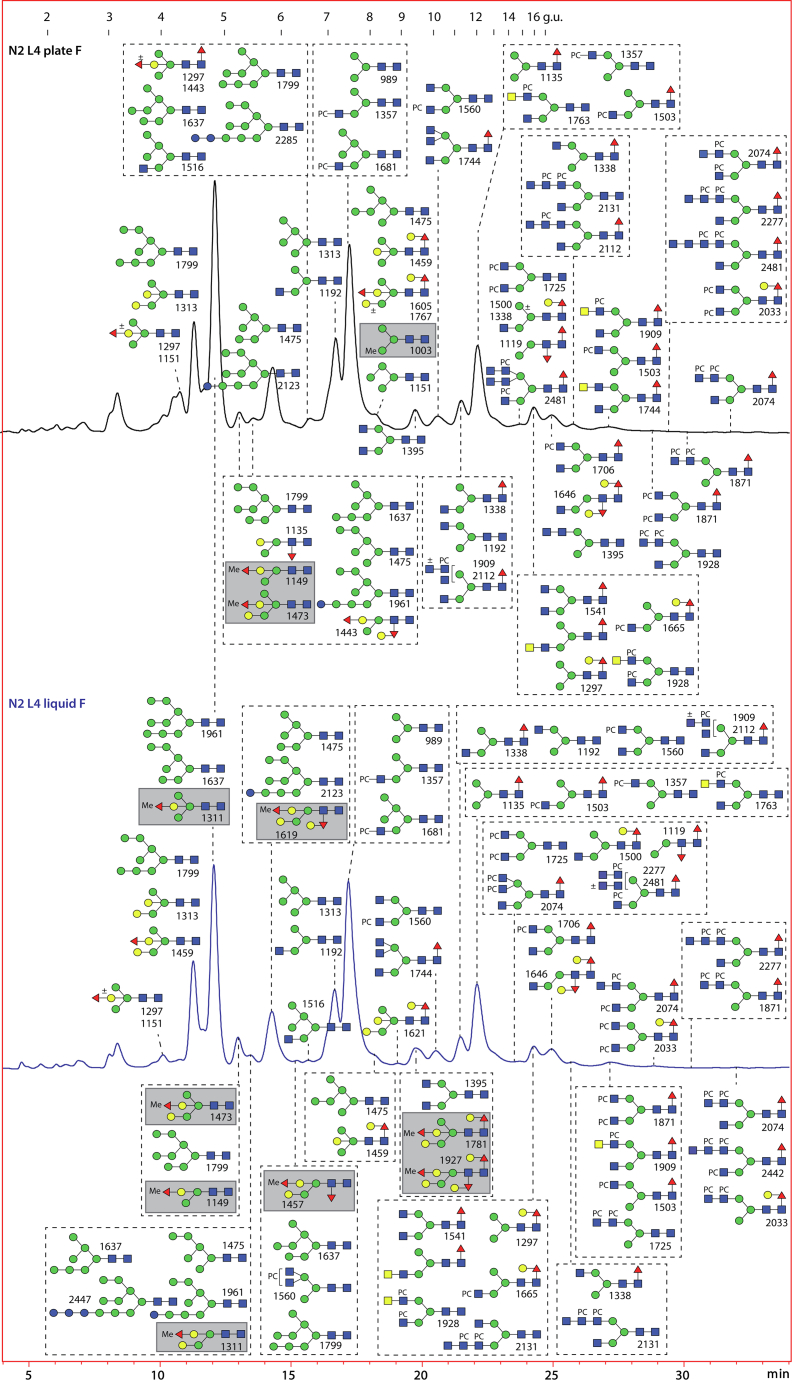
**RP-HPLC of PNGase F-released pyridylaminated N-glycans from *Caenorhabditis elegans* L4 larvae cultivated in liquid or on plates.** Chromatograms for the wildtype L4 liquid- and plate-grown larvae are shown annotated with dextran hydrolysate external calibrant (in glucose units, g.u.) and with the structures found on the basis of MS, MS/MS, and digestion data. Native methylated glycans (highlighted with *gray boxes*) are more frequent in the liquid-grown larvae. See Figures 3 and 4 for MS/MS and digestion data for example glycans, supplemental Fig. S3 for chromatograms of independent preparations, and supplemental Fig. S5 for 2D-HPLC of the plate-grown larvae glycome. PNGase, peptide:N-glycosidase; RP, reversed-phase.

**Fig. 3:**
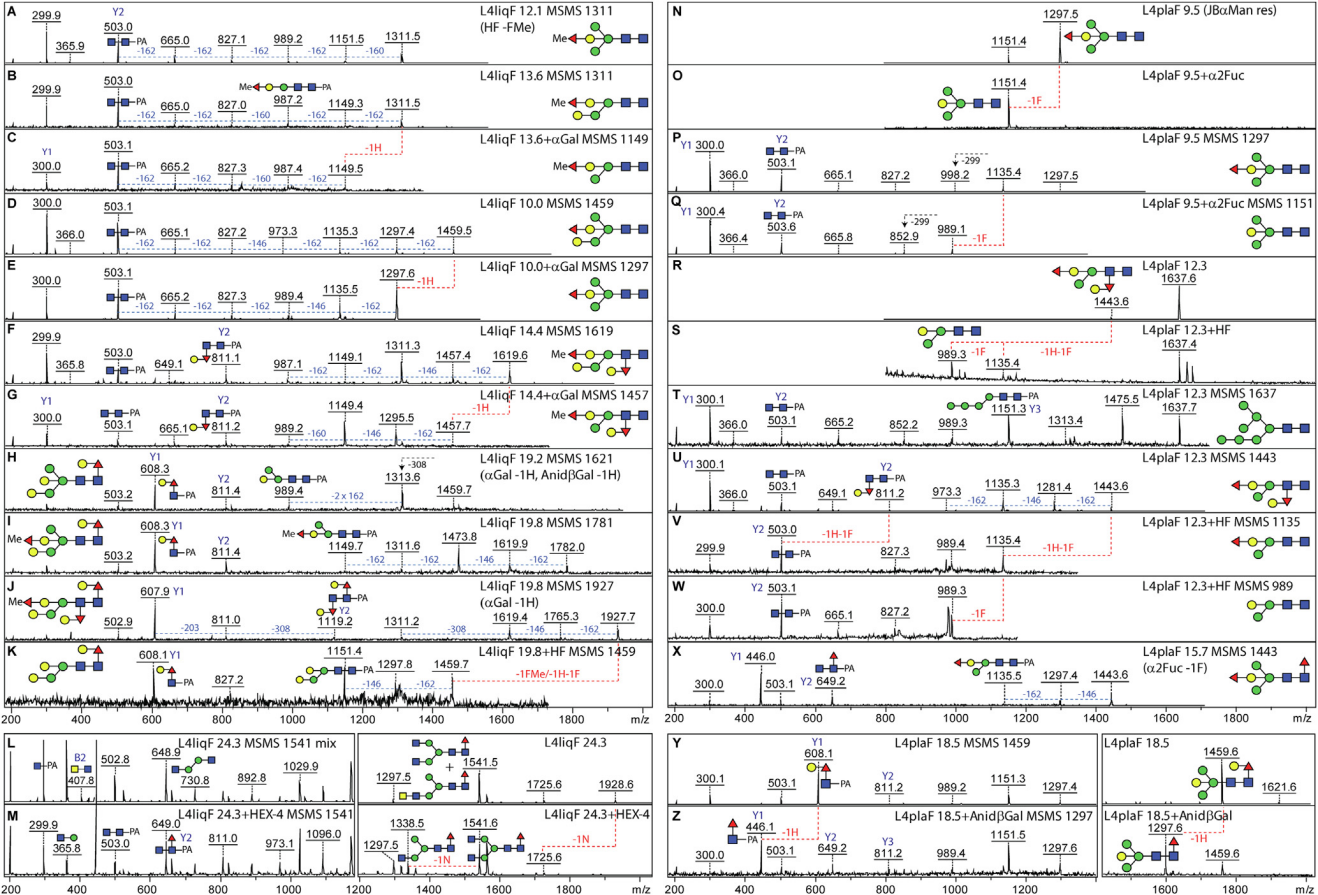
**Example of MS/MS and digestion data for PNGase F-released N-glycans from *Caenorhabditis elegans* L4 larvae.** RP-HPLC fractionated glycans were subject to MALDI-TOF MS and MS/MS in positive mode before and after chemical or enzymatic treatment; each glycan is annotated with the fraction name (L4liqF or L4plaF and the retention time in minutes), the *m/z*, the proposed structure, key Y_1_ or Y_2_ fragment ions, and sensitivity to α- or β-galactosidase, α1,2-fucosidase, HEX-4 β1,4-*N*-acetylgalactosaminidase, or hydrofluoric acid (HF), resulting in the indicated losses of fucose (F), hexose (H), or *N*-acetylhexosamine (N). *A*–*K*, MS/MS spectra for glycans from larvae cultivated in liquid before or after coffee bean α-galactosidase (αGal) or HF treatment; shown are data for two isomers of *m/z* 1311 and single isomers of *m/z* 1459, 1619, 1621, 1781, and 1927, which display differences in the occurrence of α-galactosylation, α1,2-fucosylation, methylation, and core fucosylation; as appropriate, losses of 146 (Fuc), 160 (MeFuc), 162 (Hex), 299 (HexNAc_1_-PA), or 308 Da (Hex_1_Fuc_1_) are indicated. *L* and *M*, MS and MS/MS for two coeluting forms of Hex_3_HexNAc_4_Fuc_1_ (*m/z* 1541); treatment with the *C. elegans* HEX-4 β-*N*-acetylgalactosaminidase resulted in partial conversion to *m/z* 1338 and a loss of the trace *m/z* 407 (LacdiNAc) B_2_ fragment ion; the HEX-4-sensitive *m/z* 1928 glycan in this fraction is PC modified (Fig. 4*D*). *N*–*X*, MS and MS/MS spectra for glycans from larvae cultivated on plate before or after microbial α1,2-fucosidase (α2Fuc) or HF treatment; shown are data for a simple bisected glycan of *m/z* 1297 and a bisected glycan of *m/z* 1443 with a modified distal GlcNAc, the latter coeluting with an HF-resistant Man_7_GlcNAc_2_ structure of *m/z* 1637, as well as a core fucosylated isomer of *m/z* 1443. *Y* and *Z*, MS and MS/MS for an isoform of Hex_5_HexNAc_2_Fuc_1_ (*m/z* 1459) with a “GalFuc” epitope on the proximal core GlcNAc; treatment with *Aspergillus nidulans* β-galactosidase (βGal) resulted in conversion to *m/z* 1297 and replacement of the *m/z* 608 Gal_1_Fuc_1_GlcNAc_1_-PA Y_1_ fragment ion by one at *m/z* 446. A lack of such *m/z* 446 Y_1_-fragments (Fuc_1_GlcNAc_1_-PA) for monofucosylated and difucosylated glycans is indicative of an unmodified proximal GlcNAc. HF-sensitive distal GalFuc modifications (*F*, *G*, and *U*) are defined by the presence of *m/z* 811 Y_2_ and the absence of *m/z* 608 Y_1_ MS/MS fragments; this modification has been characterized by GC–MS and ESI–MS^2^ (11, 26). Bisecting β-galactose (with or without fucose or methylated fucose) is a motif previously defined by serial chemical/enzymatic digestion, ESI–MS^2^ and NMR in *C. elegans* double *fut-1;fut-6* and triple *fut-1;fut-6;fut-8* knockout strains lacking two or three chitobiose core-modifying α-fucosyltransferases (12, 13). ESI, electrospray ionization; PNGase F, peptide:N-glycosidase; RP, reverse-phase.

The chromatograms of the PNGase F-released glycans for liquid- and plate-grown L4 larvae were rather similar, also regardless of preparation (Figs. 2, S3 and S4). Based on MS/MS and chemical or enzymatic treatments, some 60 to 75 structures per PNGase F-released glycome were annotated (supplemental Table S1), with two-thirds being common to all samples. The most obvious differences appeared to be (i) the relative lack of α-galactosylated and/or methylated structures in the plate-grown L4 PNGase-F released glycomes and (ii) some sample-dependent variations in minor phosphorylcholine-modified glycans. The major structures in the corresponding most abundant fractions were shared between all L4 PNGase F-released samples, including the typical paucimannosidic and oligomannosidic glycans. Example digestion and MS/MS data for neutral structures are shown in Figure 3 and demonstrated the presence of structures galactosylated on fucose or mannose residues as reported previously in mixed cultures with primarily adults (26), as proven by α- and β-galactosidase treatments (Fig. 3, *C*, *E*, *G* and *Z*); fucose was not only just a core modification but also a substitution of the bisecting β1,4-galactose residues sensitive to either α1,2-fucosidase or hydrofluoric acid (Fig. 3, *N*–*S* and *U*–*W*). Fucose on the bisect, but not the core, could also be methylated (Fig. 3, *A*–*C*, *F*, *I*–*K*). Furthermore, a neutral glycan containing a LacdiNAc motif was also detected, and the diagnostic *m/z* 407 B-ion was absent after HEX-4 β-*N*-acetylgalactosaminidase treatment (Fig. 3, *L* and *M*).

**Fig. 4:**
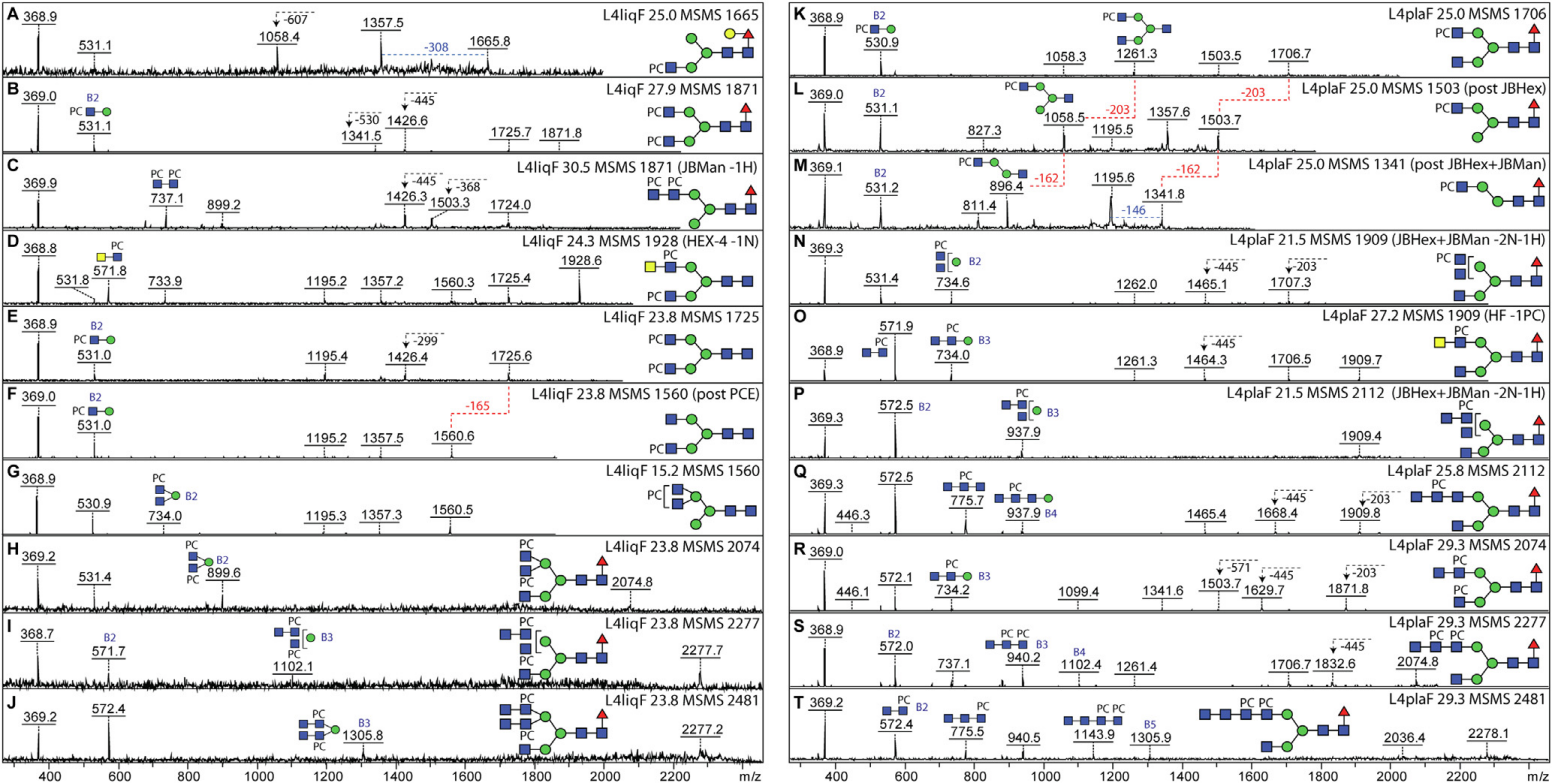
**Example of MS/MS and digestion data for PNGase F-released zwitterionic N-glycans from *Caenorhabditis elegans* L4 larvae.** RP-HPLC fractionated phosphorylcholine (PC)-modified glycans isolated from liquid- or plate-cultivated larvae were subject to MALDI-TOF MS and MS/MS in positive mode before and after enzymatic treatment; the MS/MS spectra are annotated with the fraction name (L4liqF or L4plaF and the retention time), the *m/z* value, key B fragments, and sensitivity to jack bean α-mannosidase, jack bean β-hexosaminidase, HEX-4 β1,4-*N*-acetylgalactosaminidase, or phosphorylcholine esterase (PCE), resulting in indicated losses of hexose (H, 162 Da), *N*-acetylhexosamine (N, 203 Da), or PC (165 Da); a loss of 607 Da (*A*) correlates with the presence of a reducing-terminal GalFuc modification. Variations in the B fragment ions are indicative of differences in the location and number of the PC moieties (see, *e.g.*, isomers of *m/z* 1871, 1909, 2074, 2277, 2481; *B*, *C*, *H*–*J*, *N*, *O*, and *R*–*T*), whereas efficient removal of a mannose residue after combined β-*N*-acetylhexosaminidase/α-mannosidase (JBHex/JBMan) treatment is indicative that the nonmodified HexNAcHex is α1,3-linked to the core β-mannose (*K*–*M*). Terminal HexNAc in the context of a HexNAc_2–4_PC_1–2_ motif is not sensitive to jack bean β-*N*-acetylhexosaminidase (*N* and *P*), but in one case, it was removed by HEX-4 β-*N*-acetylgalactosaminidase (*D*, *m/z* 1928; Fig. 3, *L* and *M*). Whereas HF quantitatively removes PC residues, phosphorylcholine esterase (PCE) is only partially efficient (*E* and *F*). Based on previous NMR, GC–MS, and Q-TOF CAD-MS/MS data on nematode glycans, the phosphorylcholine residues are assumed to substitute the C6 of GlcNAc residues (41, 74), which are either nonreducing terminal GlcNAc or within 4-linked HexNAc-based chito-oligomer and LacdiNAc motifs (39). PNGase F, peptide:N-glycosidase; RP, reverse-phase.

A particular challenge was to assign some of the low abundance zwitterionic structures, which are late-eluting on the C18 column. Some structures are relatively simple with single phosphorylcholine-modified antennal GlcNAc residues as widely reported for nematodes (Hex_1_HexNAc_1_PC_1_ B_2_ fragments at *m/z* 531; Fig. 4, *A*, *B*, *D*, *E*, *K* and *R*); nevertheless, it is clear that *C. elegans* also synthesizes extended complex, hybrid, and pseudohybrid isomers modified with phosphorylcholine, with detected masses of up to 2500 Da. Complicated triantennary examples, especially in the liquid-cultured worms, contain branched motifs consisting of a mannose and two or more HexNAc residues, of which one or two were monosubstituted with phosphorylcholine, resulting in B-ion fragments of *m/z* 734, 899, 937, 1102, or 1305 (Hex_1_HexNAc_2–4_PC_1–2_; Fig. 4, *G*–*J*, *N* and *P*). Furthermore, there are examples of linear motifs containing two, three, or four HexNAc and one or two phosphorylcholine residues resulting in fragments of *m/z* 572, 737, 940, or 1143 detected in both liquid- and plate-grown worms (HexNAc_2–4_PC_1–2_; Fig. 4, *C*, and *O*–*T*). To target the structures with two phosphorylcholine moieties, a 2D-HPLC approach was also employed, which verified the occurrence of isomeric forms with different antennal lengths (supplemental Fig. S5). To gain insights as to the exact structure, either as noted or shown, selected fractions were subject to β-hexosaminidase (jack bean hexosaminidase, chitinase, and HEX-4), α-mannosidase, hydrofluoric acid, or PCE treatments, revealing which antennae were modified by phosphorylcholine.

**Fig. 5:**
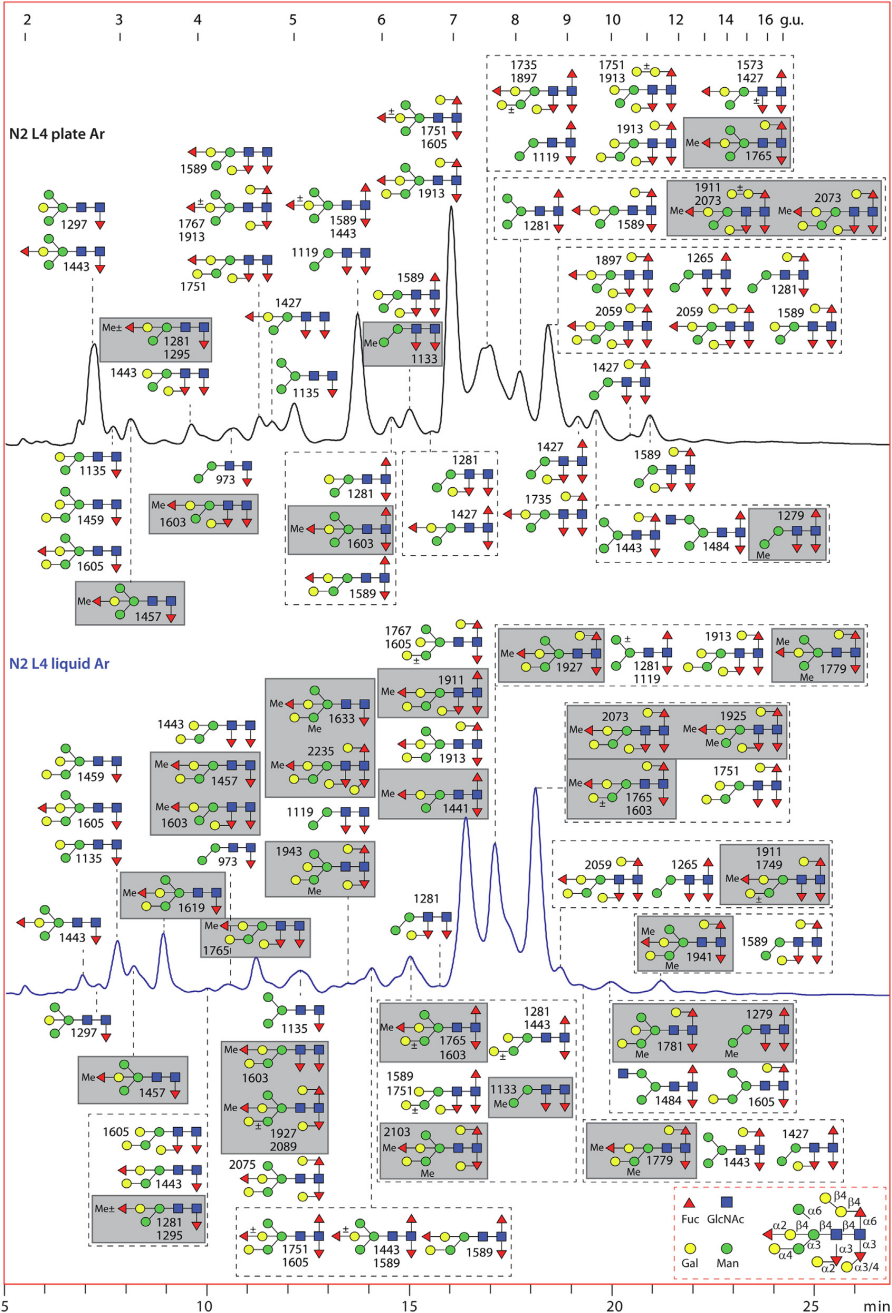
**RP-HPLC of PNGase Ar-released pyridylaminated N-glycans from *Caenorhabditis elegans* L4 larvae cultivated in liquid or on plates.** Chromatograms for the wildtype L4 liquid- and plate-grown larvae are shown annotated with dextran hydrolysate as external calibrant (in glucose units, g.u.) and with the structures found on the basis of MS, MS/MS, and digestion data. The key (*bottom right*) indicates linkages proposed from previous LC- or GC–MS data (11, 25, 26). Residual oligomannosidic and paucimannosidic glycans found in the PNGase F digests are not annotated on these chromatograms. There are approximately three times more structures with α-galactosylation of mannose and/or methylation in the liquid-grown glycome as compared with the plate-grown (34 and 30 compared with 9 and 10), suggestive of a stress-related glycomic shift. See Figures 6 and 7 and S9–S11 for example of MS/MS and digestion data, supplemental Fig. S6 for chromatograms of independent preparations, and supplemental Fig. S8 for 2D-HPLC of the plate-grown larvae glycome. It is estimated that approximately 10% of the total N-glycomes were released with PNGase Ar. PNGase F, peptide:N-glycosidase; RP, reverse-phase.

As PNGase F does not release core α1,3-fucosylated glycans, we treated the residual glycopeptides with either native PNGase A or the more recently available recombinant PNGase Ar. In contrast to the results with PNGase F-released glycans, there was a clear difference in the chromatograms between the resulting liquid- and plate-grown L4 larval subglycomes (Figs. 5 and S6 and S7). Also, while the two preparations of liquid-grown larvae resulted in similar profiles, there was more variability between the two plate-grown samples. In terms of detected and verifiable structures, the PNGase Ar-released subglycomes were more complicated in terms of numbers of glycans (68 or 56 glycans as compared with 38 or 43 for PNGase A release; supplemental Table S1), which in part is due to the higher specific activity of the PNGase Ar enzyme but also because of its previously reported ability to release N-glycans with an α-galactosylated core α1,3-fucose residue with the signature Y_1_ ions at *m/z* 916, that is, GlcNAc_1_Fuc_2_Gal_2_-PA (26).

**Fig. 6:**
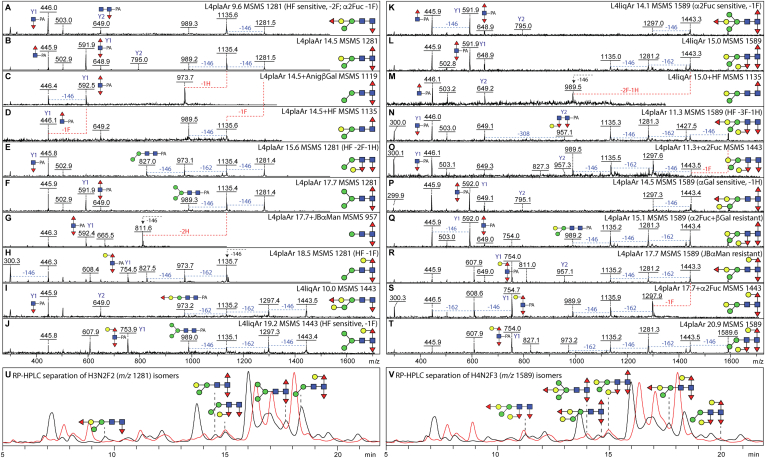
**Example of MS/MS and digestion data for PNGase Ar-released N-glycans from *Caenorhabditis elegans* L4 larvae.** RP-HPLC fractionated glycans were subject to MALDI-TOF MS and MS/MS in positive mode before and after chemical or enzymatic treatment. The MS/MS spectra are annotated with the fraction name (L4liqAr or L4plaAr and the retention time), the *m/z* value, key fragments and summarized or shown sensitivity to α- or β-galactosidase, α-mannosidase, α1,2-fucosidase, or hydrofluoric acid, resulting in the indicated losses of fucose (F), methylated fucose (FMe), or hexose (H). *A*–*J*, comparison of five isomers of *m/z* 1281 (Hex_3_HexNAc_2_Fuc_2_) and two isomers of *m/z* 1443 (Hex_4_HexNAc_2_Fuc_2_) with indications of differences in core Y1 fragments and effects of enzymatic or chemical treatments. *K*–*M*, comparison of two isomers of *m/z* 1589 (Hex_4_HexNAc_2_Fuc_3_) found in the L4 liquid glycome and the effect of HF treatment of one of them resulting in replacement of the proximal Y_1_ difucosylated fragment at *m/z* 592 with a monofucosylated one at *m/z* 446 in addition to loss of the distal GalFuc motif. *N*–*T*, comparison of five isomers of *m/z* 1589 in the L4 plate glycome and the effect of α1,2-fucosidase on two of them; the summed evidence shows variations in the positions of the fucose and galactose residues. The occurrence of difucosylated and trifucosylated chitobiose cores is in accordance with the defined activities of *C. elegans* FUT-1, FUT-6, and FUT-8 (14), the absence of such cores from the *fut-1;fut-6;fut-8* triple knockout strain (12), and others’ ESI–MS^n^ data on permethylated *C. elegans* N-glycans (21, 25). *U* and *V*, depiction of the separation of Hex_3_HexNAc_2_Fuc_2_ and Hex_4_HexNAc_2_Fuc_3_ isomers by RP-HPLC; the overlaid liquid and plate chromatograms in Figure 5 are shown in *red* and *black*, respectively. ESI, electrospray ionization; RP, reverse phase.

**Fig. 7:**
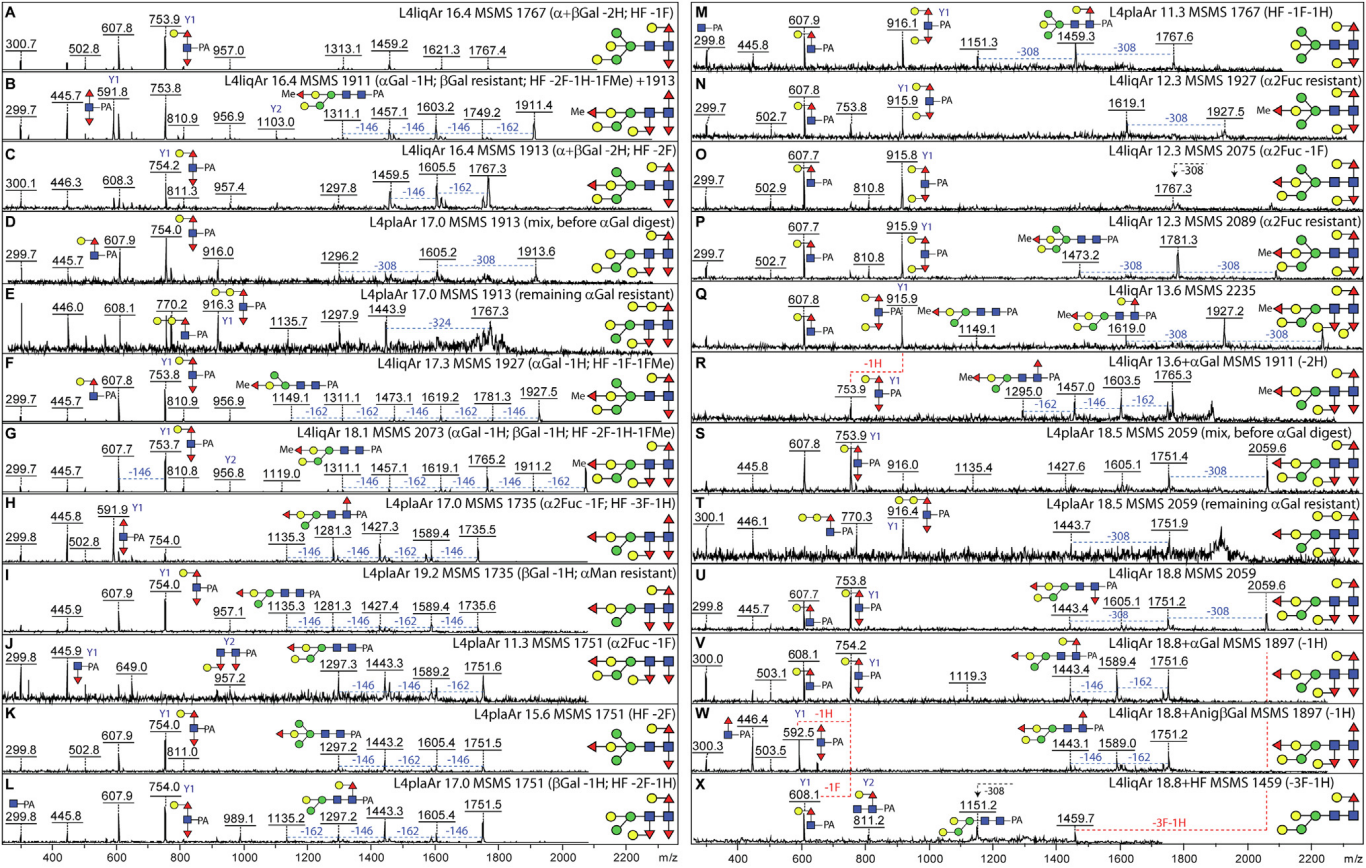
**Example of MS/MS and digestion data for PNGase Ar-released N-glycans from *Caenorhabditis elegans elegans* L4 larvae**. RP-HPLC fractionated glycans, indicated with *m/z* value, and the fraction name (L4liqAr or L4plaAr and the retention time), were subject to MALDI-TOF MS and MS/MS in positive mode before and after chemical or enzymatic treatment. The various examples of highly core modified N-glycans were defined on the basis of the MS/MS spectra and summarized or shown sensitivity to α- or β-galactosidase (loss of hexose; H), α1,2-fucosidase (loss of fucose, F, linked to bisecting galactose), or hydrofluoric acid (loss of core proximal and distal α1,3-fucose and any attached galactose residues or of fucose/methylated fucose linked to bisecting galactose). *A*, *U*–*X*, glycans of *m/z* 1767 and 2059 from the liquid glycome 16.4 and 18.8 min fractions with dominant *m/z* 754 Y_1_ fragments (Hex_1_HexNAc_1_Fuc_2_-PA) were sensitive to coffee bean α- and *Aspergillus niger* β-galactosidase and hydrofluoric acid treatments, whereby the latter glycan corresponds to the α-galactosidase-sensitive structure in the 18.5 min fraction of the L4 plate glycome (panel *S*). *B*–*E*, *S*, and *T*, coeluting glycans of *m/z* 1911, 1913, and 2059 were distinguished only after enzymatic digestion, whereby the α-galactosidase-resistant structures have Y fragments at *m/z* 770 and 916 (Hex_2_HexNAc_1_Fuc_1–2_-PA), indicative of a digalactosylated motif, as compared with the dominant *m/z* 754 of the α-galactosidase-sensitive isomers. *F*–*L*, glycans of *m/z* 1735, 1751, 1927, and 2073 show different sensitivities to α2-fucosidase, β-galactosidase, or HF treatments correlating with variations in the position or degree of substitution of the fucose residues. *M*–*R*, glycans of Hex_5–7_HexNAc_2_Fuc_2–4_Me_0–1_ with a core Y_1_ fragment at *m/z* 916 and galactosidase sensitivities (26) indicative of both proximal (*i.e.*, reducing terminal) core fucose residues being galactosylated; as there were no Y_1_ fragments with increments of 14 Da, it is concluded that the methylfucose residues only modify the bisecting galactose. For further examples of MS/MS of previously uncharacterized N-glycans found in L4 larvae, refer to supplemental Figs. S9–S11. PNGase, peptide:N-glycosidase; RP, reversed-phase.

Glycans of the same mass were detected in multiple HPLC fractions; their different MS/MS fragmentation patterns, which also change after chemical or enzymatic treatments, indicate that isomeric separation based on elution time was achieved. For instance, there are five or more forms of Hex_3–5_HexNAc_2_Fuc_2–3_ (*m/z* 1281, 1443, 1589, 1605, and 1751), whereas glycans of larger mass displayed the least variability, for example, there is only one detected isomer of Hex_7_HexNAc_2_Fuc_4_Me_1_ (*m/z* 2235). The major MS/MS fragments are either core Y-ion fragments (especially *m/z* 446, 592, 754, and 916; GlcNAc_1_Fuc_1–2_Gal_0–2_-PA) or those resulting from serial loss of fucose and galactose residues, whereby core α1,3-fucose residues are relatively labile as compared with the core α1,6- or bisecting α1,2-fucose-type motifs (Figs. 6 and 7 for selected data as well as supplemental Figs. S8–S11). In accordance with earlier studies (10, 26), hydrofluoric acid effectively removes proximal and distal core α1,3-fucose residues as well as partially the α1,2-fucose on the bisecting galactose, whereas the microbial α1,2-fucosidase only removed the latter (Figs. 6, *D*, *M*, *O* and *S*, 7*X* and S8*L*); methylated fucose was only removed by hydrofluoric acid (supplemental Figs. S8*I* and S11*G*).

In some cases, enzymatic treatments indicated that there were two isomeric structures in the same fraction, whereby α-galactosidase-resistant glycans with a digalactose modification of the α1,6-fucose and an unsubstituted α1,3-fucose were revealed (MS/MS fragments at *m/z* 770 and 916; Fig. 7, *E* and *T*), the former being a core motif found in adult worms (13, 26). On the other hand, more structures displayed α-galactosylation of the core α1,3-fucose in the liquid-grown worms as opposed to the plate-grown worms (MS/MS fragments of *m/z* 608 and 916, see Figure 7, *M*–*Q* and α-galactosidase treatment in Fig. 7*R*). Coffee bean α-galactosidase not only removed the galactose substitution of the core α1,3-fucose but also that linked to the α1,3-mannose (Figs. 7*V* and S10). In terms of β-galactosylation, it was observed that β-galactosidases from both *A. nidulans* and *A. niger* (44) remove galactose from the core α1,6-fucose (Figs. 3*Z*, 7*W*, S8*G* and S11*N*), whereas removal of the bisecting galactose is only possible by *A. niger* β-galactosidase as shown for simple structures (Figs. 6*C* and S2*H*), but modification of the distal GlcNAc appears to sterically hinder this enzyme.

Drawing also on earlier data regarding the glycan motifs in *C. elegans*, we conclude that the possible isomeric variations are in the positions of the fucose residues (core α1,3 or core α1,6 linked on the proximal GlcNAc, α1,3 linked on the distal, and α1,2 linked to the bisecting β1,4-galactose), the occurrence of α- or β-linked galactose (on either core fucose or α1,3-mannose), or the presence of α1,6-mannose residues. As for the PNGase F-released subglycomes, α-galactosylation on the α1,3-mannose and methylation of the α1,2-fucose appeared to be more abundant in the liquid-grown samples (supplemental Fig. S12).

### O-Glycans of Embryos, L4 Larvae, and Mixed Culture *C. elegans*

**Fig. 8:**
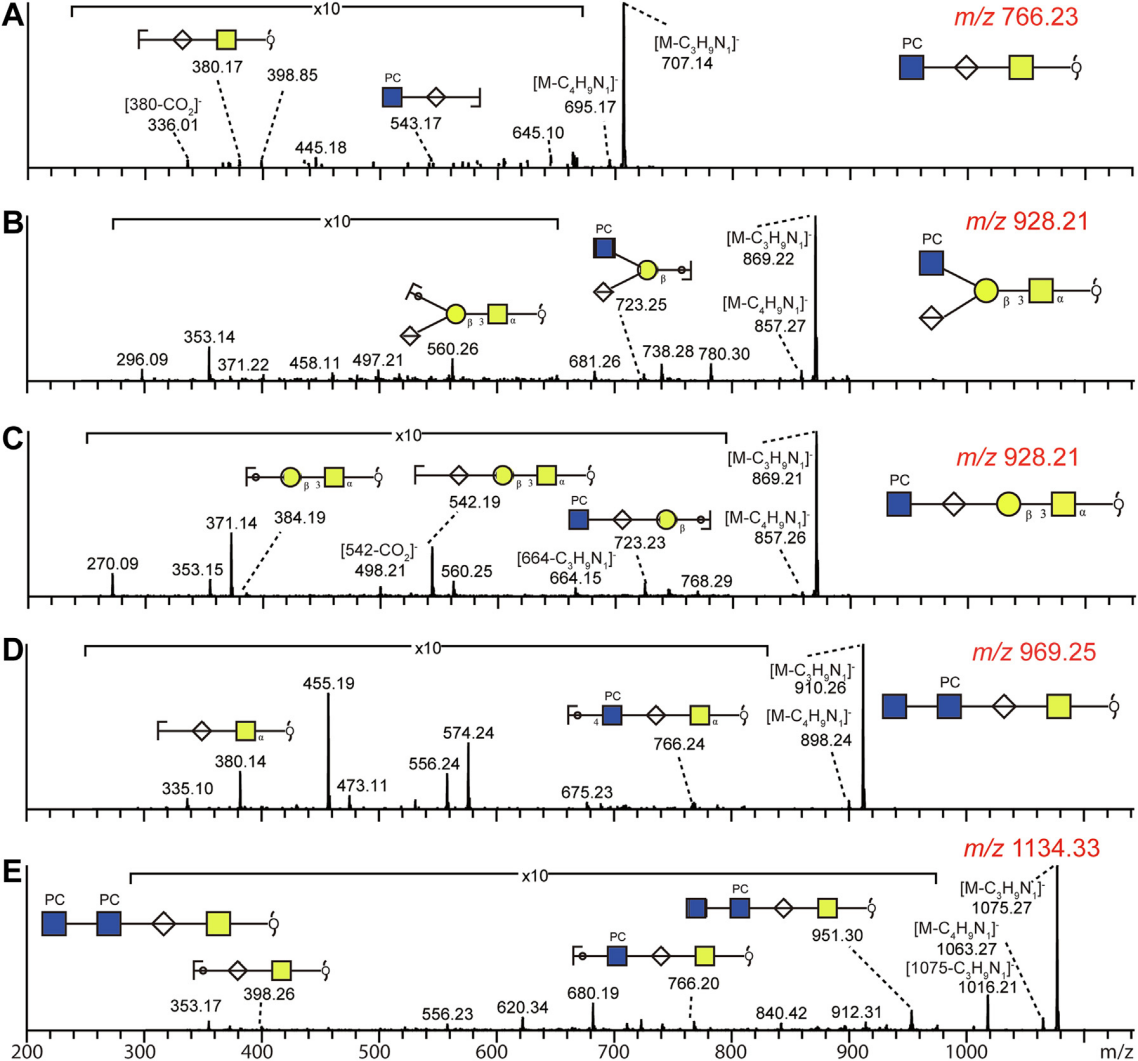
**Phosphorylcholine is a modification of O-glycans from *Caenorhabditis elegans.*** Negative-mode LC–ESI–MS/MS analyses indicate the occurrence of five O-glycan structures carrying HexNAc_1_PC_1_ motifs. The fragment ions at [M-H-59]^−^ or [M-H-N(CH_3_)_3_]^−^ are diagnostic for phosphocholine containing glycans and arise by loss of the trimethylamine group. Z fragment ions containing subterminal hexuronic acid residues are often accompanied by loss of the carboxyl group [Z-CO_2_]^−^ (*A* and *C*), a characteristic absent from MS/MS of glycans with nonreducing hexuronic acid (*B*). As for N-glycans, HexNAc_2_PC_1–2_ motifs exist as nonreducing terminal modifications of O-glycans (*D* and *E*). For MS/MS of selected neutral O-glycans, refer to supplemental Fig. S13. ESI, electrospray ionization.

Glycopeptides remaining after PNGase A treatment were subject to reductive β-elimination and LC–MS/MS analysis of the released O-glycans. The dominant structures found in embryo, L4 larvae, and adults are probably based on core 1 Galβ1,3GalNAc disaccharide with varying abundance of Hex_2_HexNAc_1_Fuc_1_ and Hex_3–4_HexNAc_1_ being the major differences (supplemental Fig. S13 and supplemental Table S2). Some of the structures are compatible to those proposed for *C. elegans* on the basis of NMR or MS data (18, 27). However, compared with studies performed on permethylated glycans (8, 18, 20), our data reveal for the first time O-glycans modified with phosphorylcholine residues, two of which (*m/z* 766 and 969) have been previously found in insects (58) and which are more pronounced in the three embryonal samples. Negative-ion mode MS/MS spectra show dominant deprotonated ions at [M-H-59]^−^ because of the diagnostic loss of the trimethylamine group from the phosphorylcholine moiety. The fragments at *m/z* 543, 723, and 766 revealed the occurrence of HexNAc_1_HexA_1_PC_1_-based motifs (Fig. 8), whereas positive-ion mode MS/MS for the linear glycan with *m/z* 930 resulted in an intense HexNAc_1_HexA_0–1_PC_1_ fragment at *m/z* 369 and 545 (data not shown).

### Transcriptomic Analysis of Genes Encoding Potential Golgi Proteins

Glycosylation of proteins is a nontemplate-driven process, and the glycome of an organism is dependent on the spatiotemporal expression of a relevant set of glycosyltransferases and remodeling glycosidases. Therefore, we extracted a subset of already-published transcriptomic data, focusing on those genes with proven biochemical function in N-glycan modification (supplemental Fig. S14); there are variations in expression with a seemingly reciprocal relationship for the expression of redundant α1,2-mannosidase, GlcNAc-TI, and Golgi hexosaminidases (59, 60, 61). Also, there is a shifted increase during embryonal development for the three proven core FUT genes (14, 15, 62), whereby the two GT10 core α1,3-FUTs (FUT-1 and FUT-6) are later expressed as compared with the GT23 core α1,6-FUT (FUT-8), whose expression is similar to that of the GT92 α1,6-fucose-modifying β1,4-galactosyltransferase 1 (GALT-1) (63). This could correlate with the relative lack of trifucosylated and tetrafucosylated glycans and the occurrence of only one glycan with a distal core α1,3-fucose residue in the embryos, whereas the “Galβ4Fucα6” motif is well represented throughout the life cycle. Similarly, expression of the GLY-2 N-acetylglucosaminyltransferase V increases first at 400 min, which may explain the lack of triantennary structures in the embryo.

As there is a general lack of information regarding most of the enzymes necessary to generate the highly structurally and developmentally variable N-glycome, we postulated that it may be possible to gain clues as to which enzymes may be involved in glycan maturation by examining the transcriptome datasets in more detail. Considering that the typical Golgi glycan-modifying enzyme possesses a single N-terminal transmembrane domain and is typically of 300 to 600 amino acids (64), we sought to identify a subset of the *C. elegans* genome encoding such proteins. The list was supplemented with further known glycosidase and glycosyltransferase genes, resulting in a set of over 700 genes. Interestingly, a number of clusters of potential glycosylation-related genes was identified, for example, C13A2.1–C13A2.12, K06H6.1–K06H6.6, or T15D6.1–T15D6.12 (supplemental Fig. S15). The genes encoded within these clusters are often putative glycosyltransferases of different CAZy GT families (65), but potential methyltransferases, NDP-sugar transporters, as well as proteins with domains of unknown function (DUF268, DUF273, and DUF1647) are also represented; without biochemical data, it is unknown whether this clustering is indicative of consecutive functions in glycan metabolism in an operon-like manner as in bacteria, considering also that multiple members of the same gene family occur in some of these clusters.

**Fig. 9:**
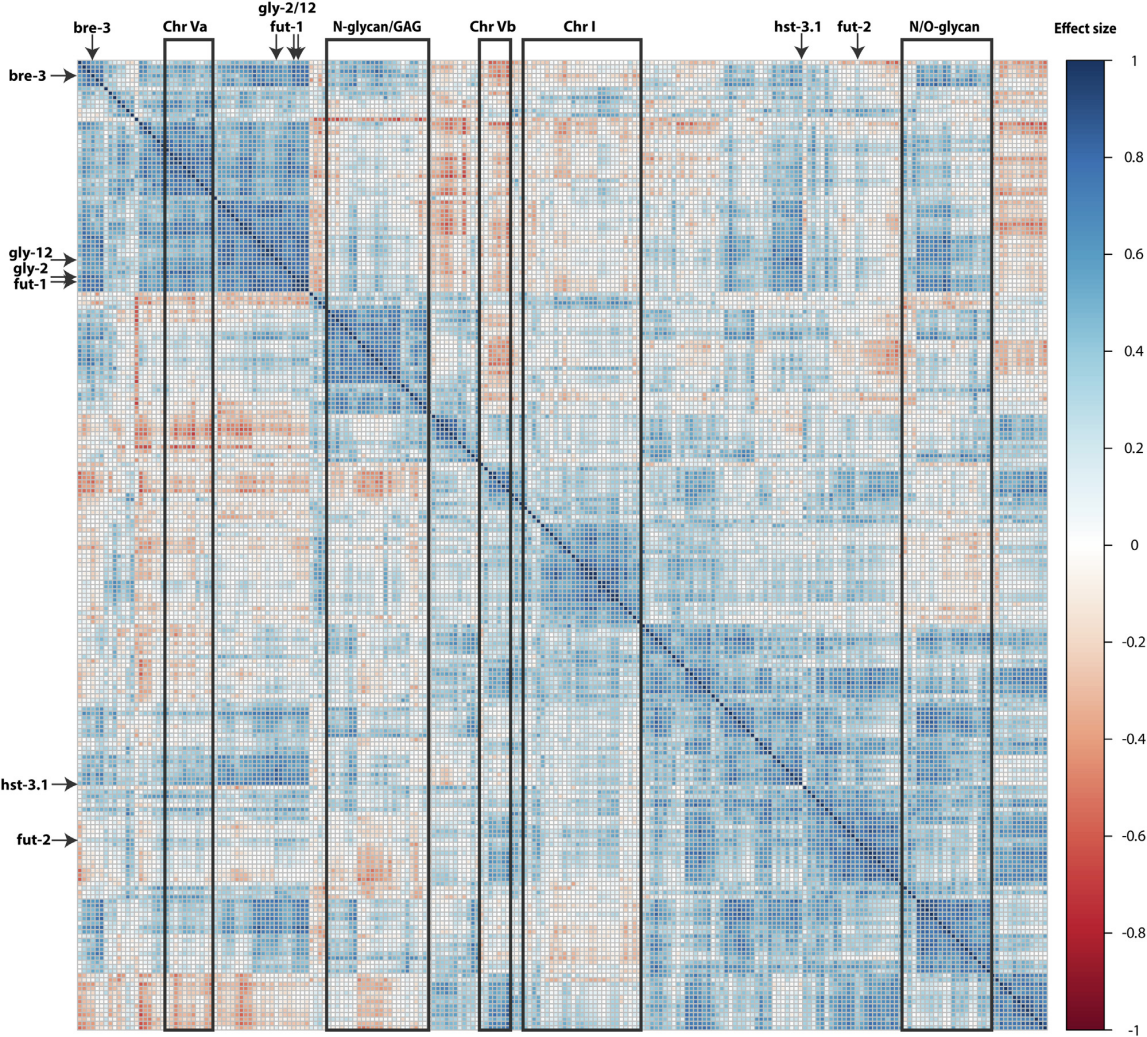
**Cluster analysis of 285 genes with known or potential roles in glycosylation.** Corrplot cluster analysis of RNA-Seq transcriptomic data (L1–L4, dauer, and adult) for 285 genes encoding proteins of either known roles in glycosylation and/or present in potential glycogene clusters and/or member of CAZy families GT2, GT7, GT10, GT11, GT13, GT14, GT16, GT18, GT23, GT27, GT43, GT47, GT49, GT92, GH20, GH38, or GH47. Correlations in expression as indicated by intensity of the effect size (*blue*/*red*; *i.e.*, high or low correlation) are highlighted for three potential glycogene clusters on chromosome I and V (supplemental Fig. S15) as well as genes with known functions in N-/O-glycan or glycosaminoglycan biosynthesis, including various *fut* (*fucosyltransferase*), *gly* (*glycosylation*), *hex* (*hexosaminidase*), *mans* (*class I mannosidase*), and *sqv* (*squashed vulva*) genes. A higher resolution form of the figure annotated showing all gene names is shown in supplemental Fig. S16*B*.

We then generated extended heatmaps for (i) all 700 genes potentially encoding single transmembrane domain Golgi proteins and (ii) a subset of 285 genes either with proven biochemical function in glycosylation and/or present in the “glycoclusters” and/or displaying homology to other glycosyltransferases. An initial perusal of the transcriptomic clustering of the set of 700 genes (supplemental Fig. S16*A*) suggested similar temporal expression of some genes with known roles in N-glycan biosynthesis as well as some genes in potential glycoclusters. The more targeted analysis of the subset of 285 genes (Figs. 9 and S16*B*) also showed such trends with, for example, one GlcNAc-TI gene (*gly-14*), the single GlcNAc-TII gene (*gly-20*), and two class I and one class II mannosidase genes (*mans-1*, *mans-4*, and *aman-2*) displaying similar temporal expression in the embryonal and postembryonal stages as was the case for some genes in the C13A2/F07G11, K06H6/ZK488, and T09E11/T15D6/E03H4 genomic regions. For the larval and adult stages, some of the genes required for glycosaminoglycan biosynthesis clustered in terms of expression, for example, *sqv-3*, *-5*, *-8*, and *rib-2*, as also another set of proven N-glycosylation genes (*fut-8*, *gly-13*, *hex-2*, *mans-2*, and *mans-3*). Thus, there is potentially coordinated expression of genes with either a functional or a spatial relationship.

## Discussion

**Fig. 10:**
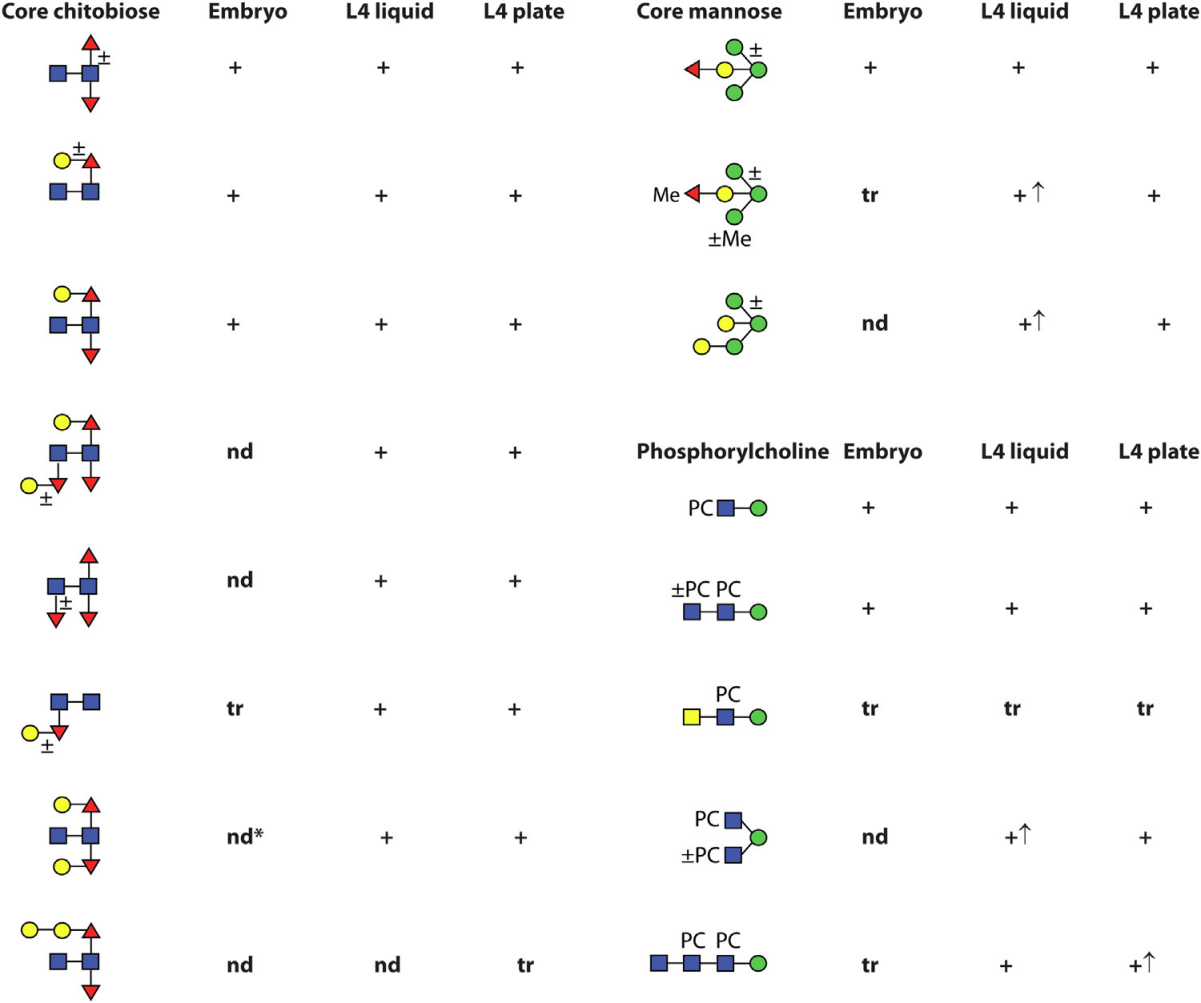
**Summary of core and antennal motifs of *Caenorhabditis elegans* N-glycans and trends in their expression.** Based on the cumulative evidence (Fig. 1, Fig. 2, Fig. 3, Fig. 4, Fig. 5, Fig. 6, Fig. 7 and Supplementary Data), various N-glycan structural motifs can be proposed, which have been detected in the different samples. nd, not detected; tr, trace; ↑, increased occurrence; ∗, N-glycans with the di-GalFuc-core (Y_1_ fragment of *m/z* 916) can only be released by PNGase Ar. PNGase, peptide:N-glycosidase.

It has become widely presumed that glycosylation changes during development. Examples include glycomic shifts during the schistosome life cycle (4), in development of the porcine parasite *Oesophagostomum dentatum* (3) or the sheep parasite *Haemonchus contortus* (38), in frog morphogenesis (66), different parts of the mammalian brain (67) or the degree of IgG galactosylation as compared with age (68). Previous studies on *C. elegans* N-glycans have also made this conclusion (7). Here, we show a large increase in N-glycomic complexity between embryos and L4 larvae using an off-line LC-MALDI-TOF-MS approach. Thereby, we could also detect isomeric structures as well as ones of low abundance. Also, cultivation on plates or in liquid culture alters the N-glycome. Overall, over 200 different structures were identified with confidence in the different embryo and L4 samples (supplemental Table S1), which display a variety of core and antennal modifications (Fig. 10).

In terms of the N-glycome of wildtype embryos, 25 glycan masses are found both in the current study and that of Geyer *et al.* (57) based on mass spectrometric screening; this number decreases to 13 when considering the *glp-1* embryos. Of the 40 masses we detect, partly in multiple fractions, in wildtype embryos, only two were not detected in the *glp-1* glycome. Thus, different studies in different laboratories can come to different conclusions, which may be due to the exact glycomic workflows. Certainly, the use of HPLC fractionation enables us to determine isomers, as retention times, MS/MS, and chemical/enzymatic treatments can distinguish various structural motifs; thus, we gained more structural information than some previous studies. When considering distinct isomers, 10 structures were not detected in *glp-1*, overlapping strongly with the nine absent from *aqx-1*; most of these “missing glycans” were core α1,3-fucosylated (supplemental Table S1). This is either a coincidence or indeed an indication that defective Notch signaling impacts a minor subset of the *C. elegans* N-glycome, although some differences in relative O-glycan occurrence may occur between the strains (supplemental Table S2).

As compared with the embryo, the L4 larval N-glycomes are more complicated in terms of numbers of structures, which could be annotated (over 100 per sample rather than 60) and the actual structural complexity. Here, the two-step release procedure with PNGase F followed by different forms of PNGase A enabled us to better investigate the core α1,3-fucosylated glycans by avoiding coelution with more abundant structures. In addition to the typical range of oligomannosidic and paucimannosidic structures, the PNGase F-released glycomes show the presence of numerous low abundance phosphorylcholine-modified glycans reminiscent of those found in the parasitic *O. dentatum*, partly based on chito-oligomers (3), and only rarely on LacdiNAc (Fig. 2, Fig. 3, Fig. 4). Such complex structures with up to three antennae have previously not been found in the *C. elegans* glycome; however, unlike some distantly related parasitic nematodes (69, 70, 71), neither tetra-antennary nor anionic N-glycans were found. On the other hand, it was the PNGase A/PNGase Ar-released subglycomes, which showed the most obvious differences in the RP-HPLC chromatograms between liquid- and plate-grown nematodes, whereby the PNGase Ar also released N-glycans with “double” GalFuc-substituted reducing termini (Figs. 5 and S6); this is in accordance to our published data on this enzyme (26), but here a number of previously undetected structural variants have been found, including many with bisecting β-linked galactose, a modification defined by us *via* ESI–MS and NMR as a feature unique to *C. elegans* (12); difucosylation and trifucosylation of the chitobiose unit and galactosylation of core fucose residues, on the other hand, are features known from other clade V nematodes including *H. contortus* and *O. dentatus* (3, 38). In contrast, the differences in the mucin-type O-glycome are in terms of percentage occurrence rather than the presence or the absence of certain structures, whereby the phosphorylcholine-modified forms are also novel (supplemental Table S2). The newly detected N- and O-linked zwitterionic glycans in *C. elegans* add to the repertoire of such phosphodiester modifications of nematode N-glycans (41, 72), glycolipids (73, 74) and glycosaminoglycans (75).

A glycomic variation dependent on cultivation method is not entirely unexpected as morphological and transcriptomic differences between liquid (either with bacteria or axenic) and plate-grown *C. elegans* have been previously observed (76, 77), although a direct effect on glycosylation or glycosyltransferases was not noted. However, not unexpectedly, the swimming-type behavior of *C. elegans* in liquid culture does cause oxidative stress (78), and so the increased occurrence of methylated and α-galactosylated N-glycans we observe in both samples of liquid-grown L4 larvae may be direct or indirect markers of stress in this organism. Comparing the PNGase F-released glycomes (as shown by two independent preparations), there are nine or 10 detected α-galactosylated glycans in the liquid-cultivated worms and four or three from the plate, overlapping in part with the eight or seven methylated structures in the liquid grown or four or five in the plate grown; this trend is also evident for the PNGase A- and PNGase Ar-released glycomes. Overall, it is estimated that there are eightfold increases in the abundance of α-galactosylated and methylated N-glycans in the liquid-grown larvae as compared with those cultivated on plates (supplemental Fig. S12). This suggests a differential expression of the relevant mannose-modifying α-GALT and fucose-modifying methyltransferase. However, such enzymes remain to be identified.

Looking for a direct relationship between transcriptome and glycome, in order to explain developmental alterations in the ensemble of glycans, is only partly possible from the available data. While differences in core FUT expression may relate to observed shifts in core chitobiose modifications (*fut-1* and *fut-6* having a delayed increase in expression as compared with *fut-8*; supplemental Fig. S14), the challenge with *C. elegans* is that the enzymology underlying the overall N-glycome is still understudied. For instance, we do neither know the nature of most of the β-GALTs nor of the α-GALTs, methyltransferases, α1,2-FUTs, or phosphorylcholinyltransferases, which modify N-glycans of this organism; considering the comparative glycomic information on nematodes, it is expected that some of these enzymes, such as the bisecting β-GALT, will be unique to *C. elegans* as compared with parasitic nematode species (24). In the case of mucin-type O-glycans, the structures are not all identified in terms of monosaccharides or their linkages, rather often only in terms of mass or fragmentation, and perhaps only three of the enzymologically characterized glycosyltransferases of *C. elegans* (core 1 GALT, the GLY-1 β1,6-glucosyltransferase, and the CE2FT-2 α1,2-FUT) have a substrate specificity indicative of a role in O-glycan synthesis (79, 80, 81); this leaves O-glycan-modifying glucuronyltransferases, glucosyltransferases, and phosphorylcholinyltransferases still to be identified. Comparisons to other nematodes require further O-glycomic studies.

Nevertheless, compiling a list of potential Golgi enzymes on the basis of their predicted length and topology is an initial step toward identifying candidate genes for further analysis, and the available data suggest potential for coordinated expression of putative glycosylation gene clusters (Figs. 9, S15 and S16). However, unlike the cases in bacteria where a single polycistronic mRNA can contain multiple glycosyltransferase reading frames (82), in *C. elegans*, an operon pre-mRNA can be cis- and trans-spliced to result in a number of mature monocistronic mRNA molecules with either SL1 or SL2 5′-spliced leaders, which are then translated individually (83, 84). The existence of polycistronic mRNAs can explain the similar expression profiles for the three glycogene clusters on chromosomes I and V, whereas the stage-correlated transcription of N- and O-glycosylation-relevant genes outside these clusters could be dependent on transcription factors. Indeed, perusal of modENCODE chromatin immunoprecipitation-sequencing data (85) indicates that there are binding sites for the DAF-16 and PHA-4 transcription factors at or near the 5′-ends of, for example, the *aman-2*, *gly-9*, *gly-13*, *gly-20*, *mans-1*, *mans-2*, and *sqv-6* genes, whereas NHR-77 (rather than DAF-16) may bind the promoters of, for example, *galt-1*, *gly-5*, *gly-6*, *gly-10*, *hex-2*, or *mans-3*; intriguingly, the *nhr-77* gene lies within the glycogene cluster on chromosome I. The long-term aim of predicting the glycome from the genome requires the biochemical function of the glycosyltransferases and other glycan-modifying enzymes encoded by the glycogene clusters to be defined as well as more targeted analysis of expression and transcriptional control under different growth conditions.

In conclusion, we show that there is an increase in glycomic complexity between the embryonal and larval L4 stages of *C. elegans* and also distinct differences dependent on larval cultivation method. We define not only just N-glycan structures with a diverse variety of core modifications but also longer phosphorylcholine-modified antennae, found in even greater abundance in another study on adult worms using a modified glycomic workflow (86). Thus, despite 20 years of glycomic research on *C. elegans*, we continue to discover new structures in this model nematode and can wonder as to the functional repercussions of its possessing such as diverse glycome distinct from those of related species.

## Data Availability

Data described in the article are shown in the figures; mzxml files have been submitted to Glycopost: https://glycopost.glycosmos.org/entry/GPST000294.

## Supplemental data

This article contains supplemental data including further information regarding the glycomic analyses (Supplementary Figures S1-S16 and Supplementary Tables S1-S2) (11, 12, 13, 14, 18, 26, 27, 41, 65, 80, 87, 88, 89).

## Conflict of interest

The authors declare no competing interests.

## Supplemental Data

Supplemental data

SuppFigure16A_transcriptome

SuppFigure16B_corrplot_adult
