# Supplementary material for: Increasing Complexity of the N-Glycome During Caenorhabditis Development
Source: Mol Cell Proteomics. 2023 Jan 28;22(3):100505. doi: 10.1016/j.mcpro.2023.100505 (PMC7614267; doi:10.1016/j.mcpro.2023.100505)
Supplement: Supplemental data [file mmc1.pdf]

Increasing complexity of the N-glycome during *Caenorhabditis* development

Iain B. H. Wilson, Shi Yan (闫石), Chunsheng Jin (金春生), Zuzanna Dutkiewicz, Dubravko Rendić, Dieter Palmberger, Ralf Schnabel and Katharina Paschinger

Supplementary Information

Further information regarding the glycomic analyses

Definition of the level of the glycan structural analysis:

The goal was the N-glycomic analysis of (i) *Caenorhabditis elegans* N2 embryos as compared to *apx-1* and *glp-1* embryos and (ii) and *Caenorhabditis elegans* L4 larvae grown either in liquid culture or on plates. Thereby, individual glycan-containing HPLC fractions were subject to MALDI-TOF MS and MS/MS, in combination with a range of chemical and enzymatic treatments. The embryo samples were prepared once, while for L4 larvae, cultivation/preparation was performed twice. In addition, O-glycans from embryo, L4 and adult samples were analysed by LC-ESI-MS/MS.

Search parameters and acceptance criteria

a. **Peak lists:** As stated in the methods section: typically 1000 shots were summed for MALDI-TOF MS and 5000 for MS/MS. Spectra were processed with the manufacturer's software (Bruker Flexanalysis 3.3.80) using the SNAP algorithm with a signal/noise threshold of 6 for MS (unsmoothed) and 3 for MS/MS (four-times smoothed).
b. **Search engine, database and fixed modifications:** All glycan data were manually interpreted and no search engine or database was employed; the fixed modification is the pyridylamine label at the reducing end ($GlcNAc_1$-PA fragments of *m/z* 300).
c. **Exclusion of known contaminants and threshold:** All glycan data were manually interpreted; only peaks with an MS/MS consistent with a pyridylaminated chitobiose core were included – the 'threshold' for inclusion was an interpretable MS/MS spectrum (at least in terms of composition).
d. **Enzyme specificity:** A description of the release methods (either with PNGase A alone or PNGase F followed by PNGase A or Ar) is given in the methods section. Enzymes used during the analysis (glycosyl hydrolases) are defined in the methods by species name and supplier. Citations for in-house purified recombinant enzymes are also given in the methods section. As previous experience with normalizing glycosidase amounts based on units of activity towards *p*-nitrophenyl sugars reduced digestion efficiency towards native oligosaccharides, aliquots of glycans (equivalent to 5 – 50 mV in terms of fluorescence) were incubated overnight with 0.2 µl of the various enzyme preparations (whether commercial or in-house produced, except for α and β-galactosidases which were desalted and/or further diluted before used due to the presence of MS-detectable non-glycan contaminants). These conditions result in no obvious unspecific removal of residues as defined by shifts in mass, MS/MS or retention times. Ammonium acetate buffers were used as suppliers' buffers interfere with MALDI-TOF MS analysis; generally one-quarter of any glycosidase digest was applied directly to the target plate prior to drying and addition of matrix.

   Fucosidases: A microbial α1,2-fucosidase (recombinant; Megazyme) was used in this study to remove non-methylated fucose residues attached to the bisecting galactose. No evidence for removal of core α1,3- or α1,6-fucose by this enzyme was observed as the Y_1 fragments were unaltered.
   Galactosidases: Coffee bean α-galactosidase (native; Sigma) will remove the galactose residue

attached to the α1,3-mannose of the di/tri-mannosylchitobiosyl core as well as the galactose attached to the proximal core α1,3-fucose, but its action may be inhibited by steric hindrance; *Aspergillus nidulans* β-galactosidase (His-tag purified recombinant form expressed in *Pichia pastoris*) will remove the galactose attached to the core α1,6-fucose; *Aspergillus niger* β-galactosidase (His-tag purified recombinant form expressed in *Pichia pastoris*) will remove the galactose attached to the core α1,6-fucose (completely) and the 'bisecting' galactose residue attached to the core β1,4-mannose (about 50% overnight).

Hexosaminidases: Jack bean β-*N*-acetylhexosaminidase (native; Sigma) is a general-purpose enzyme unspecifically removing β-linked GlcNAc residues; *Streptomyces plicatus* β1,3/4-*N*-acetylhexosaminidase ('chitinase' recombinantly-expressed in *E. coli*; NEB) removes β1,4-linked GalNAc from LacdiNAc and β1,4-linked GlcNAc from chitobiose motifs, but no other GlcNAc residues; *C. elegans* HEX-4 is an in-house prepared enzyme (His-tag purified recombinant form expressed in *Pichia pastoris*) which demonstrably removes β1,4-linked GalNAc residues, but in this and previous studies is not observed to remove any other HexNAc residue.

Hydroflouric acid: Treatment (3 µl of 48% HF added to the dried glycan) was 48 hours on ice in the cold room prior to drying under vacuum; expected release of α1,3-fucose and phosphodiester residues, but not of other sugars, was observed under these conditions. Partial release of α1,2-fucose by HF was corroborated by α1,2-fucosidase digestions, except for methylated fucose residues.

Mannosidases: Jack bean α-mannosidase (native or recombinant; respectively, Sigma or NEB) removes all α-mannose residues, but steric hindrance slows its action, e.g., digestion of core α1,6-mannose is minimal under the employed conditions if there is a 'lower' arm modification on the core α1,3-mannose or bisecting galactose prevents removal of the α1,3- and α1,6-mannose residues; a more specific α1,2/3-mannosidase (*Xanthomonas*; NEB) was also used for treatment of two embryonal glycan fractions.

Phosphorylcholine esterase: *Streptococcus pneumoniae* PCE (recombinant; kind gift of Dr. Nicolas Gisch, Research Center Borstel) can remove phosphorylcholine residues from teichoic acids and was applied in the current study to selected pools of PC-modified N-glycans from L4 larvae, albeit the digests required solid phase extraction before mass spectrometry and release of PC was incomplete, as compared to HF treatment.

e. **Isobaric/isomeric assignments:** For isomeric species, RP- or 2D-HPLC elution, differences in MS/MS and/or digestion data were used for the assignment (as described in the text).

Glycan or glycoconjugate identification

a. **Precursor charge and mass/charge (*m/z*):** All glycans detected were singly-charged. For the positive mode, the *m/z* values are for protonated forms. Depending on the glycan amount, preparation or presence of buffers in exoglycosidase preparations, the relative amounts of the H^+, Na^+ and K^+ adducts varied. Maximally two decimal places used for the *m/z* annotations consistent with the accuracy of MALDI-TOF MS; in the figures and due to space limitations, only one decimal place is presented. Previous data indicate an average +0.03 Da (+ 22 ppm) deviation between the measured and the calculated *m/z* values on the instrument used.
b. **MALDI-TOF MS settings (positive mode):** Ion Source 1 and 2 were 19.00 and 16.75 kV; Lens, 9.00 kV; Reflector 1 and 2, 21.05 and 9.65 kV; Pulsed Ion Extraction, 160 ns; Matrix Suppression typically up to 700 Da; Detector Gain, typically 2163 V.
c. **MALDI-TOF MS/MS settings (positive mode):** Ion Source 1 and 2 were 6.00 and 5.35 kV; Lens, 2.90 kV; Reflector 1 and 2, 27.00 and 11.75 kV; Lift 1 and 2, 19.00 and 4.00 kV; Pulsed Ion Extraction, 140 ns; Detector Gain, typically 2260 V when fragmenting; Laser Power Boost typically 50%; not in CID mode; PCIS typically 0.65%.

d. **All assignments:** For the glycans present in each pool, see the RP-HPLC chromatograms annotated with structures shown according to the Standard Nomenclature for Glycans. Downwardly- and upwardly-drawn core fucose and mannose residues are respectively α1,3- and α1,6-linked (see **Figures 1, 2 and 5** in the main text) .

e. **Modifications observed:** Listed are the *m/z* values for glycans carrying a reducing terminal pyridylamine group as judged by presence of an *m/z* 300 $GlcNAc_1$-PA fragment. As the glycans are otherwise chemically unmodified, *Δm/z* of 146, 160, 162, 165 and 203 correspond to deoxyhexose (presumed to be fucose), methylated fucose, hexose, phosphorylcholine or *N*-acetylhexosamine. As glycans from *C. elegans* are not detected in the negative mode, there is no indication for the presence of sialic acid, phosphate, phosphoethanolamine, sulphate or glucuronic acid residues.

f. **Number of assigned masses:** Glycan assignments were not just based on measured mass only, but on the basis of MS/MS corroborated by digest and elution data.

g. **Spectra:** Representative annotated spectra (MS and MS/MS) defining structural elements are given in various figures. In total, MS and/or MS/MS data for some 70 of the approximately 200 defined structures or digestion products thereof are shown. The overall data is based on nearly 1400 MS and 3000 MS/MS spectra; over 660 mzxml files for the MS/MS data are available.

h. **Structural assignments:** As noted in the results section, the typical oligomannosidic structures are assigned based on elution time and fragmentation pattern; it is otherwise assumed that the glycans contain a trimannosyl core consistent with typical eukaryotic N-glycan biosynthesis up to the processing by GlcNAc-TII (MGAT2). The presence of a GlcNAc-TV (MGAT5) homologue, but no GlcNAc-TIV (MGAT4), in *C. elegans* is compatible with the proposed tri-antennary glycans, whose fragmentation patterns show preferential loss of the 'heaviest' antenna. However, due to the low abundance of some glycans or occasional steric hindrance, ambiguity remained regarding which modifications were on the three antennae.
   The assignments of antennal and core fucose residues are based on RP-HPLC retention time, fragmentation pattern and/or susceptibility to digestions; galactose modifications of *C. elegans* N-glycans have been previously corroborated by ESI-MS^n, NMR and/or GC-MS. Other antennal modifications (phosphorylcholine and chito-oligomers) are defined based on digestions and fragmentation patterns after digestion, whereby these are known from other nematodes. There is no evidence of in-source fragmentation of the N-glycans.

Summary of glycosidases used in this study

PNGases	*Flavobacterium* PNGase F (recombinant, expressed in *E. coli*)<br>Almond PNGase A (native almond)<br>Almond PNGase A (recombinant almond, expressed in Hi5 insect cells)<br>Rice PNGase Ar (recombinant, expressed in *P. pastoris*)
Fucosidases	Microbial α1,2-fucosidase (recombinant)
Galactosidases	Coffee bean α-galactosidase (native, desalted)<br>*A. niger* and *A. nidulans* β-galactosidases (recombinant, *P. pastoris*)
Hexosaminidases	Jack bean β-*N*-acetylhexosaminidase (native)<br>*S. plicatus* β1,3/4-*N*-acetylhexosaminidase (recombinant, *E. coli*)<br>*C. elegans* HEX-4 β1,4-*N*-acetylgalactosaminidase (recombinant, *P. pastoris*)
Mannosidases	Jack bean α-mannosidase (native)<br>*Xanthomonas* α1,2/3-mannosidase (recombinant, *E. coli*)
PC-esterase	*S. pneumoniae* PCE phosphorylcholine esterase (recombinant, *E. coli*)

Supplementary Table S1: Summary of N-glycan structures. Retention times in minutes are shown for the eleven different preparations: embryos (N2, t3208 and e2144; green), L4 liquid (F, first in red; F, second in black; A, first in red; Ar, second) and L4 plate (F, first in red; F, second; A, first in red; Ar, second). nd, not detected; tr, trace. The theoretical protonated monoisotopic *m/z* for the pyridylaminated N-glycans, the summarized composition, the retention time in terms of glucose units (in light blue) and, as appropriate, the relevant figures are also indicated. Structures are based on MS/MS as well as results of chemical/enzymatic treatments (for examples see the figures and mzxml files available via Glycopost) and comparisons to our previous studies on nematode N-glycans. For ESI-MS^n and NMR data on bisected glycans, refer to Yan *et al*., 2015a, 2015b and 2018 (*J. Prot. Res.* 14, 5291–5305, *Mol. Cell. Proteomics* 14, 2111-2125 and *Anal. Chem.* 90, 928-935); for GC-MS analysis of the *m/z* 1646 N-glycan with one proximal and one distal GalFuc modification, see Yan *et al.*, 2012 (*J. Biol. Chem.* 287, 28276–28290). GlyTouCan accessions (in dark blue; e.g., G67083FT) are included for selected simpler glycans. Percentage occurrence of glycans from the second L4 liquid and plate preparations was calculated on the basis of integrated fluorescence of RP-HPLC fractions divided by MALDI-TOF-MS peak areas.

Structures	*m/z*, [M+H]$^+$;<br>Composition;<br>g.u.; Figure	Emb<br>N2 A	Emb<br>*apx-1* A<br>(t3208)	Emb<br>*glp-1* A<br>(e2144)	L4<br>Liquid F<br>(F 1st)	L4<br>Plate F<br>(F 1st)	L4<br>Liquid Ar<br>(A 1st)	L4<br>Plate Ar<br>(A 1st)
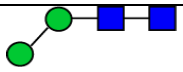	827.34<br>H2N2<br>5.9	14.7′	14.7′	14.5′	tr 14.4′<br>>0.1%	14.9′<br>>0.1%<br>13.7-14.7′		
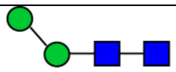	827.34<br>H2N2<br>7.8	tr 17.4′	nd 17.3′	tr 17.2′	tr 17.2′-18.4<br>0.3%<br>15.8-17.6′	17.1-18.2′<br>0.1%<br>16.5-17.6′	18.1′ Ar	
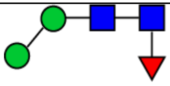	973.40<br>H2N2F1<br>4.2	tr 11′	nd 10.7′	nd 10.8′			10.6′ Ar<br>>0.1%<br>9.2′ A	10.7′ Ar<br>>0.1%<br>9.8′ A
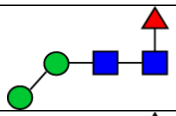	973.40<br>H2N2F1<br>10	tr 20.8′	nd 20.7′	tr 20.8′	tr 20.6′<br>>0.1%<br>tr 19.5′	tr 20.6′<br>>0.1%<br>tr 19.5-20.5′		
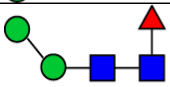	973.40<br>H2N2F1<br>15-16	22.4-23.5′	23.2′	22.2-23′	tr 22.1′ F<br>0.2%<br>tr 22.5′	tr 22.1′<br>0.3%<br>22.5′	23.4′ Ar	
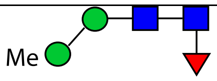	987.41<br>H2N2F1Me1<br>4.7-5.1						tr 12.3′<br>>0.1%<br>11′ A	tr 12.1′<br>>0.1%<br>11.2′ A
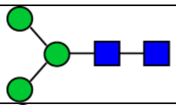	989.39<br>H3N2 (MM)<br>7.2-7.6	17-17.4′	17.2′	17.2′	17.2′<br>14.3%<br>15.8-16.5′	17.1′<br>13.5%<br>16.5′	17.3′ Ar<br><br>16.2′ A	17′ Ar<br><br>16′ A
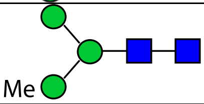	1003.41<br>H3N2Me<br>9.5				19.2′ F<br>>0.1%<br>nd 18.2′	tr 18.2′<br>>0.1%<br>18.5′ F		
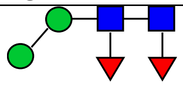	1119.46<br>H2N2F2<br>5.8						13.6-14.1′ Ar<br>>0.1%<br>tr 12.2′ A	13.7′ Ar<br>>0.1%<br>13′ A
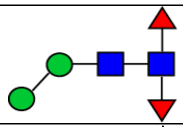	1119.46<br>H2N2F2<br>7-7.6<br>G67083FT						17.3′ Ar<br>>0.1%<br>tr 16.2′ A	17′ Ar<br>>0.1%<br>16′ A
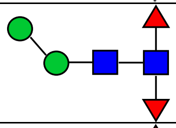	1119.46<br>H2N2F2<br>8-8.8<br>G05890XZ						17.8′ A	
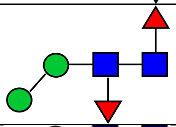	1119.46<br>H2N2F2<br>> 13-16				23.8′<br>>0.1%<br>tr 22.5′	23.8′<br>0.1%<br>22.5- 23.5′		
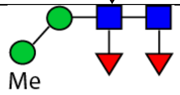	1133.47<br>6-6.3<br>Supp. Fig. 11A						15′ Ar<br>>0.1%<br>14′ A	15.1′ Ar<br>>0.1%<br>14′ A

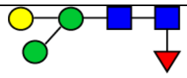	1135.45<br>H3N2F1<br>2.8						7.8′ Ar<br>>0.1%	7.8′ Ar<br>>0.1%<br>7′ A
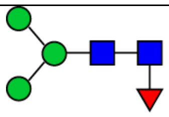	1135.45<br>H3N2F1<br>(MMF³)<br>4.9<br>G92612ZM	tr 12.5′	tr 12.4′	nd 12.5′			12.3′ Ar<br>0.1%<br>11′ A	12.1′ Ar<br>0.2%<br>11.2′ A
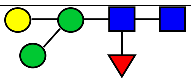	1135.45<br>H3N2F1<br>5.2					13.1′<br>>0.1%		
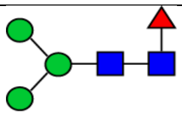	1135.45<br>H3N2F1<br>(MMF⁶)<br>11.5<br>G45995IV	22′	22.5′	22.2′	22.2′<br>7.4%<br>21.5-22.5′	22.1′<br>7.3%<br>21.5-22.5′	22.2-23.4′ Ar<br><br>22′ A	22.3′ Ar
Me<br>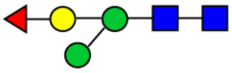	1149.47<br>H3N2F1Me1<br>5.2<br>Yan 2015a				13′ F<br>>0.1%<br>tr 11.8′	13.1 F<br>>0.1%<br>11.8′	13.1′ Ar	12.9′ Ar
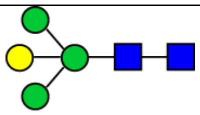	1151.45<br>H4N2<br>4.2<br>Yan 2015b	11′	10.7′	10.8′	10.8′ F<br>1.3%<br>9.5′	10.7′ F<br>2.7%<br>9.5-10′		10.7′ Ar<br>9.8′ A
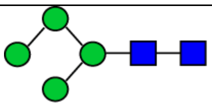	1151.45<br>H4N2<br>7.8	17.4′	17.3′ tr	17.2′	17.2′ F<br>0.5%	18.2′<br>0.7%<br>17.6′		
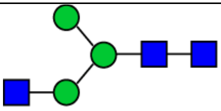	1192.47<br>H3N3 (MGn)<br>7.2<br>G70073SG	16.1-17′	16-16.8′	16-16.8′	16.6′<br>2.6%<br>15.8′	16.7′<br>2.9%<br>15.8′	16.4′ Ar	
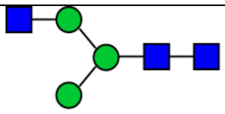	1192.47<br>H3N3 (GnM)<br>10.6-12<br>G06920GM	20.8-21.5′	21.5′	20.6-21.3′	21.5′<br>0.9%<br>20.5-21.2′	20.6-21.6′<br>1.0%<br>20.5′	22.2′ Ar	
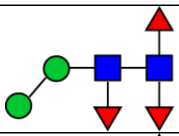	1265.51<br>H2N2F3<br>7.2-8.8						18.8′ Ar<br>>0.1%	18.5′ Ar<br>0.2%<br>17.8′ A
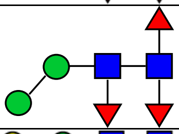<br>Me	1279.53<br>H2N2F3Me1<br>10<br>Supp. Fig. 11B						19.9′ Ar<br>>0.1%	tr 19.6′ Ar<br>>0.1%
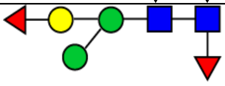	1281.51<br>H3N2F2<br>3.8<br>Fig. 6A						10′ Ar<br>>0.1%	9.6′ Ar<br>0.2%<br>8.7′ A
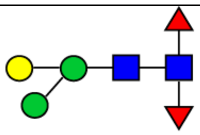	1281.51<br>H3N2F2<br>5.8-6.4<br>Fig. 6B						15′ Ar<br>>0.1%	14.5-15.1′ Ar<br>>0.1%<br>14′ A
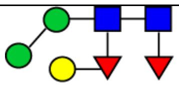	1281.51<br>H3N2F2<br>6.2-6.8<br>Fig. 6E	15.3-16′	nd 15.2′	15.2′			15.8′ Ar<br>>0.1%	15.6′ Ar<br>>0.1%
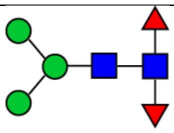	1281.51<br>H3N2F2<br>(MMF³F⁶)<br>7.5-8.2<br>Fig. 6F<br>G77479VH	tr 17.4′	nd 17.3′	nd 17.2′			tr 17.3′ Ar<br>>0.1%<br>tr 17′ A	17-17.7′ Ar<br>0.3%<br>17′ A
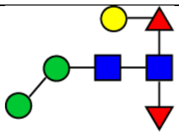	1281.51<br>H3N2F2<br>8.8<br>Fig. 6H						tr 18.8-19.2′<br>>0.1%	18.5-19.6′ Ar<br>>0.1%<br>tr 17.8′

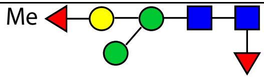	1295.52<br>H3N2F2Me1<br>3.8						10′ Ar<br>>0.1%	9.6′ Ar<br>>0.1%
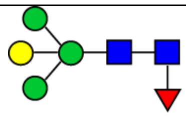	1297.50<br>H4N2F1<br>2.6<br>Supp. Fig. S2B	7.2′	7.2′	7.4′			7.3′ Ar<br>>0.1%<br>6.5′ A	7.3-7.8′ Ar<br>0.5%<br>6.5-7′ A
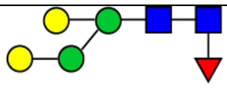	1297.50<br>H4N2F1<br>3						8.3′ Ar<br>>0.1%	8.2′ Ar<br>>0.1%<br>7′ A
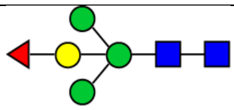	1297.50<br>H4N2F1<br>4.2<br>Fig. 3P<br>Yan 2015b	11′	tr 10.7′	tr 10.8′	10.2-10.8′<br>0.7%<br>9.3′	10.6-10.7′<br>1.5%<br>9.5′	10.6′ Ar<br>9.2′ A	10.7′ Ar<br>9.8′ A
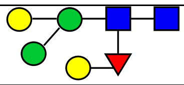	1297.50<br>H4N2F1<br>5.2					tr 12.3-13.2′		
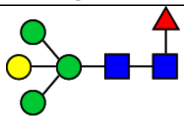	1297.50<br>H4N2F1<br>6.7	16.1′	16′	16′	15.7′<br>>0.1%<br>14.7′	15.7′<br>0.3%<br>14.7′		
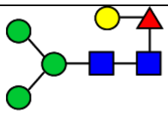	1297.50<br>H4N2F1<br>15-16<br>Supp. Fig. 2L	25′	24.5′	24.4′	24.3′<br>0.1%<br>24.7′	24.3′<br>0.3%<br>23.5′		
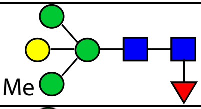	1311.52<br>H4N2F1Me1<br>3.3	7.9′	tr 8.2′	nd 8.4′				
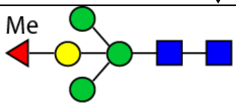	1311.52<br>H4N2F1Me1<br>4.8<br>Fig. 3A<br>Yan 2015b				12.1′<br>0.4%<br>tr 10.8′	12.0′<br>0.4%<br>tr 10.8′	12.3′ Ar	tr 12.1′ Ar
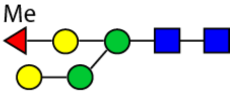	1311.52<br>H4N2F1Me1<br>5.5<br>Fig. 3B<br>Yan 2015b				13.6′<br>0.1%			
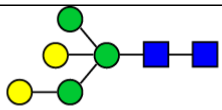	1313.50<br>H5N2<br>4.6				11.3′<br>1.4%<br>10.0′	11.0′<br>0.4%<br>10.0′		
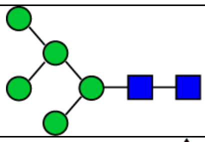	1313.50<br>H5N2<br>(Man5)<br>7.1	17′	16.8′	16.8′	16.6′ F<br>4.3%<br>15.8′	16.7′ F<br>5.1%<br>15.8′		
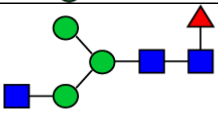	1338.53<br>H3N3F1<br>(MGnF^{6})<br>10.2-12<br>G14576KZ	20.8-21.5′	21.5′	20.6′	21.5′<br>1.6%<br>21.2′	20.6-21.5′<br>1.6%<br>20.5′	22.2′ Ar	20.6-21.7′ Ar
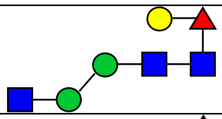	1338.53<br>H3N3F1<br>12-14				tr 23.8′<br>>0.1%	tr 23.8′	23.4′ Ar	
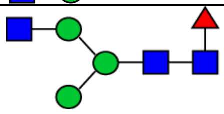	1338.53<br>H3N3F1<br>(GnMF^{6})<br>17<br>G69987TD	26′	26′	25-25.8′	25.8′<br>0.6%<br>26′	25.8′<br>0.5%<br>23.5′-26′		
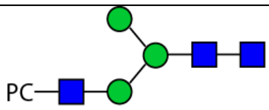	1357.53<br>H3N3PC1<br>7.8	17.4′-18.5′	17-18.3′	17.2-19′	17.2-18.4′<br>5.2%<br>16.5′	17.1-18.2′<br>2.6%<br>17.6′	18.1-18.8′ Ar	
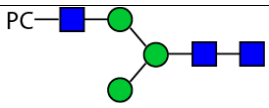	1357.53<br>H3N3PC1<br>12-14<br>Supp. Fig. S2I	22.4′	22.5′	22.2′	22.2′ F<br>0.3%	22.1′ F<br>0.3%	23.4′ Ar	

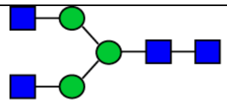	1395.55<br>H3N4 (GnGn)<br>9-10<br>G88876JQ	19.9′	19.8′	19.7′	19.8′<br>1.2%<br>19-19.5′	19.7′<br>2.0%<br>19.5′	19.9′ Ar	
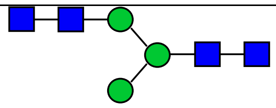	1395.55<br>H3N4<br>>11-15	23.5′	23.2′	nd 23′	nd 24.3′	23.8′<br>>0.1%		
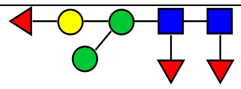	1427<br>H3N2F3<br>4.8<br>Supp. Fig. 9A							11.3-11.6′ Ar<br>>0.1%
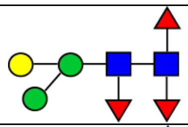	1427.56<br>H3N2F3<br>6-6.4<br>Supp. Fig. 9B							15.1-15.6′ Ar<br>14′ A<br>>0.1%
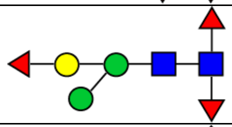	1427.56<br>H3N2F3<br>6.5-7.4						tr 16.4′<br>>0.1%	16-17′ Ar<br>>0.1%
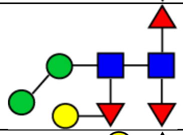	1427.56<br>H3N2F3<br>8-9						19.2′ Ar<br>>0.1%	18.5-19.2′ Ar<br>0.1%<br>17.8′ A
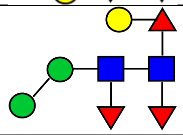	1427.56<br>H3N2F3<br>10<br>Supp. Fig. 9C							20.6′ Ar<br>0.1%
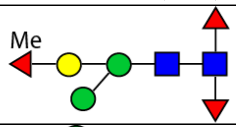	1441.58<br>H3N2F3Me1<br>6.3-7.2<br>Supp. Fig. 11C						16.4′ Ar<br>>0.1%	
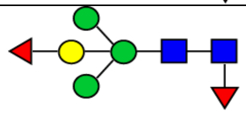	1443.56<br>H4N2F2<br>2.6	7.2′	7.2′	7.4′			7′ Ar<br>0.1%<br>6′ A	7.3′ Ar<br>0.5%<br>6′ A
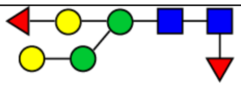	1443.56<br>H4N2F2<br>3.8<br>Fig. 6I						10′ Ar<br>>0.1%<br>8.5′ A	
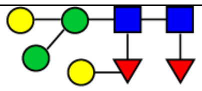	1443.56<br>H4N2F2<br>4<br>Supp. Fig. 9D							9.6′ Ar<br>>0.1%<br>8.7′ A
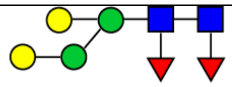	1443.56<br>H4N2F2<br>4.5<br>Supp. Fig. 9E						10.6′ Ar<br>>0.1%<br>tr 9.2′	
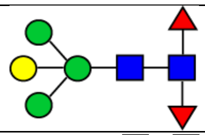	1443.56<br>H4N2F2<br>5.2-5.5<br>Supp. Fig. S2E	13.4′	13.3′	13.8′			13.6-14.1′ Ar<br>>0.1%<br>13′ A	13.7′ Ar<br>0.5%<br>13-13.4′ A
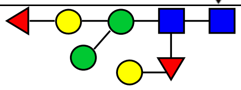	1443.56<br>H4N2F2<br>5.5<br>Fig. 3U					13.6′<br>0.1%<br>12.3′		
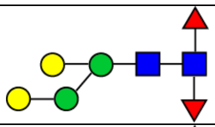	1443.56<br>H4N2F2<br>5.8-6.4<br>Supp. Fig. 9F						15′ Ar<br>>0.1%	
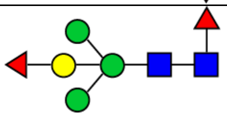	1443.56<br>H4N2F2<br>6.7<br>Fig. 3X				tr 14.7′	14.9-15.7′<br>0.3%<br>14.7′		15.7′ Ar

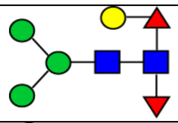	1443.56<br>H4N2F2<br>8.8-9<br>Fig. 6J	19.9’	nd 19.8’	nd 19.7’			19.2-19.9’ Ar<br>>0.1%<br>18.5’ A	19.2-19.6’ Ar<br>0.4%<br>19.4’ A
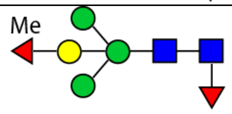	1457.58<br>H4N2F2Me1<br>3.0	7.9’	tr 8.2’	tr 8.4’			8.3’ Ar<br>0.2%<br>7.2’ A	8.2’ Ar<br>0.1%<br>7.5’ A
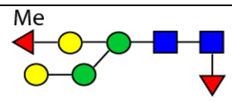	1457.58<br>H4N2F2Me1<br>4.1						10.6’ Ar<br>>0.1%<br>8.5-9.2’ A	
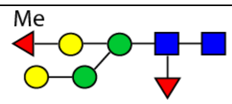	1457.58<br>H4N2F2Me1<br>6.2				15.2’<br>>0.1%<br>14-14.7’			
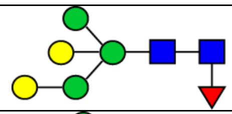	1459.56<br>H5N2F1<br>2.8	7.9’	nd 8.2’	nd 8.4’			7.8’ Ar<br>0.4%<br>6.5’ A	7.8’ Ar<br>>0.1%<br>7’ A
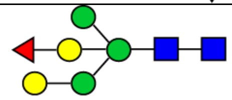	1459.56<br>H5N2F1<br>4.6<br>Fig. 3D	tr 11’	tr 10.7’	nd 10.8’	tr 10.8’<br>0.3%<br>10’	tr 10.7’<br>>0.1%<br>10’	10’ A	
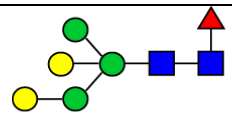	1459.56<br>H5N2F1<br>6.7				15.8’			
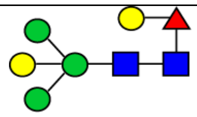	1459.56<br>H5N2F1<br>8.4-9<br>Fig. 3Y<br>G98795XC	18.5’	18.3’	18.2’	18.4’<br>0.1%<br>18.2’	18.2’<br>0.4%<br>18.5’		
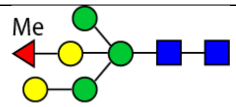	1473.57<br>H5N2F1Me1<br>5.2				13.0’<br>1.5%<br>12’	13.1’<br>>0.1%<br>11.8’	12.3-13.1’ Ar<br>tr 12.2’ A	
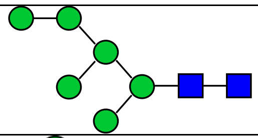	1475.55<br>H6N2<br>5.6	14’	13.9’	13.8’	tr 13.6’<br>>0.1%	13.1’<br>>0.1%		13.7’ Ar
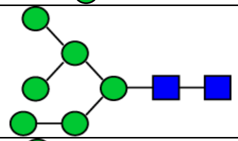	1475.55<br>H6N2<br>5.9	14.7’	14.7-15.2’	14.5’	14.4’<br>5.1%<br>13.2’	14.3-14.9’<br>6.1%<br>13.2’	15’ Ar<br>13’ A	14.5’ Ar
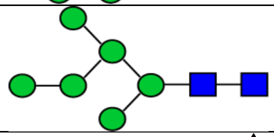	1475.55<br>H6N2<br>7.8-8.5	18.5’	18.2’	18.2’	18.4’<br>0.2%<br>tr 17.6’	18.2’ F<br>0.6%<br>17.6’		
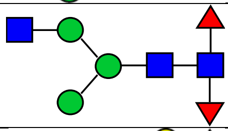	1484<br>H3N3F2<br>9-11<br>G28654UV						19.9’ Ar<br>>0.1%<br>20’ A	tr 19.6’ Ar<br>>0.1%<br>20’ A
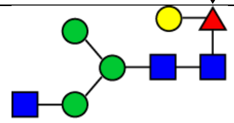	1500.58<br>H4N3F1<br>14-16		23.2’		23.8’<br>0.2%<br>tr 24.7’	23.8’-24.3<br>0.3%<br>23.5’	24.5’ Ar	
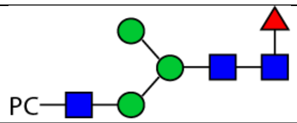	1503.59<br>H3N3F1PC1<br>12.6-16	23.5’	22.5’	23’	22.2’<br>0.9%<br>22.5’	22.1’<br>0.5%<br>21.5-22.5’	23.4-24.5’ Ar	
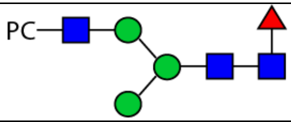	1503.59<br>H3N3F1PC1<br>>17	27’	nd 27.8’	nd 27.7’	25.8-27.1’<br>0.2%<br>tr 26’	27.2’<br>0.2%<br>26-28’		

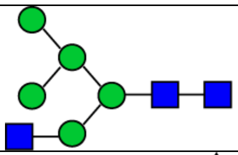	1516.58<br>H5N3<br>7.1	16.1′	16′	16′	15.7′<br>>0.1%<br>tr 15.8′	15.7-16.7′<br>>0.1%<br>15.8′		
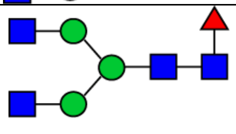	1541.61<br>H3N4F1<br>($GnGnF^6$)<br>15-16<br>Fig. 3L/M	25′	24.8′	24.4′	24.3′<br>1.5%<br>23.5-24.7′	23.8-24.3′<br>1.3%<br>23.5-25′	24.5′ Ar	24.3′ Ar
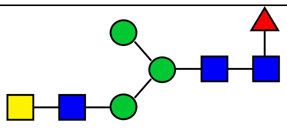	1541.61<br>H3N4F1<br>15-16<br>Fig. 3L/M				24.3′ F (mix)<br>0.2%<br>25′	tr 25′ F<br>0.2%<br>23.5-25′		
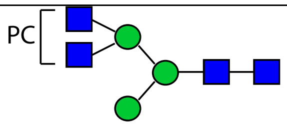	1560.61<br>H3N4PC1<br>6.3<br>Fig. 4G				15.2′<br>>0.1%<br>14′ F	14.7′		
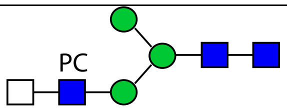	1560.61<br>H3N4PC1<br>10.5	20.8′	20.7′	20.7′	19.5′			
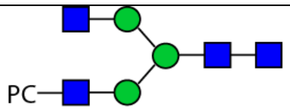	1560.61<br>H3N4PC1<br>10.2-11	21.5-22.4′	21.5′	21.5-22.2′	19.8-20.6′<br>1.9%<br>19.5-20.6′	20.6′<br>1.0%<br>19.5′/20.6′	21.2′	
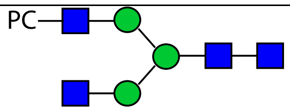	1560.61<br>H3N4PC1				21.5′<br>0.1%<br>21.2-22.5′			
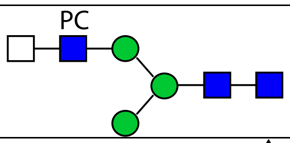	1560.61<br>H3N4PC1<br>15	25′	23.2-25′	25′	tr 23.8-24.3′<br>>0.1%<br>22.5′			
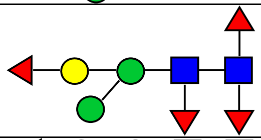	1573.62<br>H3N2F4<br>7.4<br>Supp. Fig. 9G							17′ Ar<br>>0.1%
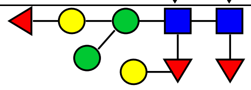	1589.62<br>H4N2F3<br>4.2<br>Fig. 6N							10.7-11.3′ Ar<br>0.1%<br>10.2′ A
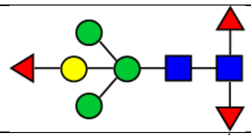	1589.62<br>H4N2F3<br>5.2-5.9<br>Fig. 6K & S8C	tr 13.4′	nd 13.3′	nd 13.3′			tr 13.6′ Ar<br>>0.1%<br>tr 13′ A	13.7′ Ar<br>0.6%<br>13′ A
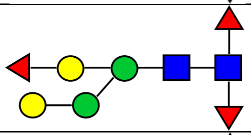	1589.62<br>H4N2F3<br>6-6.2<br>Fig. 6P						14.1′ Ar<br>>0.1%	14.5′ Ar<br>>0.1%
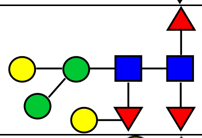	1589.62<br>H4N2F3<br>6.4<br>Fig. 6L/Q						15′ Ar<br>>0.1%	14.5-15.1′ Ar<br>0.3%<br>14.0′ A
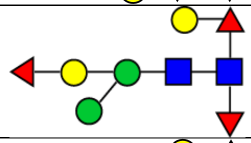	1589.62<br>H4N2F3<br>8.2<br>Fig. 6R + S8J							17-17.7′ Ar<br>0.3%<br>16-17′ A
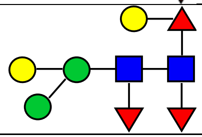	1589.62<br>H4N2F3<br>9<br>Supp. Fig. 9H							18.5′ Ar<br>>0.1%<br>17.8′ A

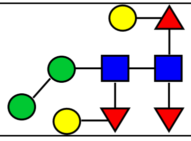	1589.62<br>H4N2F3<br>10.8<br>Fig. 6T						21.2′<br>>0.1%	20.6-20.9′ Ar<br>0.4%<br>20.0′ A
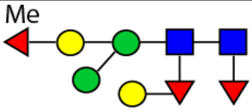	1603.64<br>H4N2F3Me<br>4.2-4.5<br>Supp. Fig. 11D						10.6′ Ar<br>>0.1%	10.7′ Ar<br>>0.1%<br>tr 9.8′ A
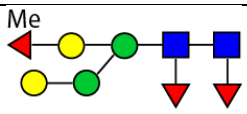	1603.64<br>H4N2F3Me<br>5<br>Supp. Fig. 11E						12.3′ Ar<br>0.1%<br>11′ A	
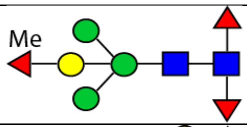	1603.64<br>H4N2F3Me<br>5.5						15′ Ar<br>0.1%<br>tr 14′	14.5′ Ar<br>>0.1%<br>13.4′ A
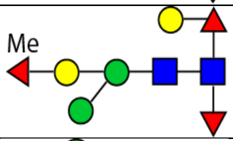	1603.64<br>H4N2F3Me<br>7.3<br>Supp. Fig. 11F						18.1 Ar<br>>0.1%	
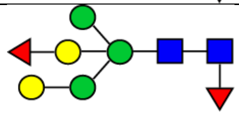	1605.61<br>H5N2F2<br>2.8						7.3-7.8′ Ar<br>0.1%<br>6.5′ A	7.3-7.8′ Ar<br>>0.1%<br>tr 6.5′ A
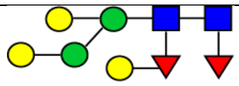	1605.61<br>H5N2F2<br>4<br>Supp. Fig. 9I						10′ Ar<br>>0.1%<br>8.5′ A	
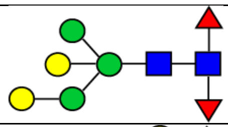	1605.61<br>H5N2F2<br>5.8-6<br>Supp. Fig. 9J		13.9′				14.1′ Ar<br>0.2%<br>13-14′ A	
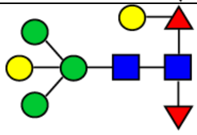	1605.61<br>H5N2F2<br>6.5-7<br>Supp. Fig. 2G<br>Supp. Fig. S8E	15.3-16.1′	15.3-16′	15.3-16′			16.4′ Ar<br>0.2%<br>tr 15.5′ A	16′ Ar<br>0.8%<br>15.5-16′ A
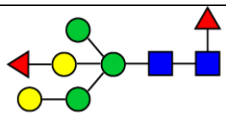	1605.61<br>H5N2F2<br>6.7				15.8′			
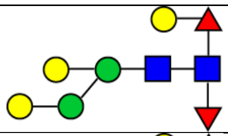	1605.61<br>H5N2F2<br>6.8-7.2<br>Supp. Fig. 9K						tr 17.3′ Ar<br>>0.1%<br>tr 16.2′ A	
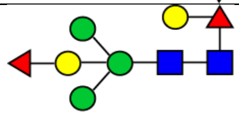	1605.61<br>H5N2F2<br>7.7				nd 18.2<br><br>17.6′	18.2′<br>0.2%<br>17.6′	tr 18.5′ Ar	tr 18.5′ Ar
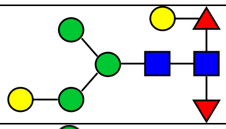	1605.61<br>H5N2F2<br>9-10						tr 19.9′ Ar<br>>0.1%	
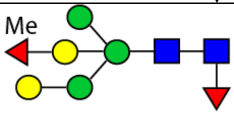	1619.63<br>H5N2F2Me1<br>3.4						8.9′ Ar<br>0.6%<br>7.2-7.8′ A	
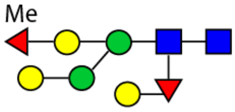	1619.63<br>H5N2F2Me1<br>6<br>Fig. 3F				14.4′<br>0.5%<br>13.2′			
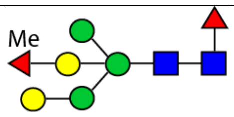	1619.63<br>H5N2F2Me1<br>7.3				16.5′			

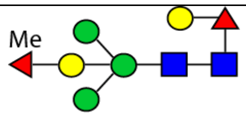	1619.63<br>H5N2F2Me1<br>9					tr 18.5′			
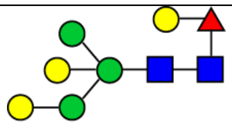	1621.61<br>H6N2F1<br>8.4-9.8<br>Fig. 3H				18.4-19.2′<br>>0.1%<br>19′	tr 18.2-19.7′<br>>0.1%<br>tr 18.5′			
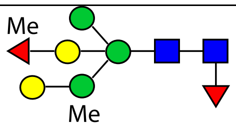	1633.65<br>H5N2F2Me2<br>5.5<br>Supp. Fig. 11I						13.6′ Ar<br>>0.1%<br>tr 12.2′ A		
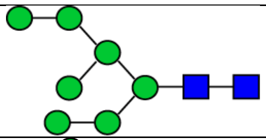	1637.60<br>H7N2<br>4.9	12.5′	12.4′	12.5′	12.1′<br>3.5%<br>10.8′	12.0′<br>2.9%<br>10.8′			
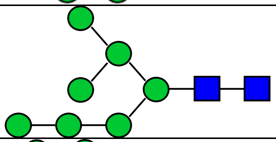	1637.60<br>H7N2<br>5.6<br>Fig. 3T	13.4-14′	13.3-13.9′	13.3-13.8′	13.6′<br>0.6%<br>12′	13.6′<br>0.6%<br>12.3′			
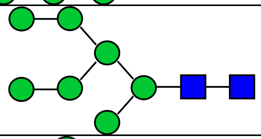	1637.60<br>H7N2<br>6.6	15.3′	15.2′	15.2′	14′				
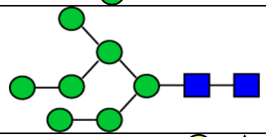	1637.60<br>H7N2<br>7	16.1′	16′	16′	15.2′<br>>0.1%<br>14-14.7′	14.9-15.7′<br>0.5%<br>14.7′			
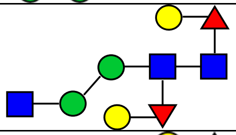	1646.64<br>H4N3F2<br>> 17<br>Yan 2012				tr 24.9′<br>>0.1%	25′<br>>0.1%	25.6′ Ar		
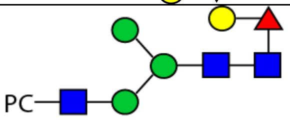	1665.64<br>H4N3F1PC1<br>16.5<br>Fig. 4A	tr 25′			24.9′<br>>0.1%<br>25/26′	25′<br>>0.1%<br>26′			
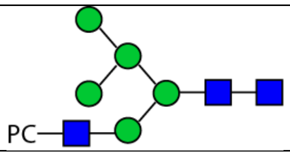	1681.63<br>H5N3PC1<br>7.3	17.4′	17.3′	17.3′	17.2′<br>0.2%	17.1′<br>0.1%			
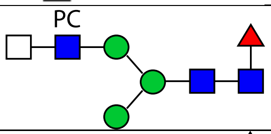	1706.67<br>H3N4F1PC1<br>>17				25′	25′			
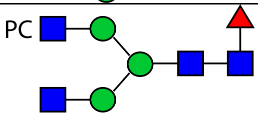	1706.67<br>H3N4F1PC1<br>> 17<br>Fig. 4K	26′	26′	25.8′	24.9-25.8′<br>1.5%<br>25-26′	25-25.8′<br>1.3%<br>25-26′			
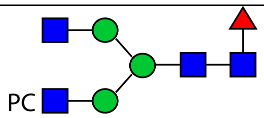	1706.67<br>H3N4F1PC1<br>16.5			27.7′	28′	28′			
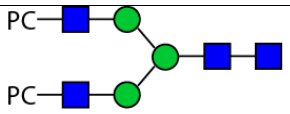	1725.66<br>H3N4PC2<br>13-18<br>Fig. 4E & S5	25-27′	25′	25′	23.8′<br>0.4%<br>22.5-25′	23.8-24.3′<br>0.5%<br>22.5′-26′	25.6′ Ar	24.3′ Ar	
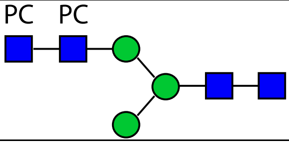	1725.66<br>H3N4PC2<br>>16	tr 27.7′	tr 27.8′	tr 27.7′	27.2-30.5′<br>0.2%<br>26′	27.2-31.2′<br>>0.1%<br>26′			

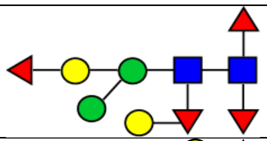	1735.68<br>H4N2F4<br>6-7.6<br>Fig. 7H<br>Supp. Fig. S8D							17′ Ar<br>0.5%<br>16′ A
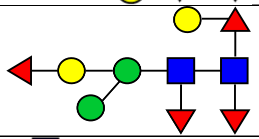	1735.68<br>H4N2F4<br>8-9<br>Fig. 7I<br>Supp. Fig. 8K							18.5-19.2′ Ar<br>>0.1%
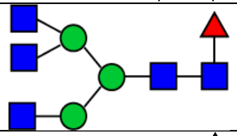	1744.69<br>H3N5F1<br>9.4-10.5				20.6′<br>0.2%<br>tr 20.5′	20.6′<br>0.2%<br>20.5′		
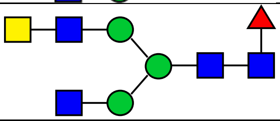	1744.69<br>H3N5F1<br>20					27.2′<br>0.2%		
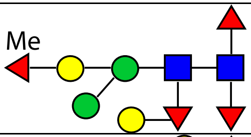 	1749.69<br>H4N2F4Me1<br>6.8						14.6′ A	
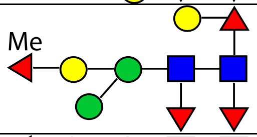 	1749.69<br>H4N2F4Me1<br>9<br>Supp. Fig. 11J						18.8' Ar<br>>0.1%	
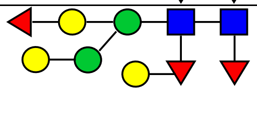	1751.67<br>H5N2F3<br>4.6<br>Fig. 7J						tr 10-11′ A	11.3′-11.6′ Ar<br>>0.1%<br>10.2′ A
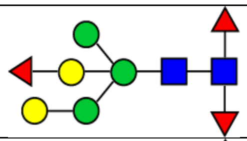	1751.67<br>H5N2F3<br>5.3-5.9<br>Supp. Fig. 9L						14.1′ Ar<br>>0.1%<br>13′ A	
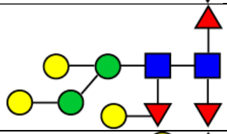	1751.67<br>H5N2F3<br>5.8-6.4<br>Supp. Fig. 9M						15′ Ar<br>0.1%<br>14′ A	
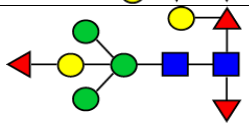	1751.67<br>H5N2F3<br>7-7.6<br>Fig. 7K<br>Supp. Fig. S8F	15.3-16.1′	16′	15.2′			tr 15.5′ A	15.6-16′ Ar<br>1.3%<br>15.5′ A
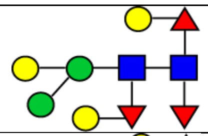	1751.67<br>H5N2F3<br>7.5<br>Fig. 7L							17′ Ar<br>0.5%<br>16′ A
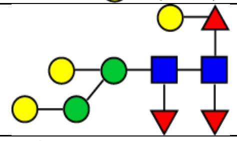	1751.67<br>H5N2F3<br>7.5-8.2<br>Supp. Fig. 9N						18.1′ Ar<br>>0.1%<br>17′ A	
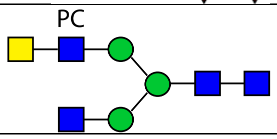 	1763.69<br>H3N5PC1<br>12-14<br>Supp. Fig. S2J	23.5-25′	tr 22.5′	22.5-23′	22.2′<br>0.5%<br>22.5′	22.1′<br>0.4%<br>22.5′		
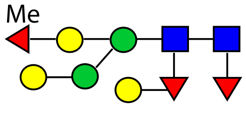 	1765.69<br>H5N2F3Me1<br>4.2<br>Supp. Fig. 10A						10.6-11.3′ Ar<br>0.5%<br>9.2-10′ A	
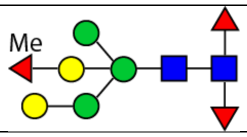 	1765.69<br>H5N2F3Me1<br>6.4<br>Supp. Fig. 10C						15′ Ar<br>0.2%<br>14′ A	

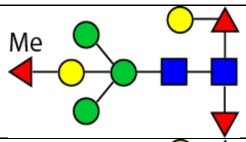	1765.69<br>H5N2F3Me1<br>6.5-7.5	tr 17′	tr 16.8′	tr 16.8′			16.4′ Ar<br>>0.1%<br>tr 15.5′ A	17′ Ar<br>0.1%<br>16′ A
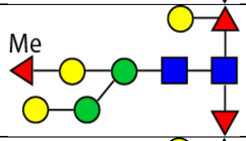	1765.69<br>H5N2F3Me1<br>8.2<br>Supp. Fig. 10E						18.1′ Ar<br>0.2%<br>17′ A	
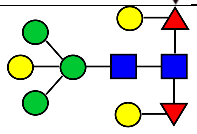	1767.67<br>H6N2F2<br>4.6<br>Fig. 7M<br>Yan 2018							11.3′ Ar<br>>0.1%
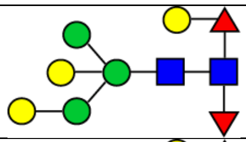	1767.67<br>H6N2F2<br>6.5-7.5<br>Fig. 7A						16.4′ Ar<br>0.7%<br>15.5-16.2′ A	tr. 16′ Ar<br>>0.1%<br>16′
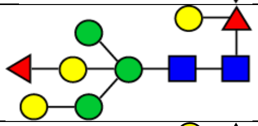	1767.67<br>H6N2F2<br>8.4-9.2				18.4′<br>0.2%<br>17.6′	18.2′<br>>0.1%<br>tr 17.6′		
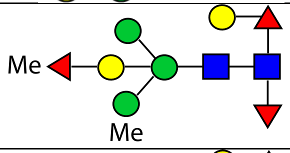	1779.70<br>H5N2F3Me2<br>7.5<br>Supp. Fig. 11K						17.3′ Ar<br>0.1%	
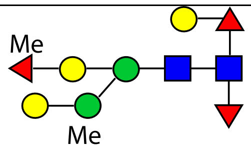	1779.70<br>H5N2F3Me2<br>9.0						19.2′ Ar<br>>0.1%	
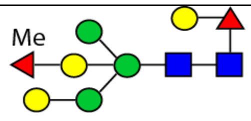	1781.68<br>H6N2F2Me1<br>9.4<br>Fig. 3I				19.8′ F<br>0.5%<br>19.5′ F			
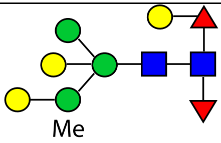	1781.68<br>H6N2F2Me1<br>9-11<br>Supp. Fig. 11L						19.2-19.9′ Ar<br>>0.1%	
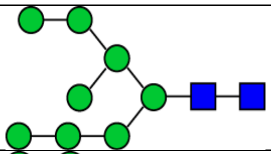	1799.66<br>H8N2<br>(Man8B)<br>4.6<br>Supp. Fig. S2C	11.7′	11.5′	11.6′	tr 11.3′<br>10′	11.3′<br>10′	tr 11.3′ Ar	11.3′ Ar
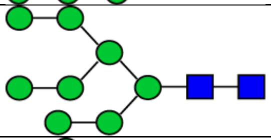	1799.66<br>H8N2<br>(Man8A)<br>5.2<br>Supp. Fig. S2D	13.4′	13.3′	13.3′	13′<br>4.4%<br>12.3′	13.1′<br>5.2%<br>11.8-12.3′		
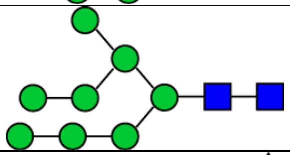	1799.66<br>H8N2<br>(Man8C)<br>6.5	14.7-15.3′	14.5′-16′	14.5-16′	14.4-15.7′<br>0.6%<br>14.7′	15.7′<br>0.1%<br>14.7′		
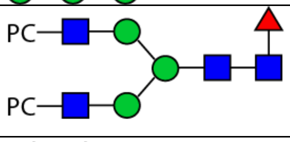	1871.72<br>H3N4F1PC2<br>>17<br>Fig. 4B<br>Supp. Fig. S5				27.1-28.9′<br>0.3%<br>32.5′	28.9-29.3′<br>0.2%<br>32.5′		
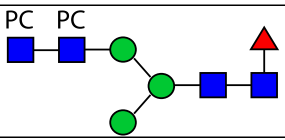	1871.72<br>H3N4F1PC2<br>>17<br>Fig. 4C				30.5′<br>>0.1%	30.5-31.2′<br>>0.1%		

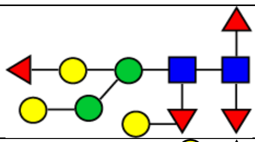	1897.73<br>H5N2F4<br>6.2-7.5						16.2′ A	tr 17′ Ar<br>>0.1%
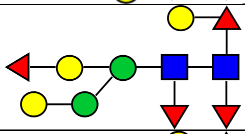	1897.73<br>H5N2F4<br>7.5-7.7<br>Supp. Fig. 9O							17.7′ Ar<br>>0.1%<br>tr 16′ A
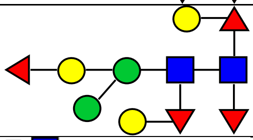	1897.73<br>H5N2F4<br>8.5							18.5′ Ar<br>0.9%<br>17.8′ A
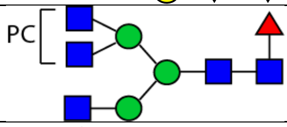	1909.74<br>H3N5F1PC1<br>11<br>Fig. 4N				tr 20.6-21.5′<br>>0.1%	20.6′<br>>0.1%<br>21.5′		
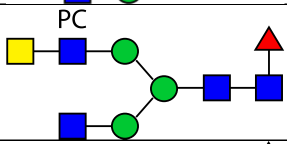	1909.74<br>H3N5F1PC1<br>>11<br>Fig. 4O	27.7′	27.8′	tr 27.7′	27.1′<br>0.9%<br>28′	27.2′<br>1.0%<br>28′		
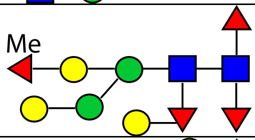	1911.75<br>H5N2F4Me1<br>6-7.2<br>Fig. 7B						16.4′ Ar<br>0.7%<br>15.5′ A	
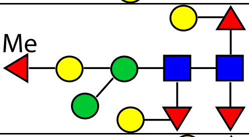	1911.75<br>H5N2F4Me1<br>8<br>Supp. Fig. 8H							17-17.7′ Ar<br>0.2%<br>16′ A
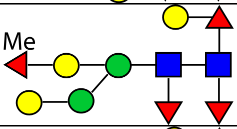	1911.75<br>H5N2F4Me1<br>9<br>Supp.Fig. 11M						18.8-19.2′ Ar<br>>0.1%	
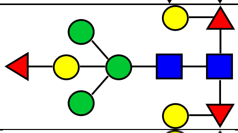	1913.73<br>H6N2F3<br>4.8<br>Supp. Fig. 9P							11.3-11.6′ Ar<br>>0.1%
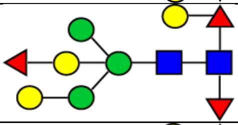	1913.73<br>H6N2F3<br>6.5-7<br>Fig. 7C						16.4′ Ar<br>0.3%<br>15.5′ A	15.6-16′ Ar<br>>0.1%<br>15.5′ A
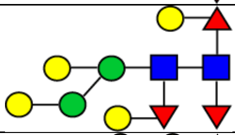	1913.73<br>H6N2F3<br>6.8-7.5<br>Fig. 7D						17.3′ Ar<br>0.2%<br>16.2′ A	17′ Ar<br>>0.1%<br>16′ A
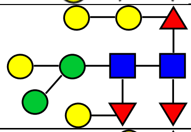	1913.73<br>H6N2F3<br>6.8-7.5<br>Fig. 7E							17′ Ar<br>>0.1%
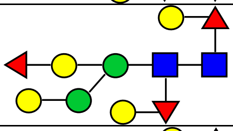	1913.73<br>H6N2F3<br>9.5					19.7′<br>>0.1%		
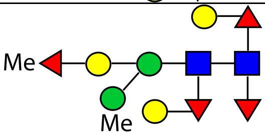	1925.76<br>H5N2F4Me2<br>8<br>Supp. Fig. 11O						18.1′ Ar<br>0.2%	
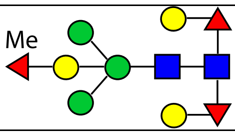	1927.74<br>H6N2F3Me<br>5<br>Fig. 7N						12.3′ Ar<br>>0.1%	

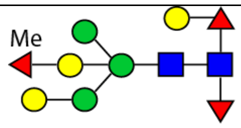	1927.74<br>H6N2F3Me<br>7.6<br>Fig. 7F						17.3’ Ar<br>1.2%<br>16.2’ A	tr 17.0’ Ar<br>>0.1%
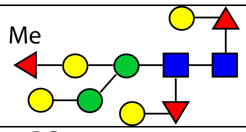	1927.74<br>H6N2F3Me<br>9-10<br>Fig. 3J				19.8’ F<br>0.2%<br>19.5 F’		19.9’ Ar	
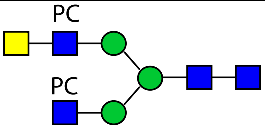	1928.74<br>H3N5PC2<br>16<br>Fig. 4D<br>Supp. Fig. 5	26-27’	26’	25-25.8’	24.3-25.8’<br>0.2%<br>25’	24.3-25.8’<br>0.1%<br>25-26’		
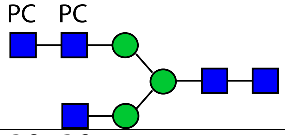	1928.74<br>H3N5PC2<br>>16				28.9’<br>>0.1%<br>26’	28.9-29.3’<br>>0.1%<br>25’		
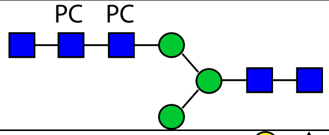	1928.74<br>H3N5PC2<br>17	27.7’	27.7’	27.7’		25.8’		
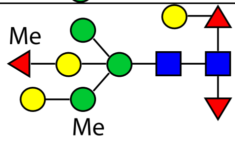	1941.76<br>H6N2F3Me2<br>11.5<br>Supp. Fig. 11P						21.2’ Ar<br>0.2%<br>20’ A	
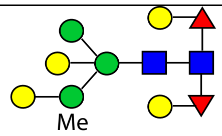	1943.74<br>H7N2F2Me1<br>5.5<br>Supp. Fig. 11Q						tr 13.6’ Ar<br>>0.1%	
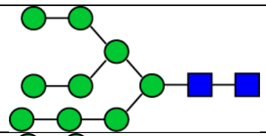	1961.71<br>H9N2<br>(Man9)<br>4.9	12.5’	12.4’	12.5’	12.1’<br>8.7%<br>10.8’	12.0’<br>10.0%<br>10.8’	12.3 Ar<br><br>11’ A	12.1’ Ar
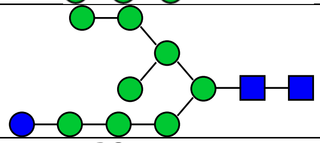	1961.71<br>H9N2<br>Man8Glc<br>5.2				13.6’<br>>0.1%	13.1’<br>>0.1%<br>11.8’		
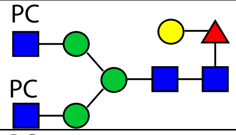	2033.77<br>H4N4F1PC2<br>>17<br>Supp. Fig. 5				28.9’<br>>0.1%	29.3’<br>>0.1%		
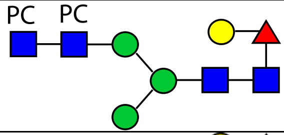	2033.77<br>H4N4F1PC2<br>>17				32.0’<br>>0.1%			
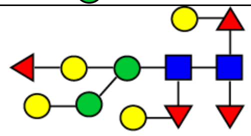	2059.78<br>H6N2F4<br>7.5-8.8<br>Fig. 7S/U						18.8’ Ar<br>>0.1%<br>17.8’ A	18.5’ Ar<br>>0.1%<br>17.8’ A
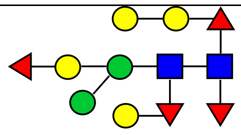	2059.78<br>H6N2F4<br>8.5<br>Fig. 7T							18.5’ Ar<br>>0.1%
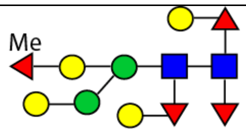	2073.80<br>H6N2F4Me1<br>8.2<br>Fig. 7G						18.1’ Ar<br>1.7%<br>17’ A	tr 17.7’ Ar<br>>0.1%

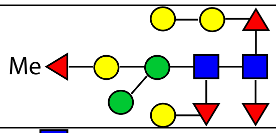	2073.80<br>H6N2F4Me1<br>8.2							tr 17.7′ Ar<br>>0.1%
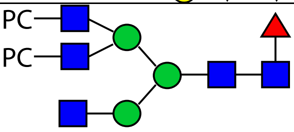	2074.80<br>H3N5F1PC2<br>15<br>Fig. 4H<br>Supp. Fig. 5				tr 22.2-23.8′<br>>0.1%	tr 23.8′<br>>0.1%		
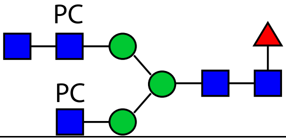	2074.80<br>H3N5F1PC2<br>>17<br>Fig. 4R<br>Supp. Fig. 5	tr 26′			27.1-28.9′ F<br>>0.1%	29.3′ F<br>0.2%		
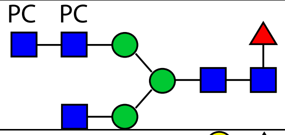	2074.80<br>H3N5F1PC2<br>>17<br>Supp. Fig. 5				30.5-31.3′<br>>0.1%<br>32.5′	31.2-32.8′<br>0.2%<br>32.5′		
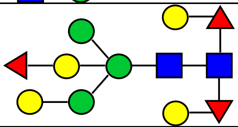	2075.78<br>H7N2F3<br>5<br>Fig 7O						tr 12.3′ Ar<br>>0.1%	
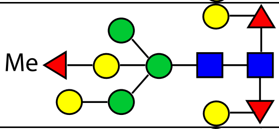	2089.79<br>H7N2F3Me1<br>5<br>Fig 7P						12.3′ Ar<br>>0.1%	
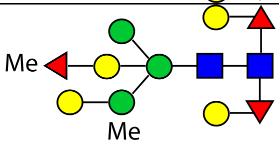	2103.81<br>H7N2F3Me2<br>6<br>Supp. Fig. 11R						15′ Ar<br>>0.1%	
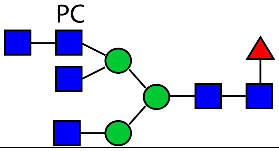	2112.82<br>H3N6F1PC1<br>>11<br>Fig. 4P				tr 20.6-21.5′<br>>0.1%	20.6-21.5′<br>>0.1%		
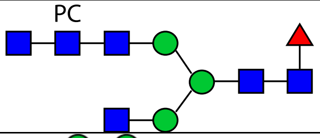	2112.82<br>H3N6F1PC1<br>>11<br>Fig. 4Q					25.8-27.2′<br>>0.1%		
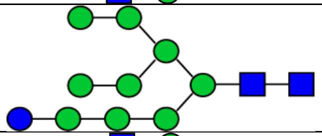	2123.76<br>H10N2<br>5.9	14.7′	13.9-14.7′	14.5′	13.6-14.4′<br>0.7%<br>13.2′	14.3-14.9′<br>0.9%<br>13.2′		
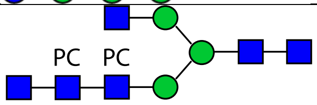	2131.82<br>H3N6PC2<br>14				24.3′<br>>0.1%			
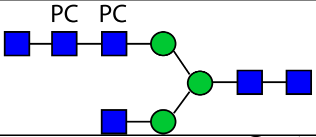	2131.82<br>H3N6PC2<br>16<br>Supp. Fig. S2M<br>Supp. Fig. 5	27′	27.8′	25.8′	25.8′<br>>0.1%<br>tr 25-26′	tr 25-25.8′<br>>0.1%<br>26′		
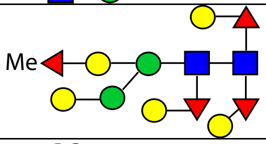	H7N2F4Me1<br>2235.85<br>5.5<br>Fig. 7Q						13.1-13.6′ Ar<br>>0.1%	
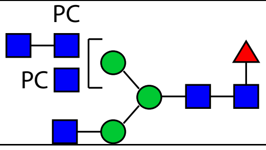	2277.88<br>H3N6F1PC2<br>>17<br>Fig. 4I				23.8′<br>>0.1%			

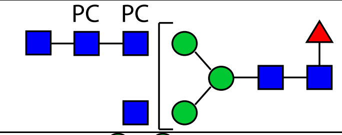	2277.88<br>H3N6F1PC2<br>>17<br>Fig. 4S<br>Supp. Fig. S5				27.9-31.3’<br>>0.1%	25.8-31.2<br>0.1%		
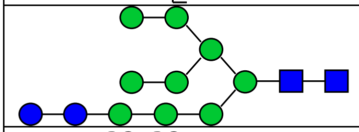	2285.81<br>H11N2<br>6					14.9’<br>>0.1%		
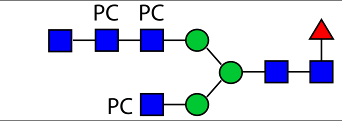	2442.94<br>H3N6F1PC3				31.3-32’<br>>0.1%			
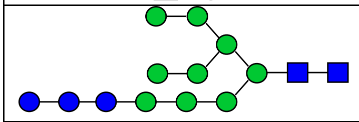	2447.87<br>H12N2				13.6’<br>>0.1%	nd 13.6’		
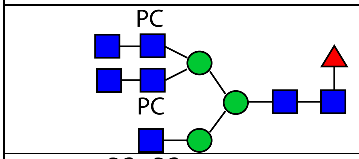	2480.96<br>H3N7F1PC2<br>15<br>Fig. 4J<br>Supp. Fig. S5				22.2-23.8’<br>>0.1%	23.8’<br>>0.1%<br>tr 23.5-25’		
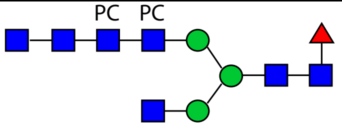	2480.96<br>H3N7F1PC2<br>>15<br>Fig. 4T<br>Supp. Fig. S5					28.9-31.2’<br>>0.1%		

Supplementary Table S2: Summary of O-glycan compositions and analyses. Peptic glycopeptides remaining after PNGase A/F digestion from *C. elegans* cultures were subject to reductive β-elimination and analysed without further derivatization by LC-ESI-MS (for example spectra, see **Figure 8** and **Supplementary Figure S13**). The calculated and observed *m/z* values as well as the percentage occurrence are shown. Of the embryo samples, there is a potential shift to simpler structures especially in the t3208 strain, while phosphorylcholine-modified O-glycans (rows in grey) are far less detectable in the L4 larvae and *pmk-1* mixed culture (in part detected, but not fragmented as indicated by an asterisk), whereas $Hex_4HexNAc_1$ is more pronounced suggestive of some shifts in O-glycosylation during development. The definition of HexA as opposed to HexMe is based on the absence of putatively methylated structures in the *samt-1* strain, lacking a proposed *S*-adenosylmethionine transporter, which was analysed in parallel.

m/z (calc)	*RT*	*Composition*	*N2emb*		*e2144a*		*t3208a*		*N2L4 liq*		*N2L4 plate*		*pmk-1 mix*	
384.15	9.50	Hex1HexNAc1	384.25	18.3	384.17	25.6	384.25	38.6	384.25	8.2	384.08	8.9	384.25	10.3
546.20	15.52	Hex2HexNAc1	546.25	1.7	546.25	2.1	546.25	3.0	546.17	5.2	546.25	7.0	546.25	6.2
560.18	12.36	Hex1HexNAc1HexA1 (branched)	560.25	12.0	560.25	12.7	560.25	7.7	560.25	2.0	560.25	0.8	560.33	1.3
560.18	16.28	Hex1HexNAc1HexA1 (linear)	560.25	6.5	560.25	2.3	560.25	4.3	560.25	1.4	560.25	0.8	560.17	2.2
692.26	21.14	Hex2HexNAc1dHex1	692.33	27.5	692.25	12.7	692.42	14.7	692.25	14.0	692.25	7.6	692.25	9.4
692.26	30.30	Hex2HexNAc1dHex1	692.33	1.6	692.33	1.5	nd	0.0	692.33	8.0	692.25	4.3	692.33	2.5
706.24	21.45	Hex2HexNAc1dHex1 Me	706.33	1.5	nd	0.0	nd	0.0	706.25	1.4	706.33	0.8	706.33	1.5
708.26	17.08	Hex3HexNAc1	708.25	0.4	708.33	0.0	708.33	0.0	708.25	2.8	708.25	1.9	708.25	0.0
722.24	19.55	Hex2HexNAc1 HexA1	722.25	1.3	722.33	5.2	722.33	3.6	722.33	1.7	722.25	3.9	722.25	7.2
749.28	19.07	Hex2HexNAc2	749.25	0.5	749.33	0.6	749.25	0.8	749.25	0.0	749.42	0.0	749.25	0.0
766.26	17.48	HexNAc2HexA1PC1	766.25	1.4	766.33	1.1	766.25	0.8	766.33*	0.1	766.33*	0.1	nd	0.0
854.31	24.77	Hex3HexNAc1dHex1	854.33	4.1	854.33	2.1	854.33	3.4	854.33	4.8	854.33	2.5	854.33	4.9
870.31	17.72	Hex4HexNAc1	870.33	11.9	870.42	18.8	870.42	12.3	870.33	35.4	870.50	49.6	870.33	40.8
884.29	19.46	Hex3HexNAc1 Hex1Me	884.33	1.6	884.25	2.1	884.25	1.7	884.25	5.9	884.42	3.2	884.33	3.6
884.29	20.89	Hex3HexNAc1 HexA1	884.42	4.1	884.33	7.7	884.25	5.9	884.33	4.5	884.33	2.8	884.33	4.6
928.32	18.53	Hex1HexNAc2HexA1PC1	nd	0.0	nd	0.0	nd	0.0	nd	0.0	nd	0.0	928.33	0.3
928.32	22.83	Hex1HexNAc2HexA1PC1	928.33	0.7	928.42	0.3	928.42	0.8	nd	0.0	nd	0.0	928.25	0.9
969.34	17.64	HexNAc3HexA1PC1	969.42	3.4	969.42	2.6	969.25	0.8	969.33*	0.1	nd	0.0	nd	0.0
1046.34	19.46	Hex4HexNAc1 HexA1	1046.33	1.3	1046.42	2.1	1046.42	1.3	1046.33	4.6	1046.42	5.7	1046.33	4.2
1134.40	16,84	HexNAc3HexA1PC2	1134.42	0.2	1134.33	0.3	1134.42	0.3	nd	0.0	nd	0.0	nd	0.0
				100.0		100.0		100.0		100.0		100.0		100.0

Supplementary Figure S1: Preparation of recombinant almond PNGase A. The sweet almond N-glycosidase A was PCR amplified from cDNA prepared from a Sardinian almond using primers PNG-Mel-BamHI-for (GATGATGGATCCATGAAATTCTTAGTCAACGTTGC) and PNG-IZT-SalI-rev (GATGATGTCGACTTAGTCAGATAAACTCAATGGTG). The product was digested with BamHI / SalI and ligated into a pKL vector cut with the same enzymes, resulting in pKL-PNG. This plasmid was inserted into the Tn7 site of DH10MultiBacY cells, followed by generation of recombinant *Autographa californica* nucleopolyhedroviruses by standard procedures. Viral titres were determined by plaque assay using 10-fold dilution series (n=3). *Trichoplusia ni* BTI-TN5B1-4 "High Five" cells ("Hi5"; ATCC CRL-10859; Wickham and Nemerow 1993) were grown in IPL-41 medium (SAFC Biosciences, St. Louis, USA) containing yeast extract and a lipid mixture supplemented with either 0% or 3% foetal calf serum (FCS) at 27°C using T-flasks. The cells were transferred to shaker flasks with supplemented IP-L41 medium without FCS two days prior to infection. For infection, $5x10^7$ cells were spun down at 900g for 10 min, resuspended in 50 mL fresh IP-L41 medium and infected with a multiplicity of infection of 5. Cells were harvested 3 days post infection by centrifugation for 10 min at 3000 g and the supernatant immediately mixed with Ni-NTA beads at 4°C while shaking for 2 h. Subsequently, the beads were filled into an Econocolumn and washed with 15 mL washing buffer (20 mM sodium phosphate, 0.5 M NaCl, 40 mM imidazole, pH 7,4). PNGase was eluted stepwise with 0.5 mL aliquots of elution buffer (20 mM sodium phosphate, 0.5 M NaCl, 500 mM imidazole, pH 7.4). Imidazole was removed by exchanging to McIlvaine buffer pH 5.5, using Vivaspin 6 columns (30,000 MWCO). The purified enzyme (1 µl) was assayed using 0.25 nmol of dabsyl-MM-glycopeptide (2 µL of 0.125 nmol/µL) mixed with 0.25 µL McIlvaine buffer pH 4.5. After incubation for 1 h at 37°C, an aliquot of 0.5 µL was removed and the reaction was continued for 23 h. Reaction aliquots were heat inactivated at 95°C for 5 min, diluted with water and subject to MALDI-TOF-MS using α-cyanohydroxycinnamic acid as matrix. (**A-B**) SDS-PAGE of fractions of His-tag purified PNGase A was followed by silver staining or Western blotting on nitrocellulose with anti-His6, showing no indication of proteolytic cleavage into two subunits, unlike the natural almond enzyme (Altmann et al., 1998). (**C**) Deduced protein sequence of PNGase A which is nearly identical to Uniprot entry PNAA_PRUDU. (**D**) MALDI-TOF-MS of a dabsyl-MM-glycopeptide incubated overnight in the absence or presence of purified recombinant PNGase A. Chromatograms of mixed *C. elegans* N-glycomes released by native and recombinant PNGase A showed no obvious differences.

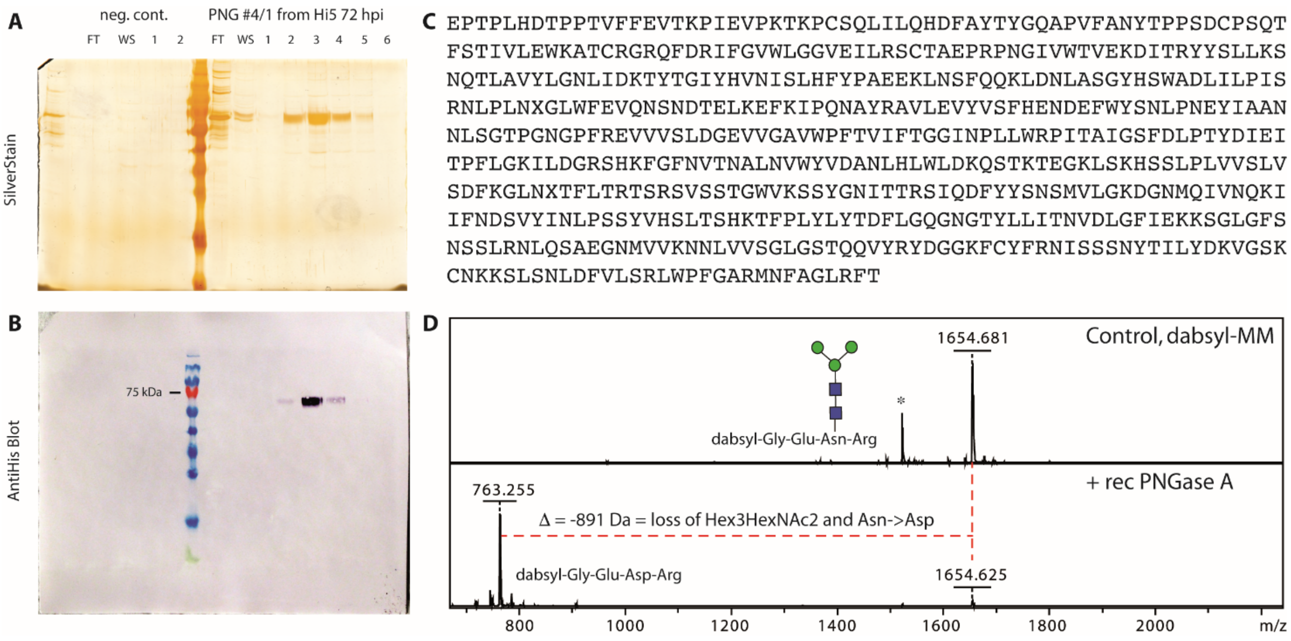

Supplementary Figure S2: MALDI-TOF MS of the individual RP-HPLC fractions of PNGase A-released N-glycans from N2 embryos. Glycans are annotated on the basis of retention time and MS/MS using the Symbolic Nomenclature for Glycans; example MS/MS are shown and selected fractions were also subject to confirmatory glycosidase treatments. The glycans are annotated as $[M+H]^+$ ions, but are significantly detected as $[M+K]^+$; *m/z* values in red correspond to impurities. In comparison to the wild-type fractions shown here, some minor core α1,3-fucosylated structures were not detected in the two mutants, *e.g.*, isomers of *m/z* 1281 (7.5 g.u.), 1443 (9 g.u.), 1459 (2.8 g.u.) or 1589 (5.2 g.u.). Other minor structures were, on the other hand, only found in one mutant (*e.g.*, galactosylated glycans of *m/z* 1500 and 1605 in *apx-1* or a phosphorylcholine-modified one of *m/z* 1706 in *glp-1*).

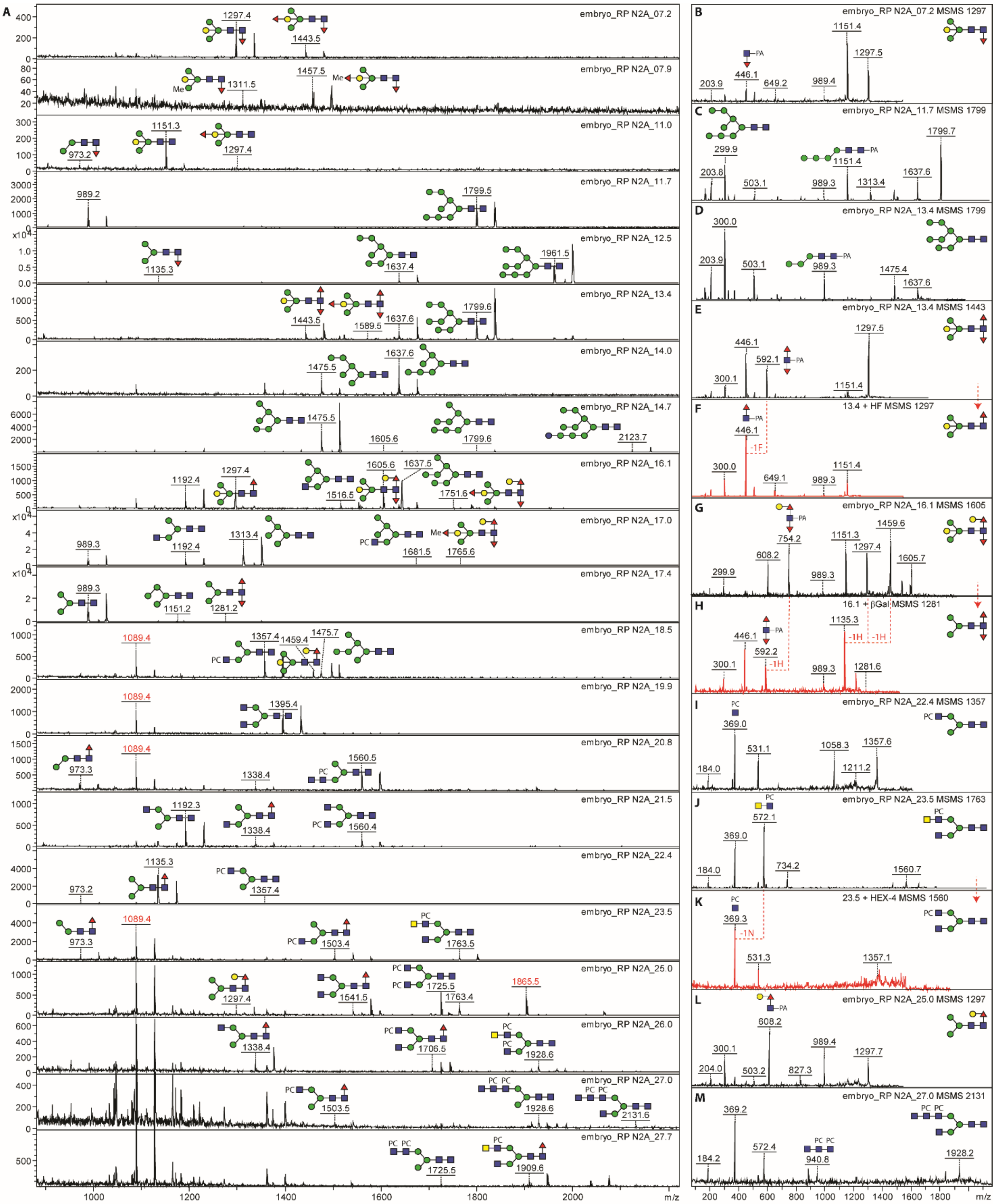

Supplementary Figure S3: RP-HPLC of PNGase F-released N-glycans from *C. elegans* L4 larvae. Chromatograms for the wild-type L4 liquid- and plate-grown larvae are shown annotated with dextran hydrolysate as external calibrant (in glucose units, g.u.) and with the structures found on the basis of MS, MS/MS and digestion data. These data from the first preparations are comparable, but not identical to those for the second preparations (see ***Figure 2*** in the main text). Methylated glycans are highlighted in grey boxes

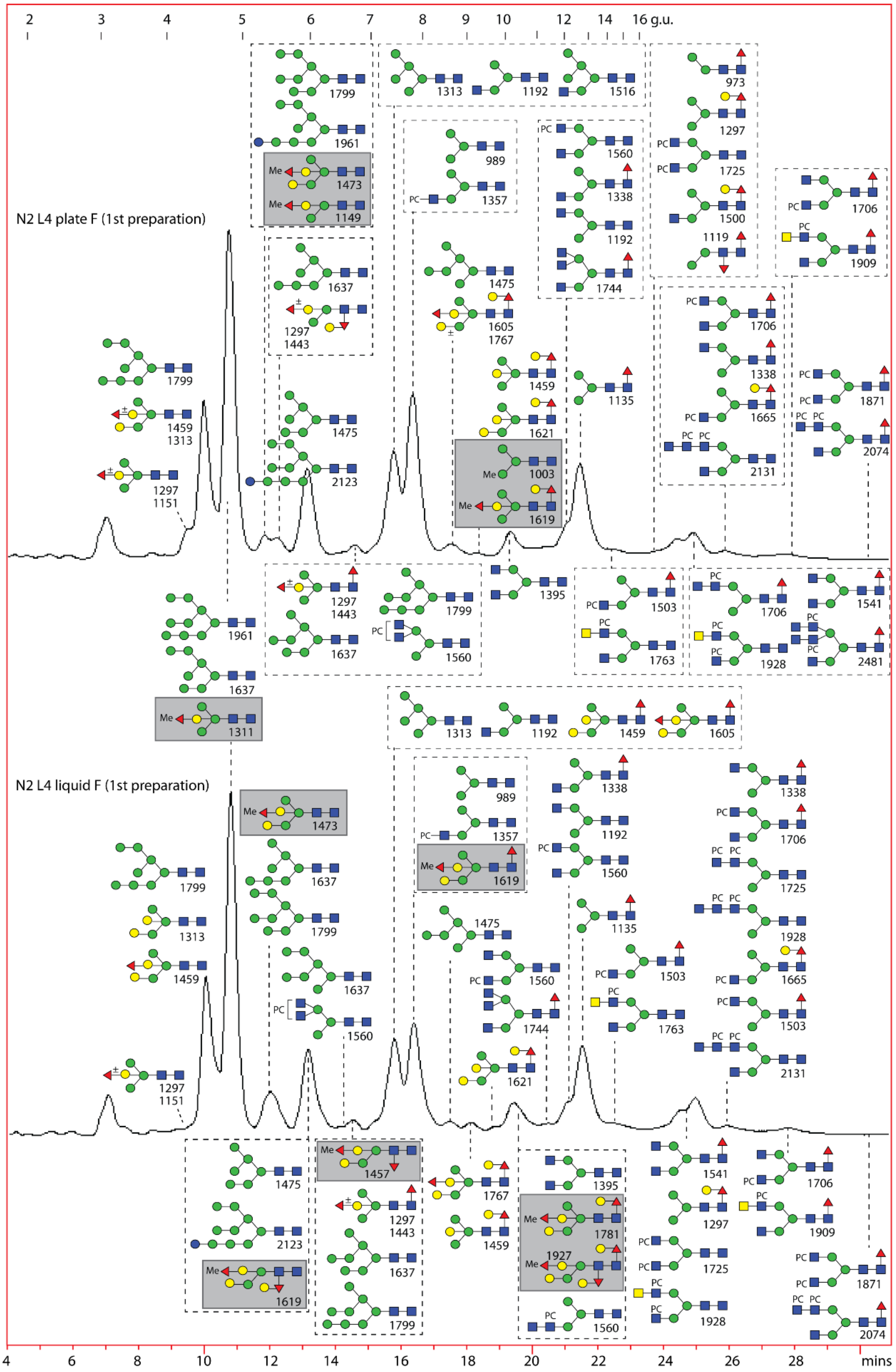

Supplementary Figure S4: MALDI-TOF MS of the individual RP-HPLC fractions of PNGase F-released N-glycans from L4 larvae (first preparations). Glycans are annotated on the basis of retention time, MS/MS and selected digestion data; for the corresponding RP-HPLC chromatograms, see ***Supplementary Figure S3***. The glycan of *m/z* 1865 (25 minutes; $Hex_5HexNAc_4Fuc_1$) is derived from the porcine pepsin used for proteolysis; for reasons of space, five glycans are depicted beside the legend. The comparison shows that even fractions of the same or similar retention time do not always contain the same set of glycan structures in the liquid and plate-cultivated samples. The data are generally comparable to those for the PNGase F-released glycans (second preparation; **Figures 2-4**) shown in the main text. Comparing fractions of the same or similar retention time indicates that α1,2-fucosylation of the bisecting residue results in slightly earlier RP-HPLC elution, whereas α-galactosylation of the α1,3-mannose rather slightly extends elution time; methylation of the α1,2-fucose, core α1,6-fucosylation, β-galactosylation of the core α1,6-fucose, modification of the distal GlcNAc or antennal phosphorylcholine residues result in significantly higher retention on a standard ODS C18 column. Also oligomannosidic isomers can be separated as previously reported (Hykollari *et al.*, 2016, *Mol. Cell. Proteomics,* 15, 73–92).

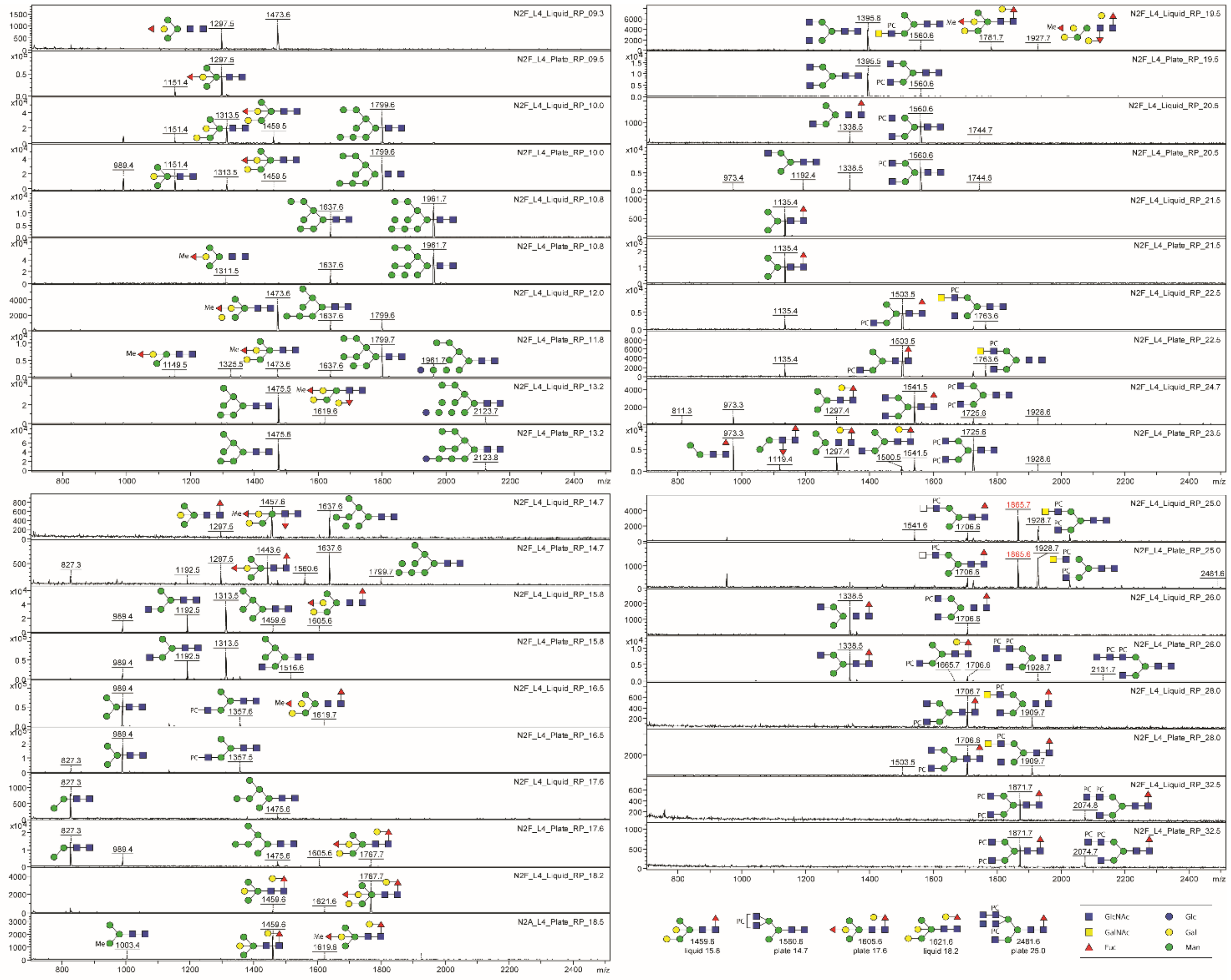

Supplementary Figure S5: 2D-HPLC and MALDI-TOF MS/MS of PNGase F-released N-glycans from plate-grown L4 larvae (second preparation). 30% of the PNGase F-release glycan pool was applied to the AS3 HIAX column **(A)** and three neighbouring fractions containing glycans of *m/z* 1725-2440 (see inset in A for MALDI-TOF MS) predicted to be modified with two phosphorylcholine residues were then pooled and applied to an RP-amide column **(B)**. All fractions were subject to MALDI-TOF MS/MS of $[M+H]^+$ ions (**C**), whereby the annotated structures proposed on the basis of MS/MS are verified in part also by specific hexosaminidase treatments: specifically those of *m/z* 1928 (17.5 mins; HEX-4 removal of 1 GalNAc), 2131 (14.9 mins; chitinase removal of 1 HexNAc), 2277 (18.8 mins; chitinase removal of 1 HexNAc) and 2481 (19.8 mins; chitinase removal of 2 HexNAc residues). The L4 plate Ar chromatogram (see also ***Supplementary Figure S8***) is shown for comparison in panel A indicating the relatively low amount of glycans released by PNGase Ar following the PNGase F digest. HIAX separates primarily by size, but PC-containing structures elute somewhat later than oligomannosidic glycans of similar mass, but on the RP-amide column they elute earlier than on the C18 column used in the rest of the study (see ***Figure 2*** in the main text or ***Supplementary Figure S3***). Previous Q-TOF CAD-MS/MS and FAB-MS data has indicated that phosphorylcholine is 6-linked to HexNAc residues of perdeuteroacetylated *C. elegans* N-glycans and that HF treatment removing PC results in increased abundance of detected permethylated $Hex_3HexNAc_{3-7}$ structures as well as related $HexNAc_{1-3}$ fragment ions (Haslam *et al.*, 2002, *Biochem. Soc. Symp*. 69, 117-134).

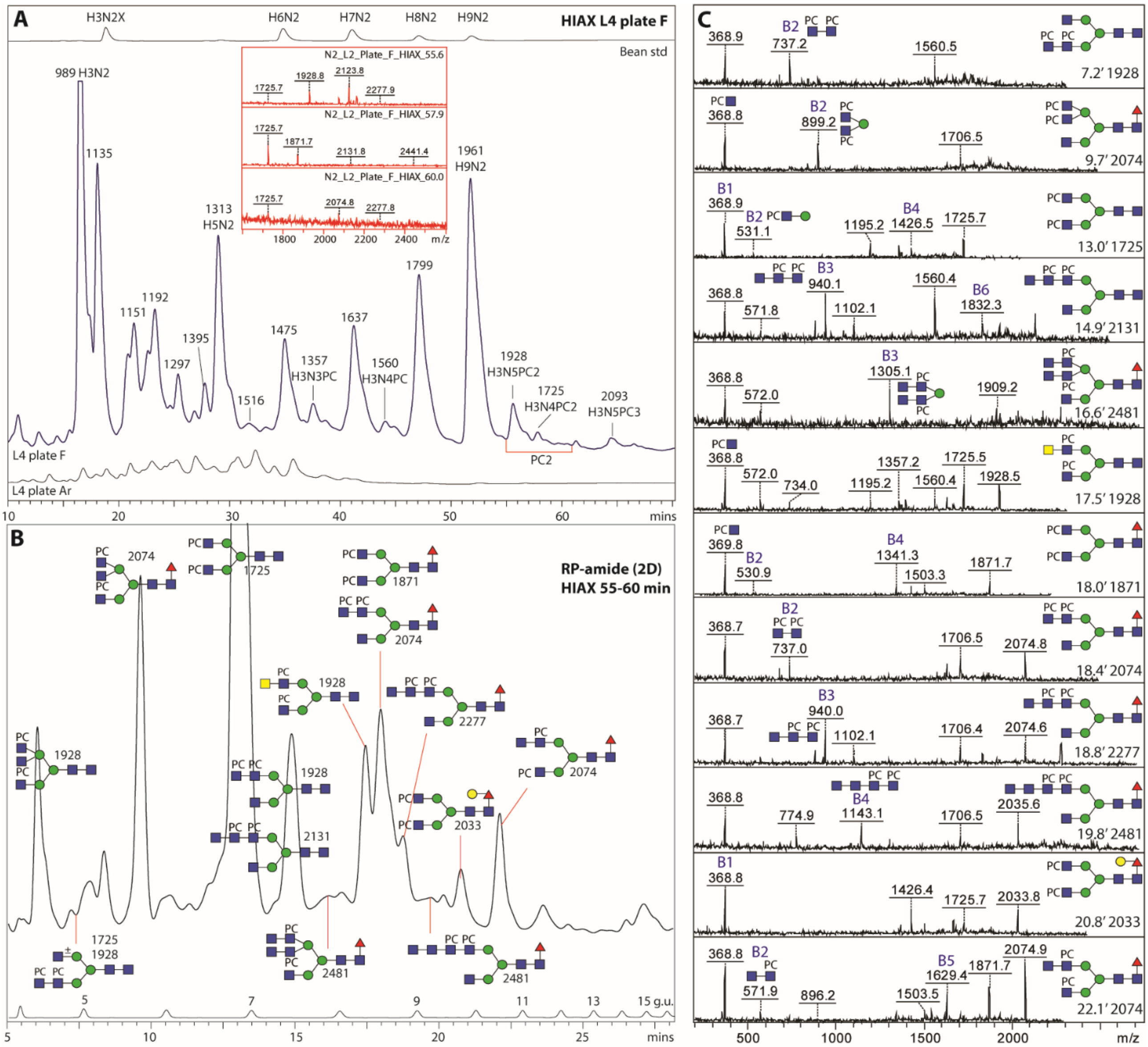

Supplementary Figure S6: RP-HPLC of PNGase A-released N-glycans from *C. elegans* L4 larvae. Chromatograms for the wild-type L4 liquid- and plate-grown larvae are shown annotated with dextran hydrolysate as external calibrant (in glucose units, g.u.) and with the structures found on the basis of MS, MS/MS and digestion data; the most dominant glycans in each fraction are shown uppermost. These data from first preparations are comparable, but not identical to those for the second preparations (see ***Figure 5*** in the main text), particularly as PNGase Ar was used for the latter, an enzyme which releases N-glycans with a GalαFucα3-substitution of the proximal core GlcNAc (associated with an α-galactosidase sensitive core Y_1 fragment of *m/z* 916). There is more modification of the distal (second) GlcNAc in the plate-grown larvae and more methylation of fucose (methylated glycans are highlighted in grey boxes) and α-galactosylation of mannose in the liquid-grown larvae.

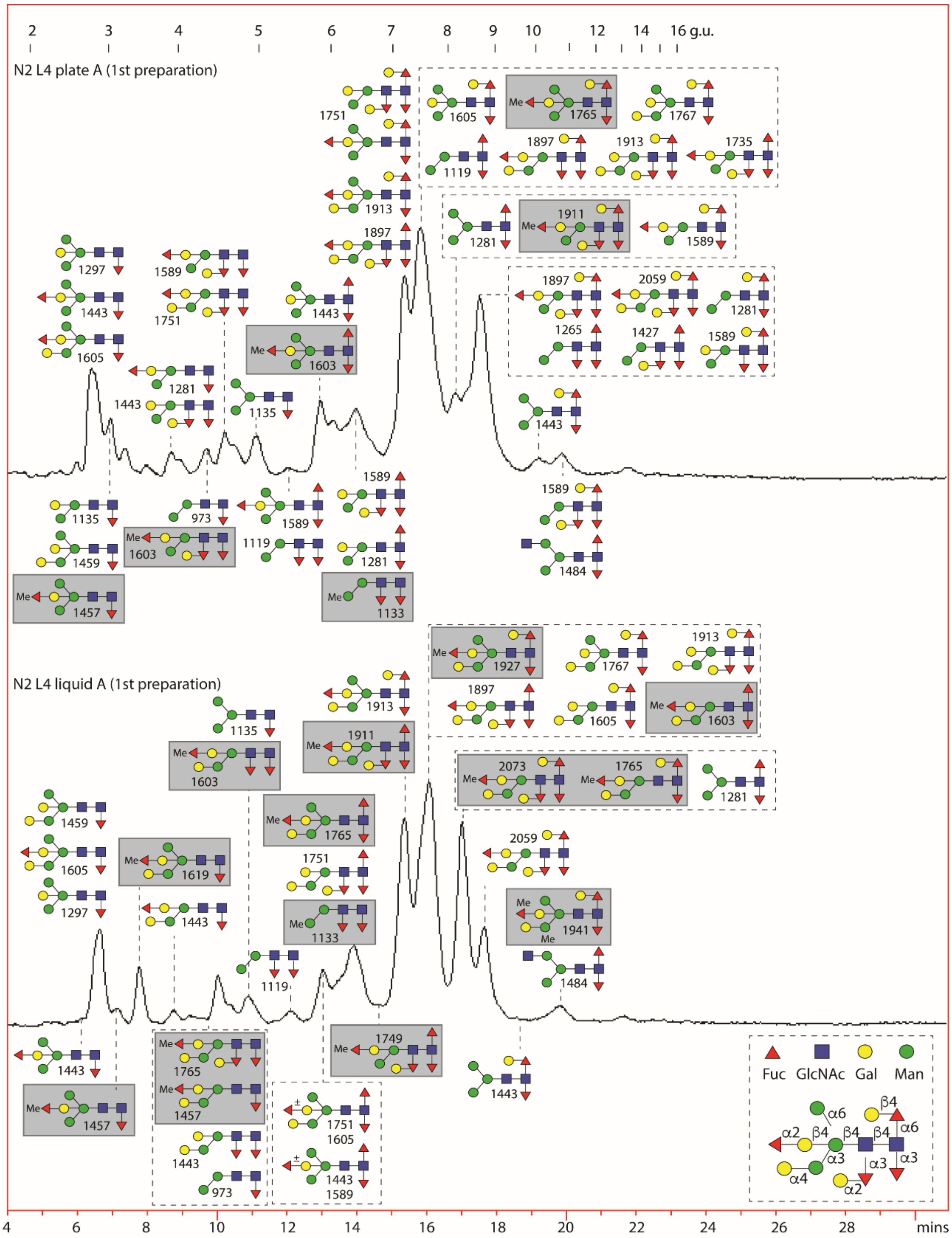

Supplementary Figure S7: MALDI-TOF MS of the individual RP-HPLC fractions of PNGase A-released N-glycans from L4 larvae (first preparations). Glycans are annotated on the basis of retention time, MS/MS and selected digestion data; for the corresponding RP-HPLC chromatograms, see ***Supplementary Figure S6***. The comparison shows that even fractions of the same or similar retention time do not necessarily contain the same set of glycan structures in the liquid and plate-cultivated samples. The data are in general comparable to those for the PNGase Ar-released glycans (second preparation; ***Figures 5-7***) shown in the main text with the primary exception that glycans with a proximal core GlcNAc decorated with two GalFuc modifications (core Y_1 fragment of *m/z* 916) are absent from the PNGase A-released subglycomes; this is due to the more restricted substrate specificity of the native enzyme as compared to the recombinant one. Comparing fractions of the same or similar retention time indicates that α1,2-fucosylation of the bisecting residue results in slightly earlier RP-HPLC elution, whereas α-galactosylation of the α1,3-mannose or methylation of the α1,2-fucose rather slightly extend elution time; both β-galactosylation of the core α1,6-fucose and modification of the distal GlcNAc result in significantly higher retention.

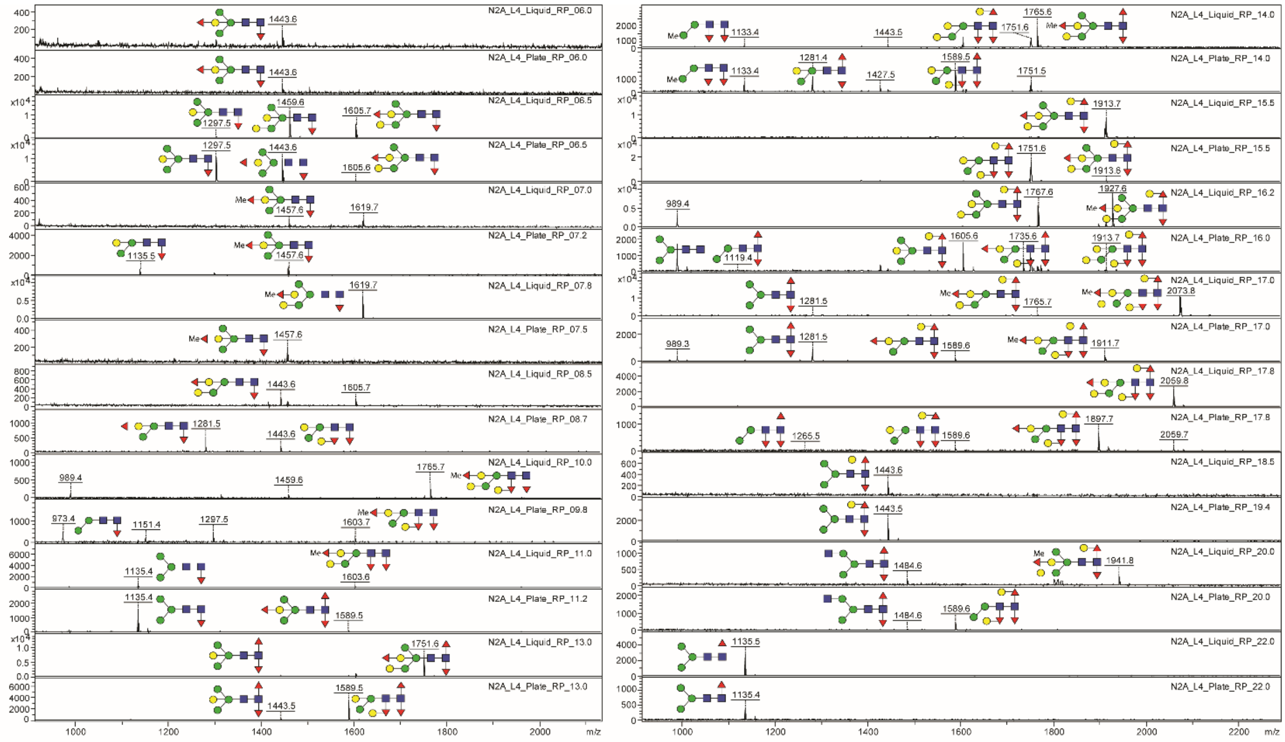

Supplementary Figure S8: 2D-HPLC and MALDI-TOF MS/MS of PNGase Ar-released N-glycans from plate-grown L4 larvae (second preparation). 30% of the PNGase Ar-release glycan pool was applied to the AS3 HIAX column **(A)** and three neighbouring fractions containing predominantly glycans of *m/z* 1605 and 1751 (see inset in A for MALDI-TOF MS) were then pooled and applied to an RP-amide column **(B)**. MALDI-TOF-MS for five RP-amide fractions are shown (inset in B) with corresponding MALDI-TOF-MS/MS before and after selected confirmatory *A. niger* β-galactosidase, HF or α1,2-fucosidase treatments **(C-L)**. The data for the major 2D-HPLC glycans correspond to those in the most dominant RP-HPLC fractions shown in the main text (**Figure 5**). HIAX separates primarily by size, but core modified structures elute somewhat earlier than oligomannosidic glycans of similar mass. To be noted is that order of elution on the RP-amide column is similar for these glycans as for the classical C18 column (see ***Figure 5*** in the main text or ***Supplementary Figure S6***), but that the oligomannosidic glycans elute later than the glycans with complex core modifications of similar mass (the *m/z* 1605 glycan co-elutes with $Man_5GlcNAc_2$, *m/z* 1313), while methylated glycans elute relatively early (e.g., the *m/z* 1911 glycan elutes in the same HIAX fraction as the *m/z* 1751 glycan, but later on RP-amide).

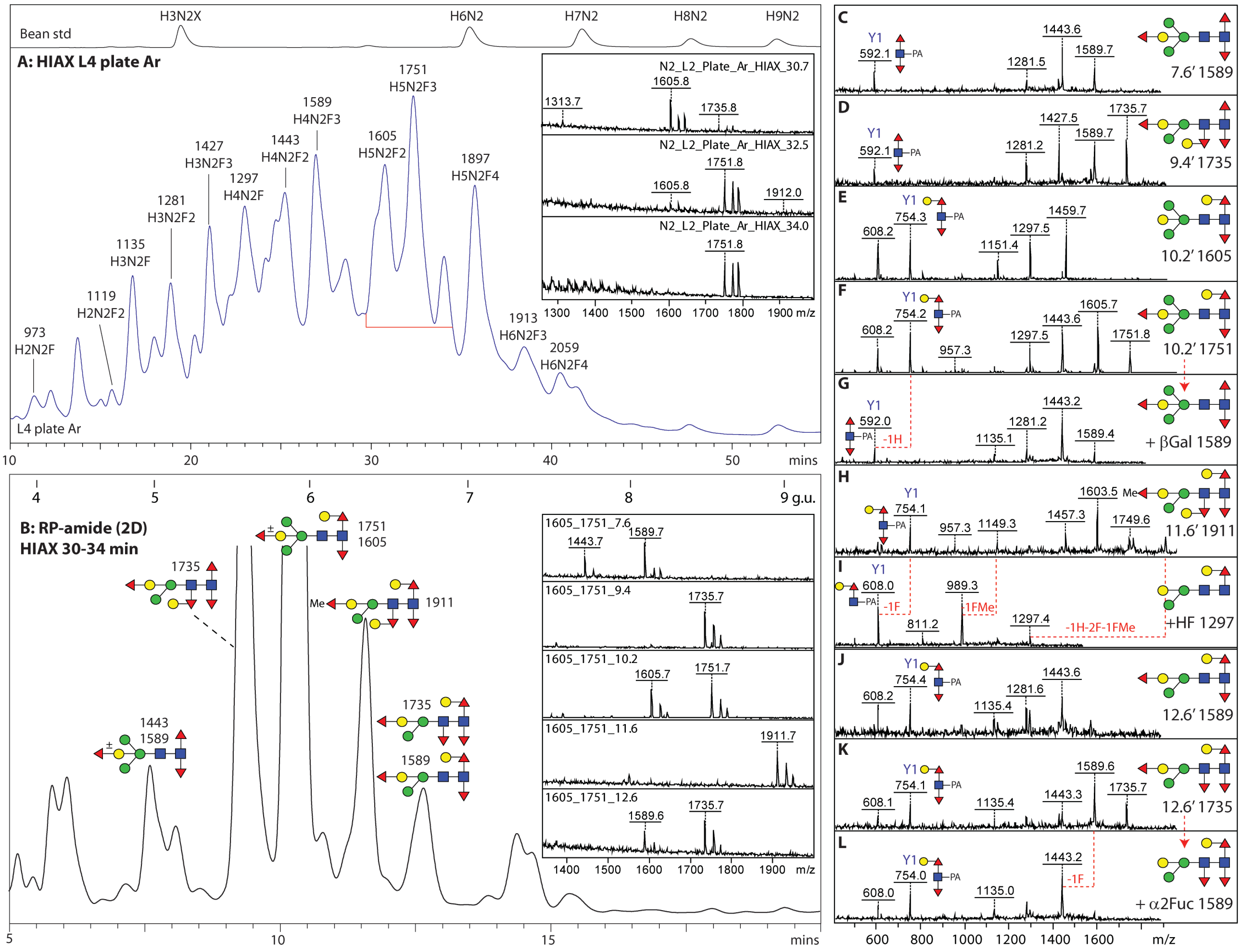

Supplementary Figure S9: Further example MS/MS of unmethylated N-glycans from liquid- and plate-cultivated L4 larvae. PNGase Ar- or A-released glycans from plate- or liquid-cultivated L4 wild-type larvae detected in the stated RP-HPLC fractions were analysed by MALDI-TOF MS/MS. The different Y_1 (*m/z* 446, 592, 754 and 916; $HexNAc_1Fuc_{1\text{-}2}Hex_{0\text{-}2}PA$) or Y_2 fragments (*m/z* 795; $HexNAc_2Fuc_2$-PA) indicate different degrees of modification of the proximal and distal core GlcNAc residues as well as isomeric separation by RP-HPLC. The order of serial losses (either 146, 162 or 308; i.e., Fuc, Hex or HexFuc), the comparative elution time and the noted sensitivity to chemical or enzymatic treatments (hydrofluoric acid, α1,2-fucosidase or α- or β-galactosidase; losses of either Fuc (F), Hex (H) or HexFuc (HF) or resistance (res)) are also considered for data interpretation. The tendency of HF-sensitive α1,3-fucose residues to be lost upon MS/MS as compared to the partially HF-sensitive α1,2- and HF-resistant α1,6-fucose has been observed in earlier studies; the loss of a hexose residue before loss of a second fucose residue is indicative for GalFuc motifs on the core, while the α-galactose on the α1,3-mannose and the β-galactose on the core α1,6-fucose are apparently more labile in MS/MS than the bisecting β-galactose. The GalFuc motif on the distal core GlcNAc of some isomers (panels D, I and M) has been previously observed in clade V nematodes (*C. elegans*, *H. contortus* and *O. dentatum*) and, due to the known specificity of the nematode FUT-6 α1,3-fucosyltransferase is only present in the absence of the α1,6-mannose residue (Yan *et al.*, 2013, *J. Biol. Chem.*, 288, 21015–21028). For further MS/MS of *m/z* 1443, 1589, 1751 and 1913 isomers, refer to respectively to ***Figures 6 I/J, 6 K-T, 7 J-L und 7C-E*** in the main text. For MS/MS of a methylated form of *m/z* 1427 (i.e., *m/z* 1441), refer to ***Supplementary Figure S11C***.

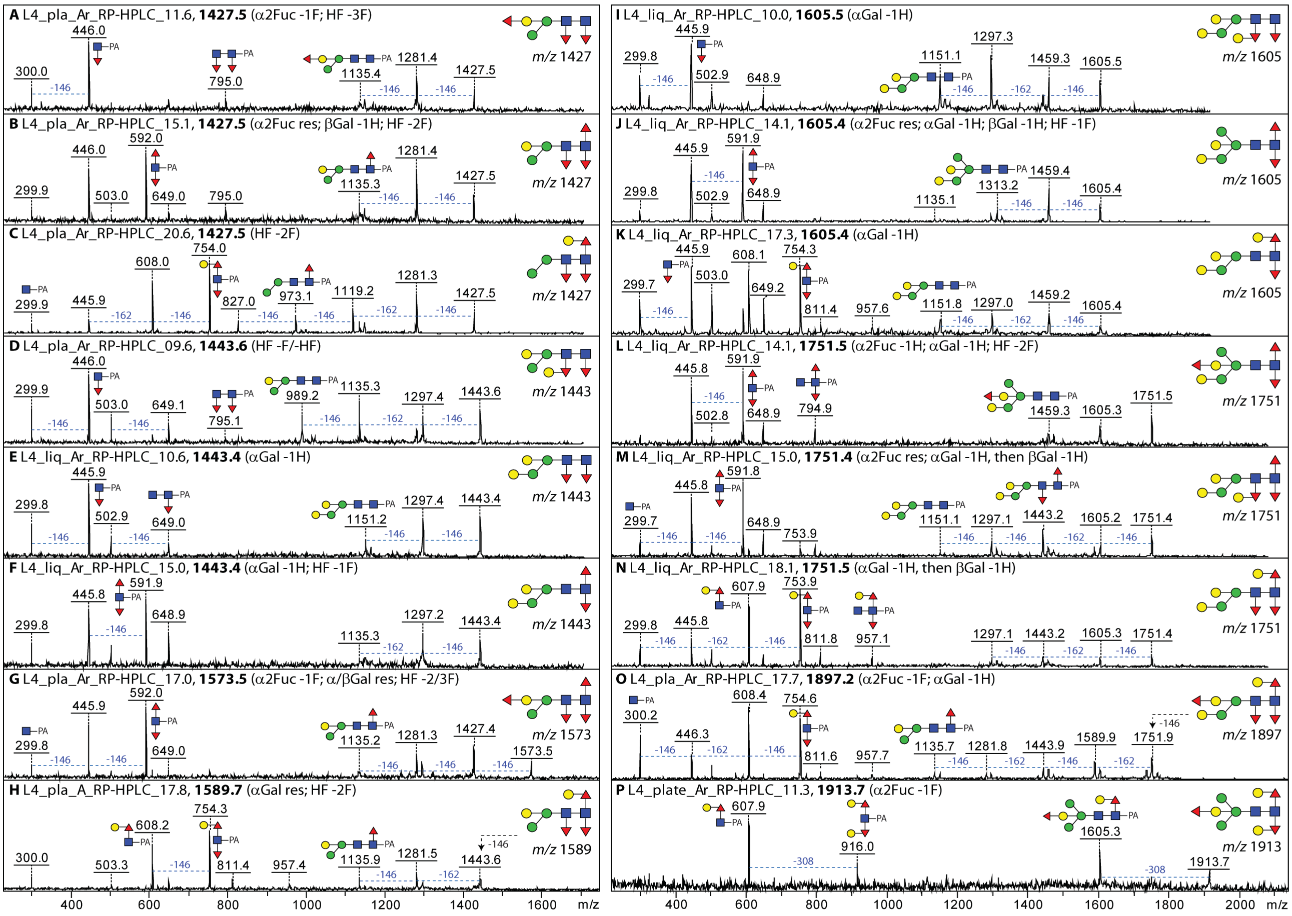

Supplementary Figure S10: MALDI-TOF MS and MS/MS of three isomers of $Hex_5HexNAc_2Fuc_3Me_1$ before and after α-galactosidase treatment. RP-HPLC fractions containing PNGase Ar-released glycans from liquid-cultivated wild-type L4 larvae were analysed by MALDI-TOF MS and MS/MS before and after treatment with coffee bean α-galactosidase. The different Y_1 fragments (*m/z* 446, 592 and 754; $Hex_{0-1}HexNAc_1Fuc_{1-2}$-PA) indicate different degrees of modification of the proximal core (reducing-terminal) GlcNAc; none of the core fucose residues are methylated, but the β-galactosidase-sensitive *m/z* 1765 glycan in the 18.1 minute fraction with the *m/z* 754 Y_1-fragment is galactosylated, while the one in the 15.0 minute fraction with the *m/z* 592 Y_1-fragment is β-galactosidase-resistant and therefore not galactosylated on the core α1,6-fucose. On the other hand, two of the three fractions were treated with α1,2-fucosidase and no mass shift was observed, indicating that only the fucose attached to the bisecting galactose was methylated. The bisecting β-galactose attached to the β1,4-mannose and the antennal α-galactose α1,4-linked to the α1,3-mannose has been proven also by NMR and ESI-MS^n of N-glycans from mutant adult *C. elegans* (Yan *et al.*, 2015, *Mol Cell Proteomics* 14, 2111-2125). The occurrence of *O*-methylated fucose, 2-fucosylation of galactose and a bisected 3,4,6-substituted mannose was also indicated by GC-MS data on the full *C. elegans* N-glycome (Haslam *et al.*, 2002, *Biochem. Soc. Symp*. 69, 117-134), while NMR data show the presence of 2-*O*-methylfucose on *C. elegans* glycolipids (Griffitts *et al*., 2005, *Science*, 307, 922-925). Selected fragments and $\Delta m/z$ are annotated; the MS spectra show the occurrence of sodium and potassium adducts, but only the protonated molecular ions were subject to MS/MS. The order of elution (only α1,3-fucosylated on the core, α1,3/α1,6-difucosylated core and the β-galactosylated difucosylated core) is also in accordance with previous studies. For MS/MS of the corresponding non-methylated forms of the first two *m/z* 1765 isomers, refer to ***Figure 7J*** and ***Supplementary Figure S9L***, whereas the MS/MS of two of the co-eluting non-α-galactosylated *m/z* 1603 glycans are shown in ***Supplementary Figure S11 D and F***.

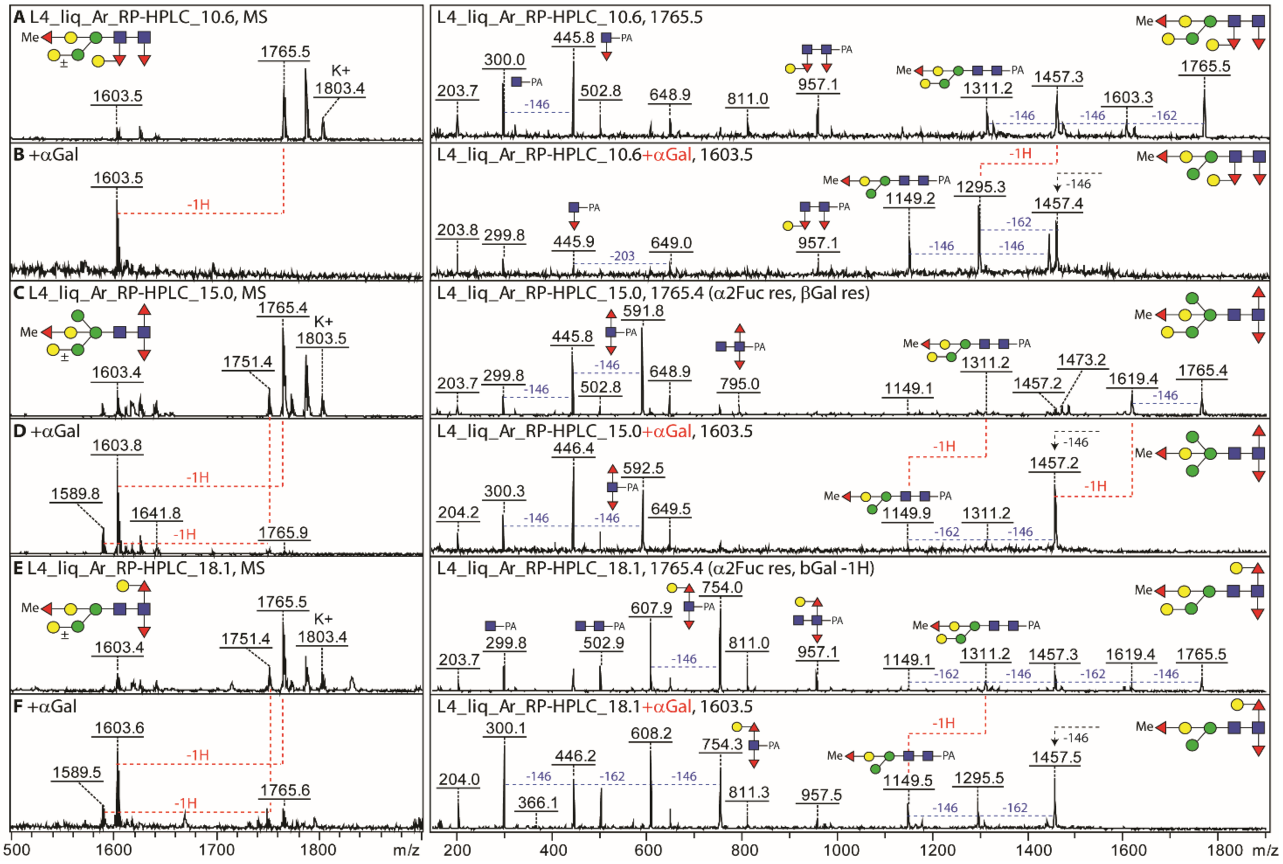

Supplementary Figure S11: Further example MALDI-TOF MS/MS of naturally-methylated PNGase Ar- or A-released glycans from liquid-cultivated L4 wild-type larvae detected in the stated RP-HPLC fractions. The different Y_1 (*m/z* 446, 592, 754 and 916; $HexNAc_1Fuc_{1-2}Hex_{0-2}PA$) or Y_2 fragments (*m/z* 795, 957 or 1103; $HexNAc_2Fuc_{2-3}Hex_{0-1}$-PA) indicate different degrees of modification of the proximal and distal core GlcNAc residues. The order of serial losses (either 146, 160, 162 or 308; i.e., Fuc, MeFuc, Hex or HexFuc), the comparative elution time and the noted or shown sensitivity to chemical or enzymatic treatments (hydrofluoric acid, microbial α1,2-fucosidase, coffee bean α-galactosidase or *A. niger* β-galactosidase; losses of either Fuc (F), MeFuc (FMe), Hex (H) or HexFuc (HF) or resistance (res), exemplified in panels *G* and *N*)) were also considered for data interpretation. The methylfucose residues are concluded to substitute the bisecting β-galactose residue on the basis of their resistance to α1,2-fucosidase, but sensitivity to hydrofluoric acid (panel *G*), their lack of association with Y_1 or Y_2 fragments, their relative low lability in MS/MS and previous ESI-MS^n data, while their removal has been shown to be a pre-requisite for enzymatic removal of the underlying bisecting β-galactose (Yan *et al*., 2015, *Mol Cell Proteomics* 14, 2111-2125). Where no $\Delta m/z$ of 160 is observed in the MS/MS spectra and/or no loss of 160 occurs upon HF treatment, the methyl group is concluded to be on the α1,3-mannose; in some instances, the methylated mannose is substituted by an α-galactose residue. Others' monosaccharide analyses indicated the occurrence of 2-*O*-methylfucose and 3-*O*-methylmannose on *C. elegans* N-glycans (Wohlschlager *et al.*, 2014, *Proc. Natl. Acad. Sci. USA*, 111, E2787-E2796).

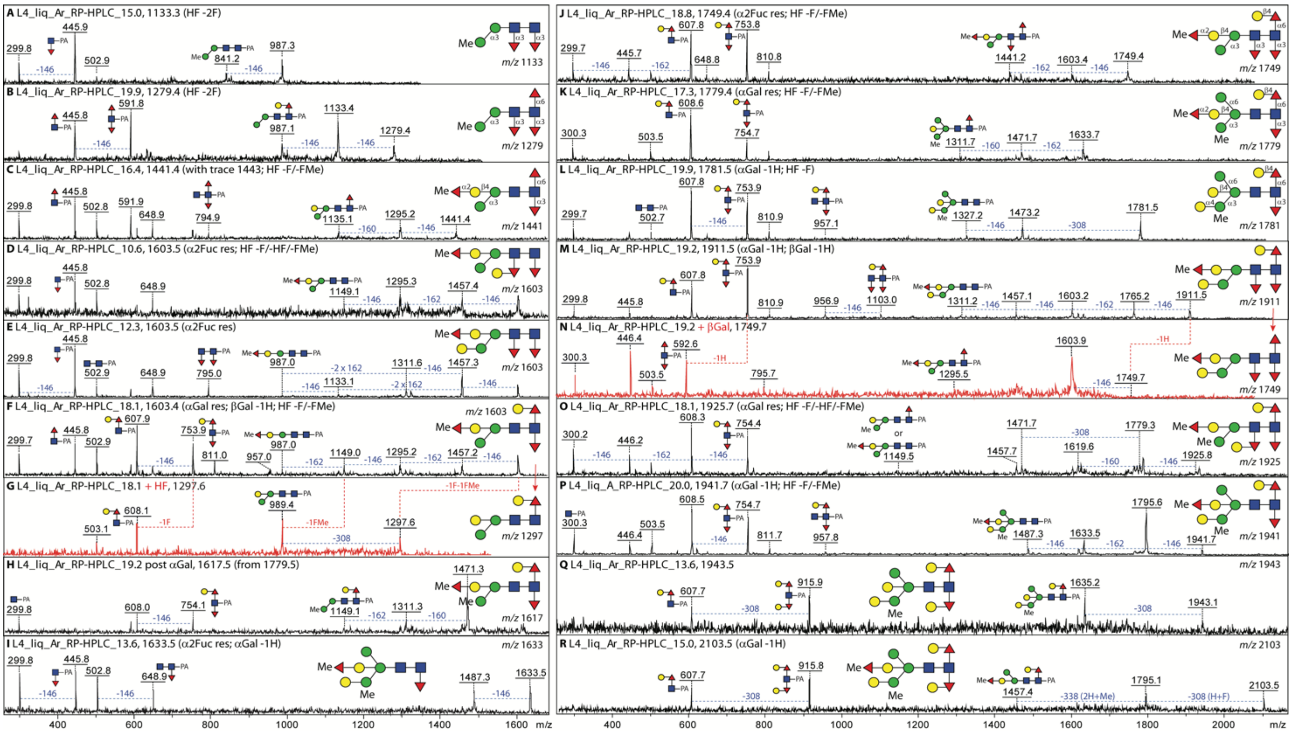

Supplementary Figure S12: Abundance of different categories N-glycans in liquid- and plate-grown L4 larvae. Percentage occurrence of individual glycans from the second L4 liquid and plate preparations was calculated on the basis of integrated fluorescence of RP-HPLC fractions divided by relative MALDI-TOF-MS peak areas (see **Supplementary Table S1**); these values do not account for differences in ionization, thereby the phosphorylcholine-modified structures are probably overestimated. The N-glycans were categorised based on structural type and the occurrences summed; α-galactosylated glycans are found amongst the bisected and methylated categories in both the PNGase F- and PNGase Ar-released subglycomes and the percentage occurrence of these is separately summed together with example structures of selected categories.

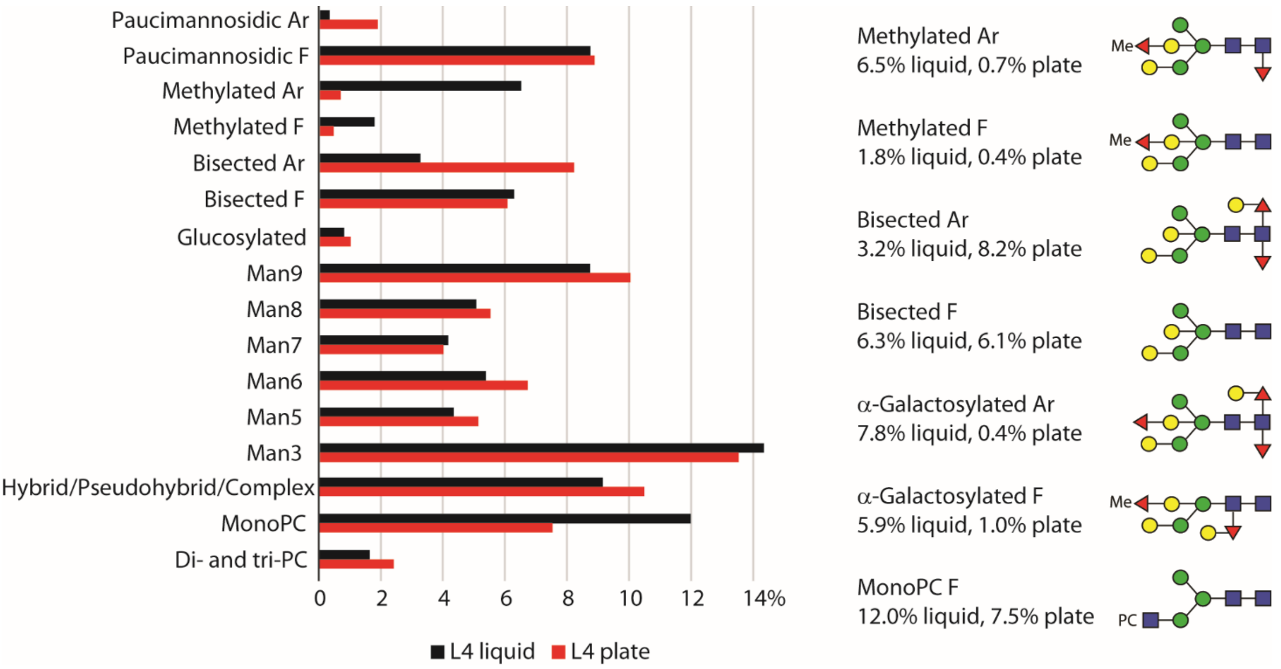

Supplementary Figure S13: LC-MS/MS analyses of four example neutral O-glycans. Peptic glycopeptides remaining after PNGase A/F digestion from a *C. elegans* mixed culture were subject to reductive β-elimination and analysed without further derivatization by LC-ESI-MS (see also **Supplementary Table 2**). The fragmentation patterns allow the putative structures of $Hex_2HexNAc_1$ (*m/z* 546), $Fuc_1Hex_2HexNAc_1$ (*m/z* 692), $Hex_4HexNAc_1$ (*m/z* 870) and $HexA_1Hex_3HexNAc_1$ (*m/z* 884) to be proposed and are compatible with either the specificity of the GLY-1 β1,6-glucosyltransferase (Warren *et al*., 2002, *Glycobiology*, 12, 8G-9G) or previous NMR data on O-glycans of the nematode indicating modification of the core 1 structure by glucose and glucuronic acid residues (Guérardel *et al*., 2001, *Biochem. J.*, 357, 167-82).

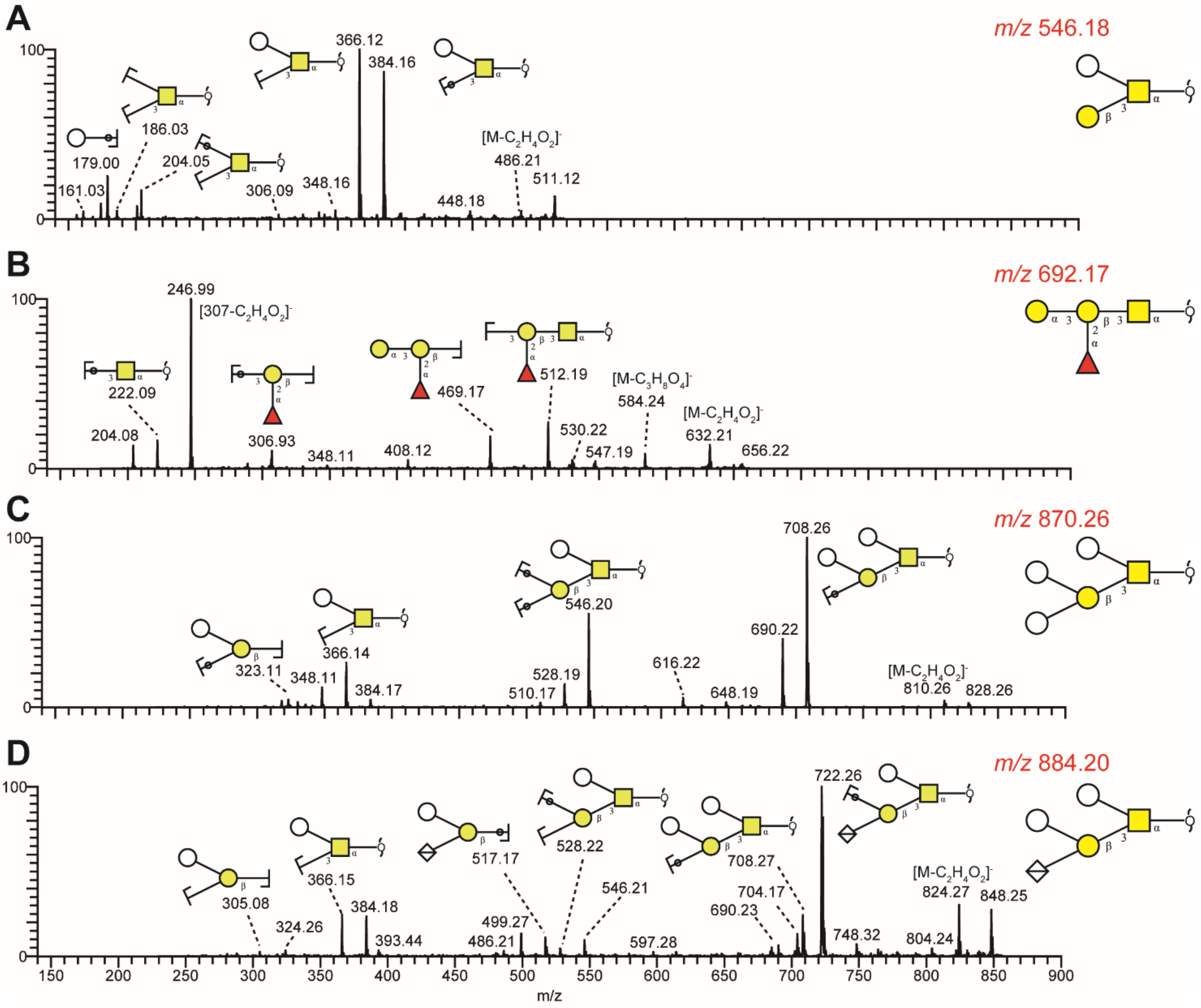

Supplementary Figure S14: Transcriptomic data for genes with proven function in N-glycan modification. Plots for twelve genes encoding proteins with known biochemical function, specifically Golgi/ER class I α1,2-mannosidases, GlcNAc-transferases I, II and V, Golgi α-mannosidase II, Golgi β-hexosaminidases, core α-fucosyltransferases and the Fuc-modifying β-galactosyltransferase (*mans-1, mans-2, mans-3, mans-4, gly-2, gly-12, gly-13, gly-14, gly-20, aman-2, hex-2, hex-3, fut-1, fut-6, fut-8* and *galt-1*) were downloaded from http://mbb-apps.dc.sfu.ca/gexplore/gexplore_search_expression.html and result from RNA-seq analysis for a detailed embryonic time course as well as larval stages L1-L4 and young adults (YA). Y-axes are in dcpm (depth of coverage per base per million reads). In most cases, there is an increase in expression during embryonal development, followed by a plateau or even reduction in later stages, whereby the increase in expression of the core α1,3-fucosyltransferase *fut-1* and *fut-6* genes is delayed as compared to that of the core α1,6-fucosyl-transferase *fut-8* gene; exceptions to this are *mans-1*, *mans-4*, *gly-14*, *gly-20* and *hex-3* for which there appears a fall in expression during embryonal development, followed by a later increase. Functional redundancy *gly-12-14*, *hex-2-3*, *mans-1-4* genes in terms of enzymatic activity and/or glycomic impact has been established.

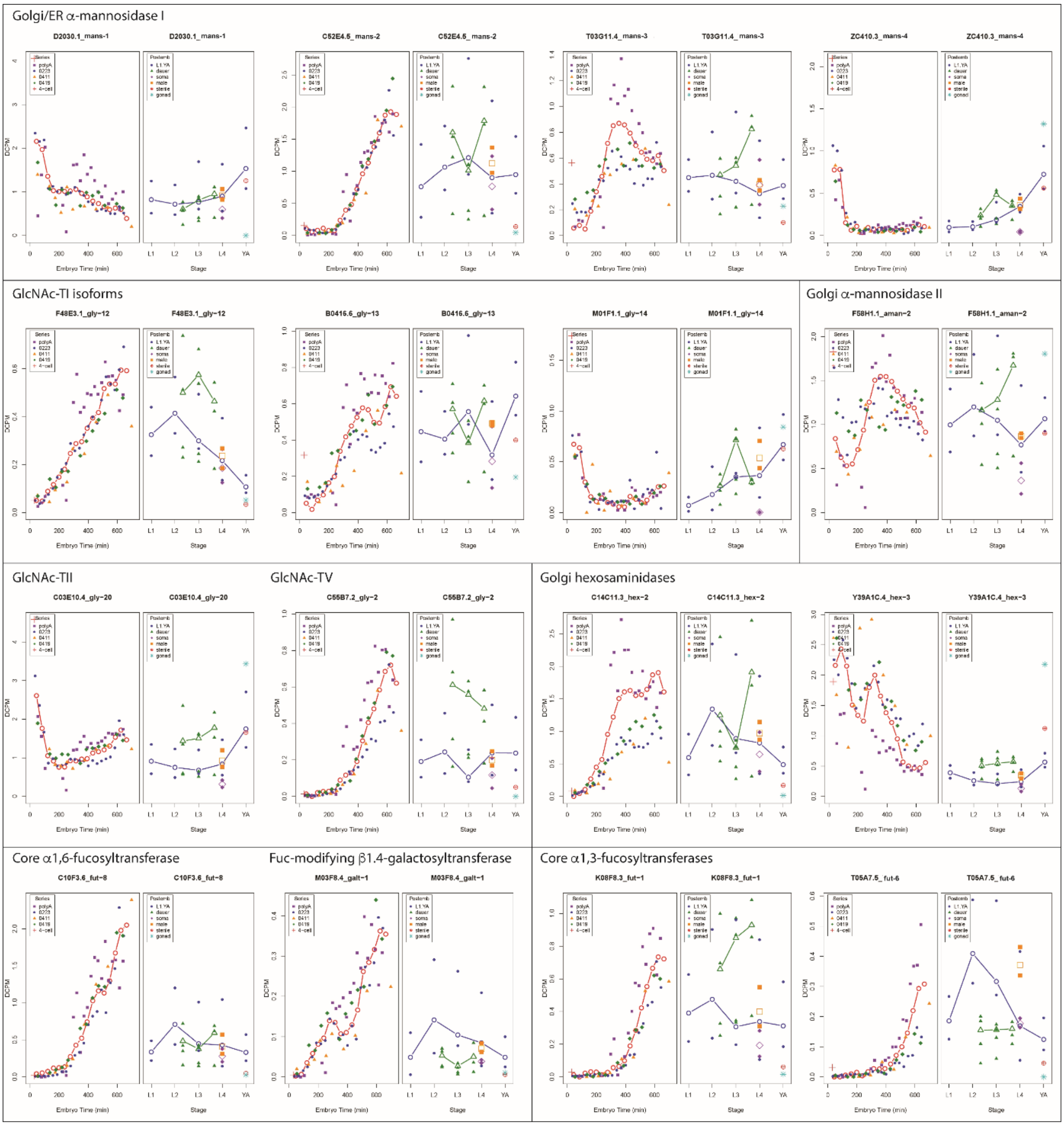

Supplementary Figure S15: Potential glycosylation-relevant gene clusters. Depicted are three regions of the *C. elegans* genome (two regions of chromosome V and one of chromosome I) with consecutive potential glycosylation-relevant genes. The direction of transcription is shown as are the reading frame numbers and gene name. The type of encoded protein (glycosyltransferase with CAZy GT family number, carbohydrate sulphotransferase, methyltransferase or domain of unknown function (DUF)) is shown underneath the reading frame. Most of these reading frames meet criteria for potential Golgi localization (300-600 amino acids and hydrophobic domain close to the terminus) and were included in the re-analysis of transcriptome data. CAZy family members are as follows: GT11, α1,2-fucosyltransferase and *fut-2* homologues, GT31, core 1 β1,3-galactosyltransferase homologues, GT14, *gly-1* β1,6-glucosyltransferase homologues, GT43 β1,3-glucuronyltransferase or β1,4-xylosyltransferase homologue *glct-3*, GT49 β1,3-glucuronyltransferase homologue *bgnt-1.7* and GT92 fucose-modifying β1,4-galactosyltransferase *galt-1* homologues (see www.cazy.org; Drula *et al.*, 2022, *Nucleic Acids Res.*, 50, D571-D577). The K06H6 'cluster' also contains a nucleotide sugar transporter gene *nstp-7*, while the transcription factor NHR-77 (a nuclear hormone receptor homologue which according to modENCODE CHIP-seq data binds 5'-regions of some glycosidase and glycosyltransferase genes) is encoded within the T15D6 'cluster'.

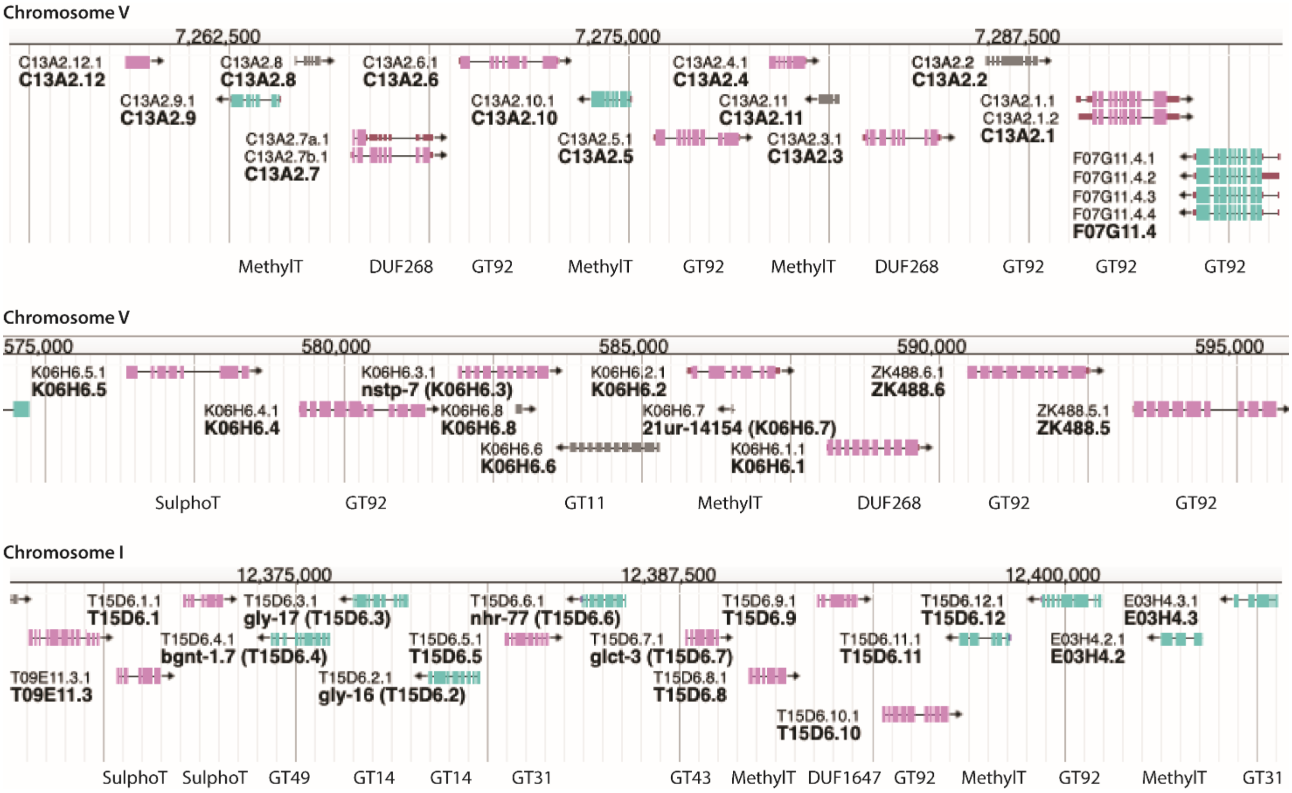

Supplementary Figure S16: Heatmap of 700 genes with single predicted transmembrane domains and cluster analysis of 285 genes with proven or potential roles as Golgi enzymes (see additional supplementary file). **(A)** Heatmap of RNA-seq transcriptomic data (embryonal time points and L1-L4, dauer and adult) for 700 genes encoding proteins of between 300 and 600 amino acids and with a single predicted hydrophobic domain (i.e., with properties akin to those of known Golgi enzymes). **(B)** Corrplot cluster analysis of RNA-seq transcriptomic data (L1-L4, dauer, adult and aggregated data) for 285 genes encoding proteins of either known roles in glycosylation and/or present in potential glycogene clusters and/or member of CAZy families GT2 (β1,4-hexosyltransferases; including *bre-3*), GT7 (β1,4-hexosyltransferase homologues; *bre-4*, *sqv-3* and *ngat-1*), GT10 (α1,3-fucosyltransferase homologues; including *fut-1* and *fut-6*), GT11 (α1,2-fucosyltransferase homologues; including *fut-2*), GT13 (β1,2-*N*-acetylglucosaminyltransferase I; *gly-12* to *gly-14*), GT14 (β1,6-hexosyltransferases and peptide O-xylosyltransferase; including *gly-1* and *sqv-6*), GT16 (β1,2-*N*-acetylglucosaminyltransferase II; *gly-20*), GT18 (β1,6-*N*-acetylglucosaminyltransferase V; *gly-2*), GT23 (α1,6-fucosyltransferase; *fut-8*), GT27 (polypeptide α-*N*-acetylgalactosaminyltransferases; *gly-3* to *gly-11*), GT31 (β1,3-hexosyltransferase homologues; including *bre-2*, *bre-5*, *sqv-2, sqv-5* and core 1 β1,3-galactosyltransferase), GT43 (β1,3-glucuronyltransferase homologues; including *sqv-8*), GT47 (β1,4-glucuronyltransferase homologues; including *rib-1* and *rib-2*), GT49 (β1,3-hexosyltransferase homologues), GT92 (β1,4-galactosyltransferase homologues; including *galt-1*), GH20 (β-*N*-acetylhexosaminidases; *hex-1* to *hex-5*), GH38 (class II α-mannosidases; including *aman-2*) or GH47 (class I α-mannosidases; *mans-1* to *mans-4*). This figure corresponds to ***Figure 9*** in the main text, but shows all gene names.
