# Supplementary figures and images for: Increasing Complexity of the N-Glycome During Caenorhabditis Development
Source: Mol Cell Proteomics. 2023 Jan 28;22(3):100505. doi: 10.1016/j.mcpro.2023.100505 (PMC7614267; doi:10.1016/j.mcpro.2023.100505)

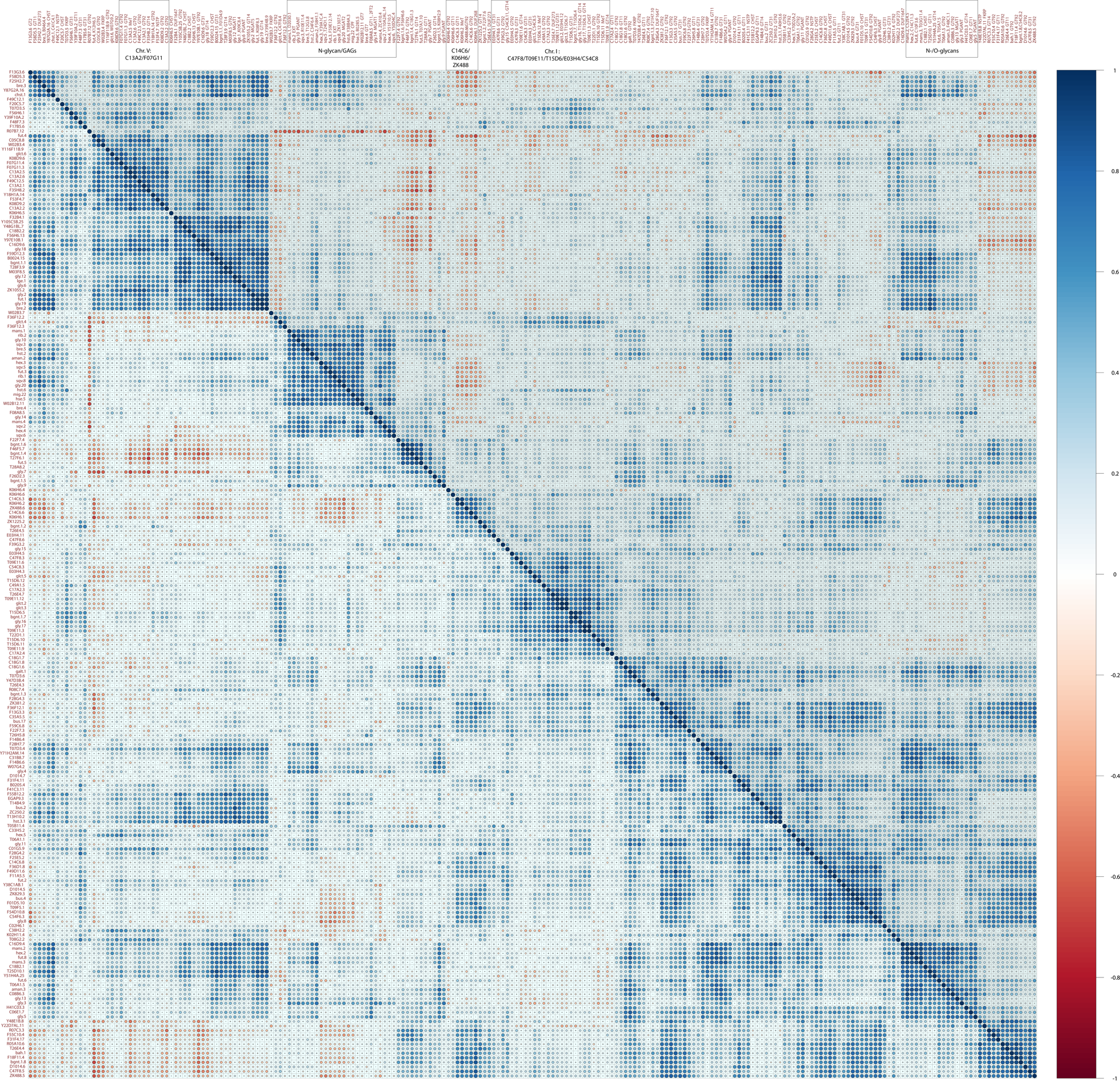

Supplement: SuppFigure16B_corrplot_adult [file mmc3.pdf]
