# Supplementary material for: Increasing Complexity of the N-Glycome During Caenorhabditis Development
Source: Mol Cell Proteomics. 2023 Jan 28;22(3):100505. doi: 10.1016/j.mcpro.2023.100505 (PMC7614267; doi:10.1016/j.mcpro.2023.100505)

Heatmap of transcriptome C. elegans embryo and adult form

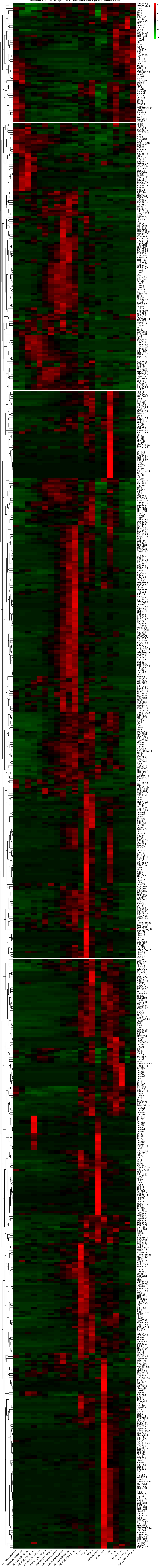

Supplement: SuppFigure16A_transcriptome [file mmc2.pdf]
